# New tricks of well-known aminoazoles in isocyanide-based multicomponent reactions and antibacterial activity of the compounds synthesized

**DOI:** 10.3762/bjoc.13.104

**Published:** 2017-05-31

**Authors:** Maryna V Murlykina, Maryna N Kornet, Sergey M Desenko, Svetlana V Shishkina, Oleg V Shishkin, Aleksander A Brazhko, Vladimir I Musatov, Erik V Van der Eycken, Valentin A Chebanov

**Affiliations:** 1 Division of Chemistry of Functional Materials, State Scientific Institution “Institute for Single Crystals” of National Academy of Sciences of Ukraine, Nauky Ave., 60, 61001, Kharkiv, Ukraine; 2 Laboratory for Organic & Microwave-Assisted Chemistry (LOMAC), KU Leuven, Celestijnenlaan 200F, B-3001, Leuven, Belgium; 3 Laboratory of Biotechnology of Physiologically Active Substances, Zaporizhzhya National University, Zhukovsky str., 66, 69600, Zaporizhzhya, Ukraine; 4 Faculty of Chemistry, V. N. Karazin Kharkiv National University, Svobody sq., 4, 61077, Kharkiv, Ukraine

**Keywords:** 3-amino-5-methylisoxazole, 5-amino-*N*-aryl-1*H*-pyrazole-4-carboxamides, antibacterial activity, Groebke–Blackburn–Bienaymé reaction, isocyanide Ugi reaction

## Abstract

The well-known aminoazoles, 3-amino-5-methylisoxazole and 5-amino-*N*-aryl-1*H*-pyrazole-4-carboxamides, were studied as an amine component in Ugi and Groebke–Blackburn–Bienaymé multicomponent reactions. The first example of an application of aminoazoles in an Ugi four-component reaction was discovered and novel features of a Groebke–Blackburn–Bienaymé cyclocondensation are established and discussed. The heterocycles obtained were evaluated for their antibacterial activity and several of them demonstrated a weak antimicrobial effect, but for most of the compounds a 30–50% increase in biomass of Gram-positive strains (mainly *B. subtilis*) compared to control was observed.

## Introduction

An intensive progress in pharmaceutical and medicinal chemistry, as well as in the generation and improvement of medicinal technologies has led to defeating a wide scope of diseases. However, we are still facing the problem of untreated ones, together with the appearance of unknown disorders and the dramatical growth of antimicrobial resistance caused by the continuous evolution of microorganisms [1–5]. Therefore, there is urgency in careful screening the chemical space with the aim of finding new biologically active structures. Modern chemistry offers several approaches, for instance, diversity oriented synthesis (DOS) for the generation of diverse compound libraries [6–8]. From this point of view, multicomponent reactions (MCRs), including isocyanide-based MCRs as the Ugi four-component reaction (Ugi-4CR) and the Groebke–Blackburn–Bienaymé reaction (GBB-3CR) in combination with post-cyclizations are powerful tools to access diversity as well as complexity in a one-pot procedure; in this way they largely cover the available chemical space [9–26]. The imidazoheterocyclic scaffold represents a promising area for the discovery of novel synthetic drug molecules [27–52]. Particularly, there are several drugs containing the imidazo[1,2-*a*]pyridine moiety such as zolpidem (treatment of insomnia) and olprinone (cardiotonic drug) and a lot of compounds in biological testing and preclinical evaluation such as soraprazan (clinical antiulcer compound), necopidem (sedative effect), and saripidem (anxiolytic) [27]. The activity of different imidazoheterocycles was also studied against migraine [30], gastric [31–32], heart [34–36], viral diseases [37–41] and an array of neurological syndromes [52]. The imidazo[1,2-*b*]pyrazole core shows also a pharmacological potential. Among others, anti-inflammatory [28,43], antiviral [39,44], antidiabetic [45] effects and cancer cell growth-inhibitory features should be mentioned [29,46–48].

Ugi-4CR has been applied in the synthesis of natural products, as bicyclomycin, furanomycin, penicillin etc. [53]. The high combinatorial potential of Ugi-4CR together with the ability to incorporate a variety of functionalities and modifications extend its application for the generation of organic compound libraries, following hit-to-lead optimization, choosing the hit structure and final marketed drug production [54–58]. Moreover, it has been acknowledged that the combination of two privileged scaffolds in a single molecule (e.g., the combination of a peptidomimetic structure with an azole fragment [59]) potentially creates more active, new entities with unusual bioproperties [20,60–61]. In addition, the application of polyfunctional reagents in Ugi-4CR opens ways to different post-cyclization reactions, thereby broadening the scope. Thus the Ugi-4CR involving substituted propiolic acids, can be followed by electrophilic ipso-iodocyclization [62] or transition-metal-initiated [63–68] and metal-free cyclizations [69–70].

**Scheme 1:**
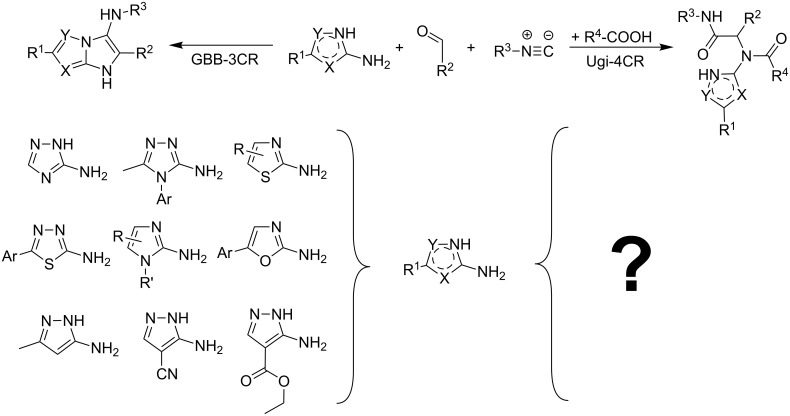
Aminoazoles in GBB-3CR and Ugi-4CR.

There are examples of using aminoazoles as an amine component in GBB-3CR (Scheme 1). They mostly involve different substituted 3-amino-1,2,4-triazoles [71–75] and 2-amino(benzo)thiazoles [71–72,76–88]. Several publications deal with 2-amino-1,3,4-thiadiazoles [71,83–84,89–90], 2-aminoimidazoles [71–72,91–92], 2-aminoxazoles [71] and 1,2,5-oxadiazole-3,4-diamine [93] with the formation of imidazoazoles. Among the pyrazoles only 5-amino-3-methylpyrazole, 5-aminopyrazole-4-carbonitrile and ethyl 5-aminopyrazole-4-carboxylate are described in GBB-3CR [29,71,83–84,94–96]. To the best of our knowledge, there is no information about the reactivity of aminoazoles as an amine component in Ugi-4CR.

Taking into account the above-mentioned facts, several aminoazoles, whose reactivity in isocyanide-based reactions had not been studied yet, were examined as an amine component in Ugi-4CR and GBB-3CR. The generated compounds were screened for their biological activity towards *Bacillus subtilis*, *Staphylococcus aureus*, *Escherichia coli* and *Pseudomonas aeruginosa.*

## Results and Discussion

**Scheme 2:**
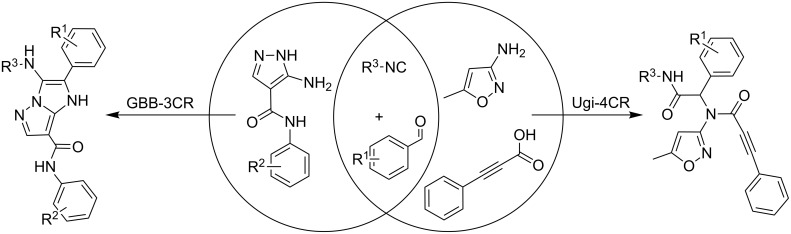
Reactivity of 5-amino-*N*-aryl-1*H*-pyrazole-4-carboxamide and 3-amino-5-methylisoxazole in GBB-3CR and Ugi-4CR.

Since aminoazoles contain an exocyclic NH_2_ group and an endocyclic nucleophilic center, they can act both as primary amines and as 1,3-binucleophiles, therefore, their treatments with isocyanides, aldehydes and carboxylic acids may proceed either as Ugi-4CR (aminoazole – primary amine, acid – reagent) or as GBB-3CR (aminoazole – 1,3-binucleophile, acid – catalyst). Literature data indicate [14,25,59,97–101] that 5-aminopyrazoles bearing in the fourth position electron-withdrawing substituents like carboxamide, carboxylate or a carbonitrile group, posses chemical properties being different from other 5-aminopyrazoles but sometimes similar to 3-amino-1,2,4-triazole that was described as a component of GBB-3CR earlier [71–75]. Therefore, the first type of aminoazoles studied in our work was 5-amino-*N*-aryl-1*H*-pyrazole-4-carboxamide that showed 1,3-binucleophile properties in the condensation with aromatic aldehydes and alkylisocyanides giving the product resulting from GBB-3CR. On the other hand, 3-amino-5-methylisoxazole in MCRs often acted as primary amine [102–106], that was confirmed in our case by treatment with aromatic aldehydes, alkylisocyanides and phenylpropiolic acid resulting in the corresponding products of the Ugi-4CR (Scheme 2).

The scope and limitations of isocyanide-based reactions involving 5-amino-*N*-aryl-1*H*-pyrazole-4-carboxamides and 3-amino-5-methylisoxazole were studied in detail. It was established that the optimal reaction conditions of a GBB-3CR involving 5-amino-*N*-aryl-1*H*-pyrazole-4-carboxamides **2a–d** were different depending on the other substrates. Particularly, the condensation involving *tert*-butylisocyanide (**3a**) and aldehydes **1a–e** bearing electron-donating substituents was effectively carried out in EtOH/H_2_O mixture with TFA (10 mol %) at room temperature for 24 h (method A) resulting in the formation of imidazopyrazoles **4a–m** (Table 1, entries 1–13).

**Table 1:** CBB-3CR involving *tert*-butylisocyanide.

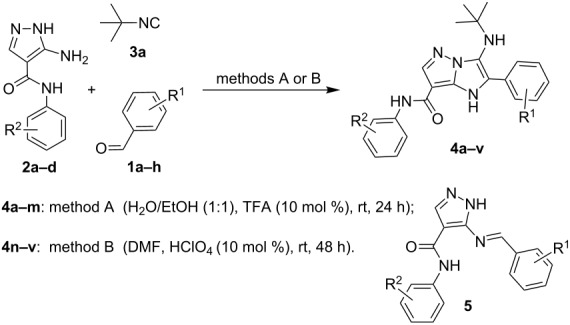							

Entry	Starting materials				Method	Product	Yield, %
	Aldehydes	R^1^	Aminopyrazoles	R^2^			

1	**1a**	H	**2a**	4-F	A	**4a**	54
2	**1b**	2-CH_3_O	**2a**	4-F	A	**4b**	75
3	**1c**	3-CH_3_O	**2a**	4-F	A	**4c**	77
4	**1d**	4-CH_3_O	**2a**	4-F	A	**4d**	75
5	**1e**	4-Cl	**2a**	4-F	A	**4e**	72
6	**1b**	2-CH_3_O	**2b**	3-F	A	**4f**	83
7	**1d**	4-CH_3_O	**2b**	3-F	A	**4g**	64
8	**1e**	4-Cl	**2b**	3-F	A	**4h**	89
9	**1b**	2-CH_3_O	**2c**	2-CH_2_CH_3_	A	**4i**	82
10	**1d**	4-CH_3_O	**2c**	2-CH_2_CH_3_	A	**4j**	64
11	**1e**	4-Cl	**2c**	2-CH_2_CH_3_	A	**4k**	66
12	**1b**	2-CH_3_O	**2d**	4-CH_2_CH_3_	A	**4l**	85
13	**1e**	4-Cl	**2d**	4-CH_2_CH_3_	A	**4m**	53
14	**1f**	4-CO_2_CH_3_	**2a**	4-F	B	**4n**	85
15	**1g**	4-NO_2_	**2a**	4-F	B	**4o**	87
16	**1h**	4-CN	**2a**	4-F	B	**4p**	90
17	**1f**	4-CO_2_CH_3_	**2b**	3-F	B	**4q**	88
18	**1g**	4-NO_2_	**2b**	3-F	B	**4r**	87
19	**1h**	4-CN	**2b**	3-F	B	**4s**	82
20	**1f**	4-CO_2_CH_3_	**2c**	2-CH_2_CH_3_	B	**4t**	61
21	**1g**	4-NO_2_	**2c**	2-CH_2_CH_3_	B	**4u**	79
22	**1h**	4-CN	**2c**	2-CH_2_CH_3_	B	**4v**	83

This condensation was also carried out in TFE or MeOH with addition of HClO_4_ (10 mol %), but a significant amount of Schiff base **5** (Table 1) was observed in this case. The reaction involving aldehydes **1f–h** bearing strong electron-withdrawing groups under all the abovementioned conditions allowed isolating a mixture of imidazopyrazoles **4n–v** with a large quantity of Schiff bases **5**.

Therefore, the conditions for the synthesis of compounds **4n–v** were optimized employing 5-amino-*N*-(3-fluorophenyl)-1*H*-pyrazole-4-carboxamide (**2b**), methyl benzaldehyde-4-carboxylate (**1f**) and *tert*-butylisocyanide (**3a**, Table 2).

**Table 2:** Optimization of the reaction conditions for the synthesis of compound **4q**.

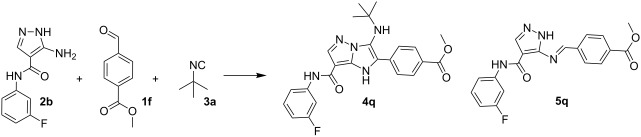					

Entry	Solvent, acid	Time, hours	*T,* °C	Products (ratio)	Yield, %^a^

1	TFE, HClO_4_, (10 mol %)	72	25	**4** + **5** (2:1)	–
2	EtOH/H_2_O (1:1), TFA, (10 mol %)	24	25	**4** + **5** (1:1)	–
3	EtOH/H_2_O (1:1), TFA, (20 mol %)	24	25	**4** + **5** (1:1)	–
4	EtOH/H_2_O (1:1), TFA, (10 mol %)	12	80	**4** + **5** (2:1)	–
5	EtOH/H_2_O (1:1), TFA, (10 mol %)	0.3^b^	140	**4** + **5** (4:5)	–
6	EtOH/H_2_O (1:1), TFA, (10 mol %)	2^c^	25	**4** + **5**^d^	–
7	EtOH/H_2_O (1:1), TFA, (10 mol %)	48	85	**4**	56
8	EtOH/H_2_O (1:1), TFA, (10 mol %)	5^b^	140	**4** + **5** (2:3)	–
9	DMF, HClO_4_, (10 mol %)	48	25	**4**	88

^a^The yields are indicated for compound **4** and are not calculated for the mixtures; ^b^upon MW irradiation; ^c^upon US irradiation; ^d^in a mixture with starting materials and undetected impurities.

Obviously, this reaction requires a longer reaction time (min. 48 h) and a moderate temperature (not more than 85 °C) to avoid tarring. Thus, after 48 h of heating (oil bath) at 85 °C the starting materials **1f**, **2b** and **3a** in EtOH/H_2_O with TFA (10 mol %), imidazopyrazole **4q** was isolated in 56% yield. However, the mother liquor still contained unreacted Shiff base **5q** (entry 7, Table 2 ). On the other hand, using DMF/HClO_4_ (10 mol %, method B) allowed obtaining the target compound **4q** in 88 % yield with no impurities (entry 9, Table 2).

Similarly the reaction of 5-amino-*N*-(4-fluorophenyl)-1*H*-pyrazole-4-carboxamide (**2a**) with 4-nitrobenzaldehyde (**1g**) and *tert*-butylisocyanide (**3a**) in EtOH/H_2_O with TFA (10 mol %) also led to Shiff base **5o** while stirring the starting materials in DMF-HClO_4_ (10 mol %) for 48 h gave compound **4o** (Table 3).

**Table 3:** Optimization of the reaction conditions for the synthesis of compound **4o**.

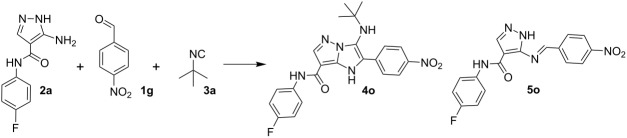					

Entry	Solvent	Time, hours	*T,* °C	Main product	Yield, (%)^a^

1	EtOH/H_2_O (1:1), TFA, (10 mol %)	72	25	**5**^b^	–
2	EtOH/H_2_O (1:1), TFA, (10 mol %)	2	140^c^	**5**^b^	–
3	EtOH/H_2_O (1:1), TFA, (10 mol %)	72	90	**5**^b^	–
4	DMF, HClO_4_, (10 mol %)	48	25	**4**	87

^a^The yields are indicated for compound **4o**; ^b^in a mixture with starting materials and undetected impurities; ^c^upon MW irradiation.

It should be noted that the conditions of method B are also suitable for obtaining imidazopyrazoles **4a–m** in comparatively high yields; however, the synthesis in EtOH/H_2_O medium is preferred from the point of view of green chemistry. Thereby, the optimal methodology for obtaining compounds **4a–m** is the synthesis according to the method A (H_2_O/EtOH (1:1), TFA (10 mol %), rt, 24 h) while for compounds **4n–v** method B (DMF, HClO_4_ (10 mol %), rt, 48 h) proved to be superior (Table 1, entries 14–22).

We presume that such difference in the outcome of GBB-3CR depending on the substitution pattern in the carbonyl component is related with the ability of the intermediate Schiff bases to be protonated as well as with their solubility. In case of the presence of electron-withdrawing substituents in the aldehyde the corresponding Schiff bases **5** are less soluble and less basic. DMF increases solubility of imines **5**, while the application of strong acid (HClO_4_) promotes Shiff bases protonation.

Phenylpyruvic acid (**1'**) was also applied as carbonyl component in GBB-3CR to obtain imidazopyrazoles having a carboxylic group. However, the process of decarboxylation took place in the reaction and 1-*H*-imidazopyrazole carboxamides **4w,x** were isolated as the sole reaction products (Table 4).

**Table 4:** Phenylpyruvic acid in GBB-3CR.

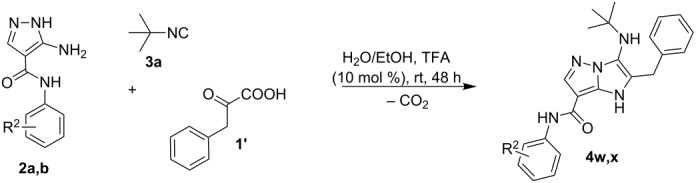				

Entry	Starting materials		Product	Yield, %
	Aminopyrazoles	R^2^		

1	**2a**	4-F	**4w**	48
2	**2b**	3-F	**4x**	42

When replacing *tert*-butylisocyanide (**3a**) with ethyl 2-isocyanoacetate (**3b**), imidazo[1,2-*b*]pyrazole-7-carboxamides **6a–h** were isolated. The condensation proceeded well in TFE with the addition of HClO_4_ (10 mol %) upon stirring for 24 h (Table 5). On the other hand, condensation of the starting reagents **1d**–**g** with **2a,b** and **3b** in EtOH/H_2_O (1:1) with TFA (10 mol %) resulted in the formation of the target products **6** in a mixture with a substantial amount of Schiff bases **5**. Moreover, only imines **5** with impurities of the starting compounds were isolated when applying DMF with HClO_4_.

**Table 5:** GBB-3CR involving ethyl 2-isocyanoacetate.

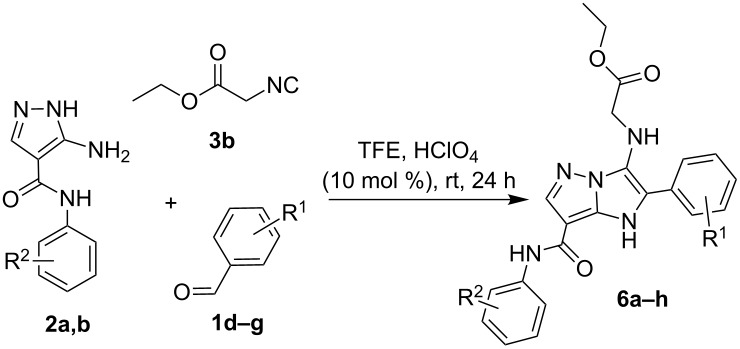						

Entry	Starting materials				Product	Yield, %
	Aldehydes	R^1^	Aminopyrazoles	R^2^		

1	**1d**	4-CH_3_O	**2a**	4-F	**6a**	77
2	**1e**	4-Cl	**2a**	4-F	**6b**	95
3	**1f**	4-CO_2_CH_3_	**2a**	4-F	**6c**	67
4	**1g**	4-NO_2_	**2a**	4-F	**6d**	85
5	**1d**	4-CH_3_O	**2b**	3-F	**6e**	78
6	**1e**	4-Cl	**2b**	3-F	**6f**	84
7	**1f**	4-CO_2_CH_3_	**2b**	3-F	**6g**	82
8	**1g**	4-NO_2_	**2b**	3-F	**6h**	80

Interestingly, in case of ethyl 2-isocyanoacetate (**3b**) the reaction proceeded equally well regardless of the substituent in the aldehyde. This can be connected with an increased reactivity of ethyl 2-isocyanoacetate (**3b**) compared to *tert*-butylisocyanide (**3a**) due to sterical reasons thus leveling the influence of the solubility factor of imines **5**.

As it has been already mentioned above, in contrast to 5-amino-*N*-aryl-1*H*-pyrazole-4-carboxamides **2**, 3-amino-5-methylisoxazole (**7**) exhibited only properties of primary amines that allowed using it as an amine component in Ugi-4CR. Particularly, its reaction with aromatic aldehydes **1a–h**, phenylpropiolic acid (**8**) and *tert*-butylisocyanide (**3a**) gave peptidomimetics **9** under stirring the starting reagents in MeOH at room temperature for 24 h. In the presence of strong electron-withdrawing substituents as nitro or cyano groups in *para*-position of the aldehyde, despite the variation of the reaction conditions, only imines **10** were isolated as the major products. In case of 4-cyanobenzaldehyde (**1h**) we managed to isolate Ugi product **9g** in a low yield of 18% (Table 6).

**Table 6:** Ugi-4CR involving 3-amino-5-methylisoxazole.

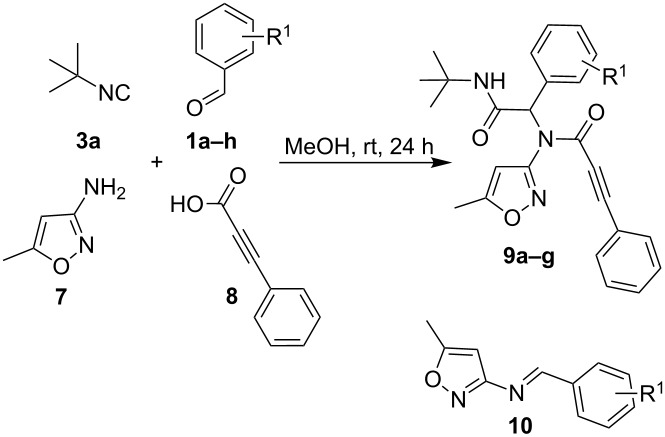				

Entry	Starting materials		Product	Yield^b^, %
	Aldehydes	R^1^		

1	**1a**	H	**9a**	49
2	**1b**	2-CH_3_O	**9b**	67
3	**1c**	3-CH_3_O	**9c**	51
4	**1d**	4-CH_3_O	**9d**	73
5	**1e**	4-Cl	**9e**	43
6	**1f**	4-CO_2_CH_3_	**9f**	43
7	**1g**	4-NO_2_	**10g**^a^	–^b^
8	**1h**	4-CN	**9h**	18

^a^**9g** was not isolated; ^b^the yields are indicated for compounds **9**.

It should be noted that 5-amino-*N*-aryl-1*H*-pyrazole-4-carboxamides **2** were also introduced into Ugi-4CR with aromatic aldehydes **1**, alkylisocyanides **3** and phenylpropiolic acid **8**; however, the acid **8** acted as a catalyst favouring the formation of GBB-3CR-products **4**. The attempts to carry out GBB-3CR involving 3-amino-5-methylisoxazole (**7**) according to the elaborated procedures (Table 1, methods A or B) as well as under other conditions were not successful.

### Structure elucidation

The purity and structures of the heterocycles obtained were established by means of mass spectrometry (including HRMS), NMR spectroscopy and X-ray diffraction study.

The ^1^H NMR spectra of imidazo[1,2-*b*]pyrazole-7-carboxamides **4** exhibit a broad signal for the NH group in the position 1 at ca. 11.8 ppm, a broad signal for the carboxamide NH at ≈9.5 ppm, a singlet for pyrazole CH in the position 6 at ≈8.2 ppm, a broad signal for the NH group near the position 3 at ≈5.1 ppm, a singlet for *tert*-butyl CH_3_ groups at ≈1.0 ppm, resonances for the aromatic protons around 6.9–8.2 ppm as well as signals for other substituents. In case of imidazo[1,2-*b*]pyrazole-7-carboxamides **4w**,**x** a broad signal for the *tert*-butyl NH group is shifted upfield to 4.2 ppm and an additional singlet for the benzyl CH_2_ group is present at 3.9 ppm.

The ^1^H NMR spectra of imidazo[1,2-*b*]pyrazole-7-carboxamides **6** exhibit a broad signal for the NH group in the position 1 at ≈11.8 ppm, a broad signal for the carboxamide NH group at ≈9.6 ppm, a singlet for the pyrazole CH in the position 6 at ≈8.2 ppm, a broad signal for the NH group near the position 3 at ≈5.7 ppm, a singlet for the CH_2_ group in the acetate moiety at ≈4.2 ppm, peaks for the ethoxy group: a quartet for CH_2_ group at ≈4.0 ppm and a triplet for the CH_3_ group at ≈1.0 ppm, peaks for the aromatic protons around 6.7–7.7 ppm as well as signals for other substituents.

The ^1^H NMR spectra of *N*-(1-arylethyl-2-(*tert*-butylamino)-2-oxo)-*N*-(5-methylisoxazol-3-yl)-3-phenylpropiolamides **9** exhibit a broad signal for the amide NH group at ≈7.9 ppm, singlet for the isoxazole CH at ≈6.3 ppm, a singlet for the CH group in the position 1 at ≈6.0 ppm, a singlet for the isoxazole CH_3_ group at ≈2.3 ppm, a singlet for the *tert*-butyl CH_3_ groups at ≈1.2 ppm, peaks for the aromatic protons around 6.8–7.5 ppm as well as signals for other substituents.

**Figure 1:**
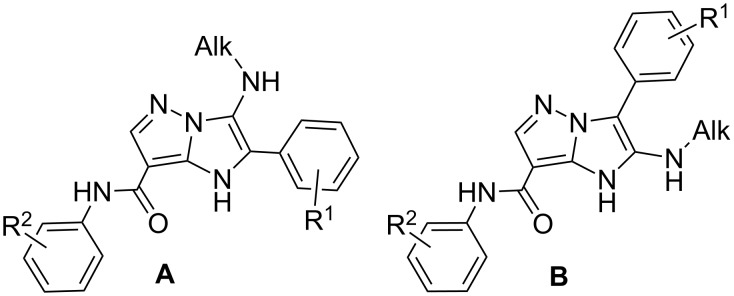
Alternative structures **A** and **B** for compounds **4** and **6**.

As it was found earlier for 2-aminopyrimidines [107–109] GBB-3CR may lead to the formation of two positional isomers **A** and **B** (Figure 1).

**Figure 2:**
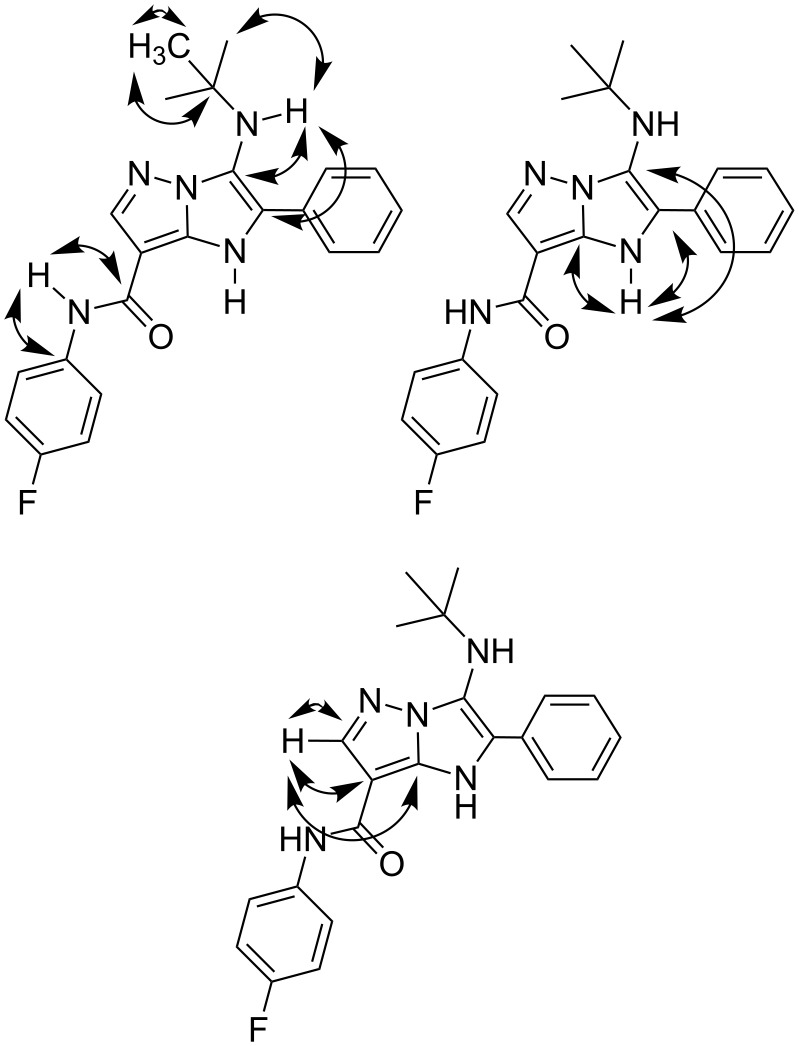
Selected data of HSQC and HMBC experiments for compound **4a**.

Experiment with D_2_O allowed to identify the signals of NH protons while the HSQC spectrum showed the correlations between the signals of protons and corresponding carbon atoms (in the position 6 and in *tert*-butyl group). The correlations between the signals of NH protons and corresponding carbon atoms (through two and three bonds, Figure 2) allowed final distinguishing the shifts of three NH groups signals in ^1^H NMR spectra.

**Figure 3:**
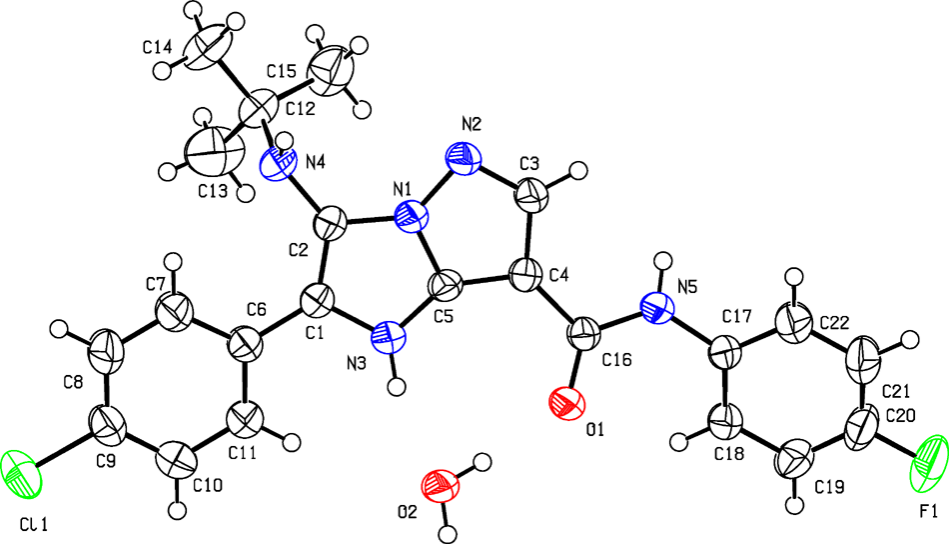
Molecular structure of 3-(*tert*-butylamino)-2-(4-chlorophenyl)-*N*-(4-fluorophenyl)-1*H*-imidazo[1,2-*b*]pyrazole-7-carboxamide (**4e**) (X-ray diffraction data). Non-hydrogen atoms are presented as thermal ellipsoids with 50% probability.

However, the final assignment of the structure **A** for heterocycles **4** was made with the help of X-ray analysis (Figure 3).

**Figure 4:**
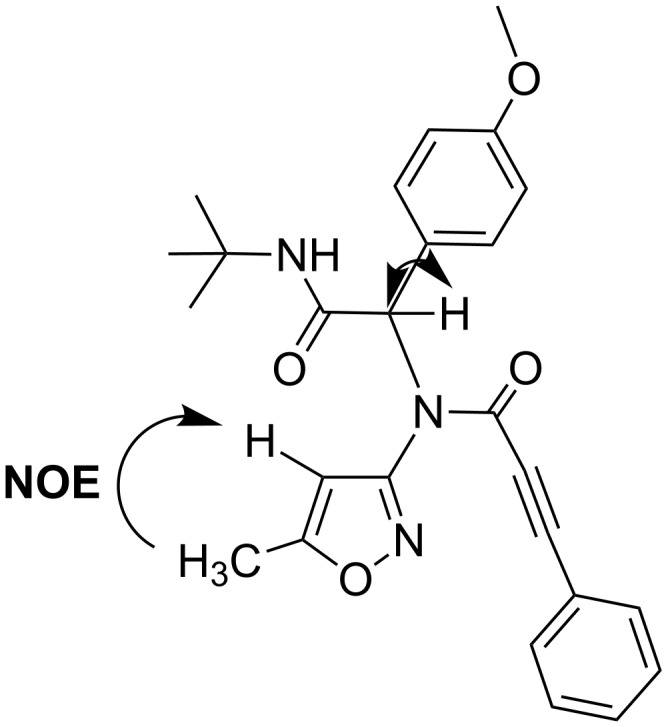
Selected data of NOE and HSQC experiments for compound **9d**.

In the case of compounds **9** the presence of NOE between the signals of the methyl group and the CH group in the isoxazole moiety allowed to distinguish closely located signals of two CH groups (Figure 4).

**Figure 5:**
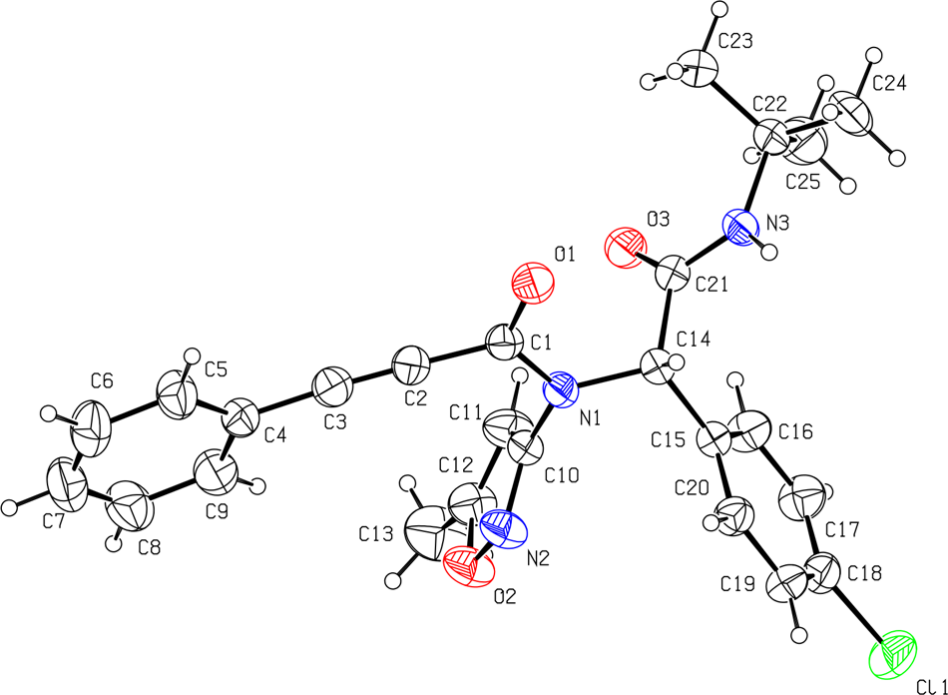
Molecular structure of *N*-(2-(*tert*-butylamino)-1-(4-chlorophenyl)-2-oxoethyl)-*N*-(5-methylisoxazol-3-yl)-3-phenylpropiolamide (**9e**) (X-ray diffraction data). Non-hydrogen atoms are presented as thermal ellipsoids with 50% probability.

Ultimately, the structure of compounds **9** was proven by an X-ray analysis of compound **9e** (Figure 5).

### Antibacterial activity

The antibacterial activity of compounds **4**, **6** and **9** (Table 7) was studied (see Experimental part in Supporting Information File 1 for details) against reference bacterial cultures: *Bacillus subtilis* (strain 1211), *Staphylococcus aureus* (strain 2231) (Gram-positive) and *Escherichia coli* (strain 1257), *Pseudomonas aeruginosa* (strain 1111) (Gram-negative).

**Table 7:** Antibacterial activity results.

Entry	Substance	MIC^a^/MBC^b^, mg/L	Strains of test cultures			
			*Escherichia* *coli*	*Pseudomonas aeruginosa*	*Staphylococcus* *aureus*	*Bacillus* *subtilis*

1	**4a**	MIC	125	–^c^	–	–
		MBC	–	–	–	–
2	**4b**	MIC	500	–	–	–
		MBC	–	–	–	–
3	**4c**	MIC	–	–	–	–
		MBC	–	–	–	–
4	**4d**	MIC	–	–	–	–
		MBC	–	–	–	–
5	**4e**	MIC	500	–	–	–
		MBC	–	–	–	–
6	**4f**	MIC	–	–	–	–*^d^
		MBC	–	–	–	–
7	**4g**	MIC	–	–	–	–
		MBC	–	–	–	–
8	**4i**	MIC	500	–	–	500
		MBC	–	–	–	–
9	**4j**	MIC	250	–	–	–*
		MBC	–	–	–	–
10	**4k**	MIC	–	–	–*	–
		MBC	–	–	–	–
11	**4n**	MIC	–	–	–	–*
		MBC	–	–	–	–
12	**4o**	MIC	–	–	–	–
		MBC	–	–	–	–
13	**4p**	MIC	500	–	–	–
		MBC	–	–	–	–
14	**4q**	MIC	–	–	–	–*
		MBC	–	–	–	–
15	**4r**	MIC	250	–	–	–
		MBC	–	–	–	–
16	**4s**	MIC	500	–	–	–*
		MBC	–	–	–	–
17	**4t**	MIC	–	–	–	–*
		MBC	–	–	–	–
18	**4u**	MIC	–	–	–	–
		MBC	–	–	–	–
19	**4v**	MIC	500	500	–	–
		MBC	–	–	–	–
20	**4w**	MIC	–	–	–	–
		MBC	–	–	–	–
21	**4x**	MIC	–	–	–	–
		MBC	–	–	–	–
22	**6a**	MIC	500	–	–	–*
		MBC	–	–	–	–
23	**6b**	MIC	–	–	–	–
		MBC	–	–	–	–
24	**6c**	MIC	500	–	–	–
		MBC	–	–	–	–
25	**6d**	MIC	–	–	–	500
		MBC	–	–	–	–
26	**6e**	MIC	–	500	–	–
		MBC	–	–	–	–
27	**6f**	MIC	500	–	–	–
		MBC	–	–	–	–
28	**6g**	MIC	125	–	–	–
		MBC	–	–	–	–
29	**6h**	MIC	500	–	500	–
		MBC	–	–	–	–
30	**9a**	MIC	–	–	–	–
		MBC	–	–	–	–
31	**9b**	MIC	–	–	–	–*
		MBC	–	–	–	–
32	**9c**	MIC	–	–	–	–*
		MBC	–	–	–	–
33	**9d**	MIC	–	–	–	–*
		MBC	–	–	–	–
34	**9e**	MIC	–	–	–	–
		MBC	–	–	–	–
35	**9f**	MIC	–	–	–	–*
		MBC	–	–	–	–
36	**Nitroxoline**	MIC	15.6	62.5	31.25	1.9
		MBC	15.6	62.5	31.25	1.9

^a^MIC – minimum inhibitory concentration; ^b^MBC – minimum bactericidal concentration; ^c^the substance in concentration ≤500 mg/L does not inhibit the culture growth; ^d^increase in biomass compared to control.

As it follows from the results obtained several of the substances studied inhibit the growth of test-microorganisms demonstrating a weak antimicrobial effect (Table 7). Generally, the compounds were found to be less active than nitroxoline (being the reference substance).

The antimicrobial effect of the heterocycles studied is different depending on each bacterical strain; however, some rules can be seen. Only a few substances inhibited the growth of Gram-negative bacteria (strains of *E. coli* and *P. aeruginosa*) in an effective way. Particularly, compounds **4a** and **6g** inhibited the growth of *E. coli* in concentration 125 mg/L. The bacteriostatic activity against *P. aeruginosa* of compounds **4v** and **6e** was fixed only in the highest concentration 500 mg/L. The Gram-positive bacterium *S. aureus* showed the resistance to almost all the compounds tested in the given concentration range. The strain of *B. subtilis* was found to be sensitive to compounds **4i** and **6d**, but the bacteriostatic activity was fixed only in the highest concentration 500 mg/L. The information about the influence of the compounds on bacteria is important from the point of view of choosing the further strategy for the investigations of their biological action. An absence or a low level of antibacterial activity of the heterocycles synthesized is a good prerequisite for carrying out the research on the other promising types of activity, e.g., anticancer, antidiabetic, etc., because in this case a negative influence on a microflora of the organism is decreased [110].

The other interesting feature of most of the compounds was the 30–50% increase in biomass of Gram-positive strains (mainly *B. subtilis*) compared to control. As it follows from a brief literature overview there are a lot of applications of metabolites (recombinant insulin [111], polyhydroxyalkanoates [112–113] etc*.* [114–119]) produced by the strains studied. Therefore, the found probiotic effect of the heterocycles has a very promising area for the further application while scaling up the production of biomass with the aim of shortening the time and saving resources [120–121]. Although this is a subject for a future detailed study the results of antibacterial activity allowed outlining the positive tendency.

## Conclusion

In summary, the behavior of 5-amino-*N*-aryl-1*H*-pyrazole-4-carboxamide and 3-amino-5-methylisoxazole as an amine component in isocyanide-based multicomponent reactions was studied. Particularly, 5-amino-*N*-aryl-1*H*-pyrazole-4-carboxamide reacted as 1,3-binucleophile with aromatic aldehydes and alkylisocyanides with the formation of 3-(alkylamino)-*N*,2-diaryl-1*H*-imidazo[1,2-*b*]pyrazole-7-carboxamides (Groebke–Blackburn–Bienaymé reaction). In contrast, 3-amino-5-methylisoxazole acted as a primary amine in Ugi four-component reaction with aromatic aldehydes, phenylpropiolic acid and *tert*-butylisocyanide giving *N*-(1-arylethyl-2-(*tert*-butylamino)-2-oxo)-*N*-(5-methylisoxazol-3-yl)-3-phenylpropiolamides.

The optimal reaction conditions were different depending on the substituent in the carbonyl component and the structure of the isocyanide. Thus, GBB-3CR involving *tert*-butylisocyanide in the case of aldehydes with electron-donating substituents was carried out in an EtOH/H_2_O mixture with TFA (10 mol %) at rt for 24 h and in DMF/HClO_4_ (10 mol %) at rt for 48 h in case of electron-withdrawing groups. When replacing *tert*-butylisocyanide with ethyl 2-isocyanoacetate the similar imidazo[1,2-*b*]pyrazole-7-carboxamides were isolated from the treatment in TFE/HClO_4_ (10 mol %) at rt for 24 h. Ugi-4CR involving *tert*-butylisocyanide proceeded under standard conditions in MeOH.

The broad antibacterial activity of the obtained compounds was studied as well. Several of the substances inhibited the growth of test microorganisms demonstrating a weak antimicrobial effect. For most of the stuctures a 30–50% increase in biomass of Gram-positive strains (mainly *B. subtilis*) compared to control was observed. After a detailed study this effect may be used to stimulate the growth of producers of biologically active compounds.

## Supporting Information

File 1 Experimental and analytical data.

File 2 NMR spectra.
