# Supplementary material for: New tricks of well-known aminoazoles in isocyanide-based multicomponent reactions and antibacterial activity of the compounds synthesized
Source: Beilstein J Org Chem. 2017 May 31;13:1050–63. doi: 10.3762/bjoc.13.104 (PMC5480327; doi:10.3762/bjoc.13.104)
Supplement: File 1 — Experimental and analytical data. [file Beilstein_J_Org_Chem-13-1050-s001.pdf]

Supporting Information File 1

for

New tricks of well-known aminoazoles in isocyanide-based multicomponent reactions and antibacterial activity of the compounds synthesized

Maryna V. Murlykina[1,2], Maryna N. Kornet[3], Sergey M. Desenko[1,4], Svetlana V. Shishkina[1,4], Oleg V. Shishkin[1], Aleksander A. Brazhko[3], Vladimir I. Musatov[1], Erik V. Van der Eycken[2] and Valentin A. Chebanov[*,1,2,4]

Address: [a]Division of Chemistry of Functional Materials, State Scientific Institution "Institute for Single Crystals" of National Academy of Sciences of Ukraine, Nauky Ave., 60, 61001, Kharkiv, Ukraine, [b]Laboratory for Organic & Microwave-Assisted Chemistry (LOMAC), KU Leuven, Celestijnenlaan 200F, B-3001, Leuven, Belgium, [c]Laboratory of Biotechnology of Physiologically Active Substances, Zaporizhzhya National University, Zhukovsky str., 66, Zaporizhzhya, Ukraine, 69600 and [d]Faculty of Chemistry, V. N. Karazin Kharkiv National University, Svobody sq., 4, 61077, Kharkiv, Ukraine

Email: Valentin A. Chebanov - chebanov@isc.kharkov.com

*Corresponding author

Experimental and analytical data

Experimental section

1. General

The starting 5-amino-*N*-aryl-1*H*-pyrazole-4-carboxamides were synthesized according to the known literature procedure [1]. Alkylisocyanides, phenylpropiolic acid and substituted aldehydes were commercially available.

Melting points of all the compounds synthesized were determined with a Kofler melting point apparatus and are uncorrected. The NMR spectra were recorded in DMSO-d_6 at 200 MHz with a Varian Mercury VX-200 spectrometer, at 300 MHz (75 MHz for ^{13}C) with a Bruker Avance 300 spectrometer, at 400 MHz (100 MHz for ^{13}C) with a Varian MR-400 and at 600 MHz (150 MHz for ^{13}C) with a Bruker Avance 600 spectrometer. The high-resolution mass spectra were recorded on KRATOS MS50TC system (EI) and on Bruker APEX II FT/ICR instrument (ESI).

Microwave experiments were carried out using an Emrys™ Creator EXP synthesizer possessing a single-mode microwave cavity producing controlled irradiation at 2.45 GHz in sealed microwave process vials utilizing the high absorbance level. The reaction time reflects the irradiation times at the set reaction temperature.

2. Chemistry

General procedure for the synthesis of *N*,2-diaryl-3-(*tert*-butylamino)-1*H*-imidazo[1,2-*b*]pyrazole-7-carboxamides 4a–m (Method A).

The appropriate 5-amino-*N*-aryl-1*H*-pyrazole-4-carboxamide **2a–d** (1 mmol) and aromatic aldehyde **1a–e** (1 mmol) were dissolved in 8 mL H_2O/EtOH (50:50 vol. %) and were stirred for 5 min. Then 10 mol % of TFA (1 M water/ethanol solution) was added and the mixture was stirred for additional 15 min. After that 1 mmol *tert*-butylisocyanide

(**3a**) was introduced and the reaction was stirred for 24 h until completion. The formed precipitate was filtered off and dried under vacuum.

When purification is needed, the following procedure can be used. The target compound **4** is heated (at ca. 80 °C, 60 min) in H_2O/EtOH (50:50, vol. %) and then is filtered off from the hot solution and dried under vacuum.

Characterization data for compounds 4a–m.

*3-(tert-Butylamino)-N-(4-fluorophenyl)-2-phenyl-1H-imidazo[1,2-b]pyrazole-7-carboxamide (**4a**).* Colorless solid, mp 182-183 °C. ^{1}H NMR (300 MHz, DMSO-d_6) δ = 11.87 (s, 1H, NH), 9.53 (s, 1H, NH), 8.18 (s, 1H, CH), 7.10-8.01 (m, 9H, ArH), 4.32 (s, 1H, NH), 1.04 (s, 9H, $\underline{(CH_3)_3}C$) ppm; ^{13}C NMR (75 MHz, DMSO-d_6): δ = 160.7, 157.7 (d, *J* = 238.8 Hz), 141.4, 137.8, 136.2, 130.5, 128.1, 127.1, 126.9, 124.5, 121.9, 121.3 (d, *J* = 7.6 Hz), 115.0 (d, *J* = 22.1 Hz), 94.2, 54.7, 30.1 ppm. HRMS (EI) calcd for $C_{22}H_{22}FN_5O$ $[M]^+$ 391.18084, found 391.18181.

*3-(tert-Butylamino)-N-(4-fluorophenyl)-2-(2-methoxyphenyl)-1H-imidazo[1,2-b]pyrazole-7-carboxamide (**4b**).* Colorless solid, mp 142-143 °C. ^{1}H NMR (300 MHz, DMSO-d_6) δ = 11.73 (s, 1H, NH), 9.49 (s, 1H, NH), 8.18 (s, 1H, CH), 7.03-8.18 (m, 8H, ArH), 3.84 (s, 3H, $\underline{CH_3}O$), 0.93 (s, 9H, $\underline{(CH_3)_3}C$) ppm; ^{13}C NMR (75 MHz, DMSO-d_6): δ = 160.7, 157.6 (d, *J* = 238.9 Hz), 156.2, 141.0, 138.0, 136.2, 131.3, 129.8, 122.5, 121.3 (d, *J* = 7.6 Hz), 120.8, 120.5, 118.9, 115.0 (d, *J* = 22.0 Hz), 111.6, 93.9, 55.7, 54.5, 29.6 ppm. HRMS (EI) calcd for $C_{23}H_{24}FN_5O_2$ $[M]^+$ 421.19140, found 421.19470.

*3-(tert-Butylamino)-N-(4-fluorophenyl)-2-(3-methoxyphenyl)-1H-imidazo[1,2-b]pyrazole-7-carboxamide (**4c**).* Colorless solid, mp 185-186 °C. ^{1}H NMR (200 MHz, DMSO-d_6) δ = 11.87 (s, 1H, NH), 9.54 (s, 1H, NH), 8.19 (s, 1H, CH), 6.79-7.79 (m, 8H, ArH), 4.33 (s, 1H, NH), 3.80 (s, 3H, $\underline{CH_3}O$), 1.07 (s, 9H, $\underline{(CH_3)_3}C$) ppm; ^{13}C NMR (100

MHz, DMSO-d_6): δ = 160.7, 159.1, 157.7 (d, *J* = 239.1 Hz), 141.5, 137.8, 136.1, 131.7, 129.1, 124.2, 122.1, 121.4 (d, *J* = 7.7 Hz), 119.1, 115.0 (d, *J* = 22.1 Hz), 113.3, 111.9, 94.2, 55.2, 54.7, 30.1 ppm. HRMS (ESI/FT-ICR) calcd for $C_{23}H_{24}FN_5O_2$ $[M+H]^+$ 422.1992, found 422.1972.

*3-(tert-Butylamino)-N-(4-fluorophenyl)-2-(4-methoxyphenyl)-1H-imidazo[1,2-b]pyrazole-7-carboxamide (**4d**).* Colorless solid, mp 167-168 °C. ^1H NMR (300 MHz, DMSO-d_6) δ = 11.82 (s, 1H, NH), 9.52 (s, 1H, NH), 8.18 (s, 1H, CH), 6.95-7.94 (m, 8H, ArH), 3.77 (s, 3H, $\underline{CH_3}O$), 1.04 (s, 9H, $\underline{(CH_3)_3}C$) ppm; ^{13}C NMR (75 MHz, DMSO-d_6): δ = 160.7, 159.2, 157.7 (d, *J* = 238.9 Hz), 141.0, 137.5, 136.2, 128.4, 124.7, 122.8, 121.3 (d, *J* = 7.7 Hz), 120.9, 115.03 (d, *J* = 22.0 Hz), 113.5, 94.3, 55.1, 54.7, 30.0 ppm. HRMS (EI) calcd for $C_{23}H_{24}FN_5O_2$ $[M]^+$ 421.19140, found 421.19360.

*3-(tert-Butylamino)-2-(4-chlorophenyl)-N-(4-fluorophenyl)-1H-imidazo[1,2-b]pyrazole-7-carboxamide (**4e**).* Colorless solid, mp 161-162 °C. ^1H NMR (300 MHz, DMSO-d_6) δ = 11.97 (s, 1H, NH), 9.56 (s, 1H, NH), 8.20 (s, 1H, CH), 7.10-8.07 (m, 8H, ArH), 1.04 (s, 9H, $\underline{(CH_3)_3}C$) ppm; ^{13}C NMR (75 MHz, DMSO-d_6): δ = 160.7, 157.7 (d, *J* = 238.8 Hz) 141.5, 138.1, 136.1, 131.6, 129.4, 128.6, 128.1, 123.5, 122.3, 121.3 (d, *J* = 7.7 Hz), 115.0 (d, *J* = 22.1 Hz), 94.3, 54.8, 30.1 ppm. HRMS (EI) calcd for $C_{22}H_{21}FClN_5O$ $[M]^+$ 425.14187, found 425.14189.

*3-(tert-Butylamino)-N-(3-fluorophenyl)-2-(2-methoxyphenyl)-1H-imidazo[1,2-b]pyrazole-7-carboxamide (**4f**).* Colorless solid, mp 190-191 °C. ^1H NMR (400 MHz, DMSO-d_6) δ = 11.72 (s, 1H, NH), 9.62 (s, 1H, NH), 8.19 (s, 1H, CH), 6.80-7.74 (m, 8H, ArH), 3.85 (s, 3H, $\underline{CH_3}O$), 0.93 (s, 9H, $\underline{(CH_3)_3}C$) ppm; ^{13}C NMR (100 MHz, DMSO-d_6): ^{13}C NMR (100 MHz, dmso) δ = 162.2 (d, *J* = 240.2 Hz), 161.0, 156. 2, 141.8 (d, *J* = 11.4 Hz), 141.1, 138.2, 131.3, 130.0 (d, *J* = 9.9 Hz), 129.7, 122.9, 120.8, 120.5, 119.0, 115.07,

111.7, 108.7 (d, *J* = 21.2 Hz), 106.1 (d, *J* = 26.4 Hz), 93.8, 55.7, 54.3, 29.7 ppm. HRMS (ESI/FT-ICR) calcd for $C_{23}H_{24}FN_5O_2$ $[M+H]^+$ 422.1992, found 422.1979.

*3-(tert-Butylamino)-N-(3-fluorophenyl)-2-(4-methoxyphenyl)-1H-imidazo[1,2-b]pyrazole-7-carboxamide (**4g**).* Colorless solid, mp 189-190 °C. ^{1}H NMR (200 MHz, DMSO-d_6) δ = 11.85 (s, 1H, NH), 9.67 (s, 1H, NH), 8.19 (s, 1H, CH), 6.75-7.94 (m, 8H, ArH), 4.25 (s, 1H, NH), 3.77 s, 3H, $\underline{CH_3}O$), 1.03 (s, 9H, $\underline{(CH_3)_3}C$) ppm; ^{13}C NMR (100 MHz, DMSO-d_6): δ = 162.1 (d, *J* = 239.8 Hz), 160.9, 158.4, 141.8 (d, *J* = 11.3 Hz), 141.1, 137.7, 130.0 (d, *J* = 9.6 Hz), 128.4, 124.6, 122.8, 121.1, 115.1, 113.5, 108.7 (d, *J* = 21.1 Hz), 106.1 (d, *J* = 26.3 Hz), 94.2, 55.1, 54.5, 30.1 ppm. HRMS (ESI/FT-ICR) calcd for $C_{23}H_{24}FN_5O_2$ $[M+H]^+$ 422.1992, found 422.1974.

*3-(tert-Butylamino)-2-(4-chlorophenyl)-N-(3-fluorophenyl)-1H-imidazo[1,2-b]pyrazole-7-carboxamide (**4h**).* Colorless solid, mp 180-181 °C. ^{1}H NMR (400 MHz, DMSO-d_6) δ = 12.02 (s, 1H, NH), 9.70 (s, 1H, NH), 8.23 (s, 1H, CH), 6.75-8.06 (m, 8H, ArH), 4.38 (s, 1H, NH), 1.05 (s, 9H, $\underline{(CH_3)_3}C$) ppm; ^{13}C NMR (100 MHz, DMSO-d_6): δ = 162.2 (d, *J* = 240.3 Hz), 160.9, 141.7 (d, *J* = 11.4 Hz), 141.5, 138.3, 131. 7, 130.1 (d, *J* = 9.4 Hz), 129.4, 128.6, 128.2, 123.6, 122.4, 115.1, 108.8 (d, *J* = 21.6 Hz), 106.1 (d, *J* = 26.0 Hz), 94.3, 54.8, 30.1 ppm. HRMS (ESI/FT-ICR) calcd for $C_{22}H_{21}ClFN_5O$ $[M+H]^+$ 426.1497, found 426.1468.

*3-(tert-Butylamino)-N-(2-ethylphenyl)-2-(2-methoxyphenyl)-1H-imidazo[1,2-b]pyrazole-7-carboxamide (**4i**).* Colorless solid, mp 105-106 °C. ^{1}H NMR (400 MHz, DMSO-d_6) δ = 11.52 (s, 1H, NH), 8.95 (s, 1H, NH), 8.08 (s, 1H, CH), 7.02-7.53 (m, 8H, ArH), 3.83 (s, 3H, $\underline{CH_3}O$), 2.62 (q, *J* = 7.7 Hz, 2H, $\underline{CH_2}CH_3$), 1.09 (d, *J* = 7.7 Hz, 1H, $CH_2\underline{CH_3}$), 0.92 (s, 9H, $\underline{(CH_3)_3}C$) ppm; ^{13}C NMR (100 MHz, DMSO-d_6): δ = 161.1, 156.1, 141.6, 139.5, 137.7, 136.1, 131.1, 128.3, 127.4, 125.9, 125.8, 123. 9, 122.8, 120.6, 120.5,

119.1, 111.7, 93.6, 55.7, 54.3, 29.8, 24.0, 14.1 ppm. HRMS (ESI/FT-ICR) calcd for $C_{25}H_{29}N_5O_2$ $[M+H]^+$ 432.2400, found 432.2356.

*3-(tert-Butylamino)-N-(2-ethylphenyl)-2-(4-methoxyphenyl)-1H-imidazo[1,2-b]pyrazole-7-carboxamide (**4j**).* Colorless solid, mp 98-99 °C. ^{1}H NMR (400 MHz, DMSO-d$_6$) δ = 11.66 (s, 1H, NH), 9.00 (s, 1H, NH), 8.11 (s, 1H, CH), 6.96-7.98 (m, 8H, ArH), 4.23 (s, 1H, NH), 3.79 (s, 3H, $\underline{CH_3}O$), 2.67 (q, *J* = 7.5 Hz, 2H, $\underline{CH_2}CH_3$), 1.14 (t, *J* = 7.6 Hz, 3H, $CH_2\underline{CH_3}$), 1.07 (s, 9H, $\underline{(CH_3)_3}C$) ppm; ^{13}C NMR (100 MHz, DMSO-d$_6$): δ = 161.1, 158.4, 141.6, 139.5, 137.1, 136.0, 128.4, 128.2, 127.4, 125.9, 125.8, 124.4, 122.9, 121.0, 113.6, 94.0, 55.1, 54.6, 30.1, 24.0, 14.1 ppm. HRMS (ESI/FT-ICR) calcd for $C_{25}H_{29}N_5O_2$ $[M-H]^+$ 430.2243, found 430.2212.

*3-(tert-Butylamino)-2-(4-chlorophenyl)-N-(2-ethylphenyl)-1H-imidazo[1,2-b]pyrazole-7-carboxamide (**4k**).* Colorless solid, mp 125-126 °C. ^{1}H NMR (200 MHz, DMSO-d$_6$) δ = 11.84 (s, 1H, NH), 9.04 (s, 1H, NH), 8.15 (s, 1H, CH), 7.13-8.04 (m, 8H, ArH), 4.39 (s, 1H, NH), 2.65 (q, *J* = 7.7 Hz, 2H, $\underline{CH_2}CH_3$), 1.12 (t, *J* = 7.6 Hz, 4H, $CH_2\underline{CH_3}$), 1.06 (s, 9H, $\underline{(CH_3)_3}C$) ppm; ^{13}C NMR (100 MHz, DMSO-d$_6$): δ = 161.0, 141.9, 139.5, 137.8, 136.0, 131.6, 129.5, 128.5, 128.4, 128.2, 127.3, 125.9, 125.8, 123.3, 122.3, 94.1, 54.8, 30.1, 24.0, 14.2 ppm. HRMS (ESI/FT-ICR) calcd for $C_{24}H_{26}ClN_5O$ $[M+H]^+$ 436.1904, found 436.1870.

*3-(tert-Butylamino)-N-(4-ethylphenyl)-2-(2-methoxyphenyl)-1H-imidazo[1,2-b]pyrazole-7-carboxamide (**4l**).* Colorless solid, mp 175-177 °C. ^{1}H NMR (200 MHz, DMSO-d$_6$) δ = 11.67 (s, 1H, NH), 9.36 (s, 1H, NH), 8.17 (s, 1H, CH), 7.08-7.57 (m, 8H, ArH), 3.85 (s, 3H, $\underline{CH_3}O$), 2.48 (q, 2H, $\underline{CH_2}CH_3$), 1.15 (t, 4H, $CH_2\underline{CH_3}$), 0.92 (s, 9H, $\underline{(CH_3)_3}C$) ppm; ^{13}C NMR (100 MHz, DMSO-d$_6$): δ = 160.7, 156.2, 141.2, 138.1, 137.8, 137.5, 131.2, 129.6, 127.7, 122.8, 120.6, 120.5, 119.8, 119.1, 111.6, 94.1, 55.7, 54.3,

29.7, 27.6, 15.8 ppm. HRMS (ESI/FT-ICR) calcd for $C_{25}H_{29}N_5O_2$ $[M+H]^+$ 432.2400, found 432.2395.

*3-(tert-Butylamino)-2-(4-chlorophenyl)-N-(4-ethylphenyl)-1H-imidazo[1,2-b]pyrazole-7-carboxamide (**4m**).* Colorless solid, mp 166-167 °C. ^1H NMR (200 MHz, DMSO-d$_6$) δ = 11.92 (s, 1H, NH), 9.42 (s, 1H, NH), 8.21 (s, 1H, CH), 7.13-8.06 (m, 8H, ArH), 4.35 (s, 1H, NH), 2.48 (q, 2H, $\underline{CH_2}CH_3$), 1.15 (t, 4H, $CH_2\underline{CH_3}$), 1.05 (s, 9H, $\underline{(CH_3)_3}C$) ppm; ^{13}C NMR (100 MHz, DMSO-d$_6$): δ = 160.6, 141.4, 138.1, 137.82, 137.4, 131.5, 129.4, 128.5, 128.1, 127. 7, 123.4, 122.2, 119.7, 94.5, 54.7, 30.1, 27.6, 15.7 ppm. HRMS (ESI/FT-ICR) calcd for $C_{24}H_{26}ClN_5O$ $[M+H]^+$ 436.1904, found 436.1877.

General procedure for the synthesis of *N*,2-diaryl-3-(*tert*-butylamino)-1*H*-imidazo[1,2-*b*]pyrazole-7-carboxamides 4n–v (Method B).

The appropriate 5-amino-*N*-aryl-1*H*-pyrazole-4-carboxamide **2a–c** (1 mmol) and aromatic aldehyde **1f–h** (1 mmol) were dissolved in DMF (0,5 mL) and stirred for 5 min. Then $HClO_4$ (1 M water solution, 10 mol %) was added and the mixture was stirred for additional 15 min. After that 1 mmol of *tert*-butylisocyanide (**3a**) was introduced and the reaction was stirred for 48 h till completion. The solution was put on ice (ca. 30 mL) and ultrasonicated with an ultrasonic horn (22 kHz) for 30 min and then put to a freezer for 1 h. The formed precipitate was filtered off and dried under vacuum.

When purification is needed, the following procedure can be used: compound **4** is heated (at ca. 80 °C, 60 min) in H_2O/EtOH (50:50, vol. %) mixture and then is filtered off from the hot solution and dried under vacuum.

Characterization data for compounds 4n–v.

*Methyl 4-(3-(tert-butylamino)-7-(4-fluorophenylcarbamoyl)-1H-imidazo[1,2-b]pyrazol-2-yl)benzoate (**4n**).* Colorless solid, mp 197-198 °C. ^1H NMR (400 MHz,

DMSO-d_6) δ = 12.03 (s, 1H, NH), 9.56 (s, 1H, NH), 8.22 (s, 1H, CH), 7.12-8.25 (m, 8H, ArH), 4.46 (s, 1H, NH), 3.82 (s, 3H, $CO_2\underline{CH_3}O$), 1.05 (s, 9H, $\underline{(CH_3)_3}C$) ppm; ^{13}C NMR (100 MHz, DMSO-d_6): δ = 166.4, 161.1, 158.1 (d, *J* = 239.0 Hz), 142.4, 138.9, 136.6, 135.7, 129.4, 128.1, 127.1, 123.9, 123.8, 121.8 (d, *J* = 7.6 Hz), 115.5 (d, *J* = 22.0 Hz), 94.7, 55.5, 52.5, 30.5 ppm. HRMS (ESI/FT-ICR) calcd for $C_{24}H_{24}FN_5O_3$ $[M+H]^+$ 450.1941, found 450.1920.

*3-(tert-Butylamino)-N-(4-fluorophenyl)-2-(4-nitrophenyl)-1H-imidazo[1,2-b]pyrazole-7-carboxamide (**4o**)*. Colorless solid, mp 200-201 °C. ^1H NMR (400 MHz, DMSO-d_6) δ = 12.18 (s, 1H, NH), 9.63 (s, 1H, NH), 8.36 (s, 1H, CH), 7.14-8.41 (m, 8H, ArH), 4.61 (s, 1H, NH), 1.09 (s, 9H, $\underline{(CH_3)_3}C$) ppm; ^{13}C NMR (100 MHz, DMSO-d_6): δ = 160.7, 157.8 (d, *J* = 239.2 Hz), 145.5, 142.3, 139.0, 137.3, 136.1, 127.2, 124.5, 123.5, 122.6, 121.4 (d, *J* = 7.6 Hz), 115.1 (d, *J* = 22.1 Hz), 94.4, 55.3, 30.1 ppm. HRMS (ESI/FT-ICR) calcd for $C_{22}H_{21}FN_6O_3$ $[M+H]^+$ 437.1737, found 437.1702.

*3-(tert-Butylamino)-2-(4-cyanophenyl)-N-(4-fluorophenyl)-1H-imidazo[1,2-b]pyrazole-7-carboxamide (**4p**)*. Colorless solid, mp 195-197 °C. ^1H NMR (400 MHz, DMSO-d_6) δ = 12.10 (s, 1H, NH), 9.61 (s, 1H, NH), 8.28 (s, 1H, CH), 7.14-8.27 (m, 8H, ArH), 4.55 (s, 1H, CH), 1.07 (s, 9H, $\underline{(CH_3)_3}C$) ppm; ^{13}C NMR (100 MHz, DMSO-d_6): δ = 160.6, 157.7 (d, *J* = 238.5 Hz), 142.1, 138.8, 136.1, 135.2, 132.0, 127.1, 123.9, 122.8, 121.3 (d, *J* = 7.7 Hz), 118.9, 115.1 (d, *J* = 22.2 Hz), 109.0, 94.3, 55.1, 30.1 ppm. HRMS (ESI/FT-ICR) calcd for $C_{23}H_{21}FN_6O$ $[M+H]^+$ 417.1839, found 417.1805.

*Methyl 4-(3-(tert-butylamino)-7-(3-fluorophenylcarbamoyl)-1H-imidazo[1,2-b]pyrazol-2-yl)benzoate (**4q**)*. Colorless solid, mp 139-140 °C. ^1H NMR (400 MHz, DMSO-d_6) δ = 12.12 (s, 1H, NH), 9.72 (s, 1H, NH), 8.27 (s, 1H, CH), 6.82-8.21 (m, 8H, ArH), 4.49 (s, 1H, NH), 3.84 (s, 3H, $CO_2\underline{CH_3}O$), 1.06 (s, 9H, $\underline{(CH_3)_3}C$) ppm; ^{13}C NMR

(100 MHz, DMSO-d_6): δ = 166.0, 162.2 (d, *J* = 240.5 Hz), 160.9, 141.9, 141.7 (d, *J* = 11.0 Hz), 138.6, 135.2, 130.1 (d, *J* = 9.3 Hz), 129.8, 129.0, 127.7, 126.7, 123.5, 115.2, 108.9 (d, *J* = 21.2 Hz), 106.2 (d, *J* = 26.1 Hz), 94.3, 55.1, 52.1, 30.1 ppm. HRMS (ESI/FT-ICR) calcd for $C_{24}H_{24}FN_5O_3$ $[M+H]^+$ 450.1941, found 450.1914.

*3-(tert-Butylamino)-N-(3-fluorophenyl)-2-(4-nitrophenyl)-1H-imidazo[1,2-b]pyrazole-7-carboxamide (**4r**)*. Colorless solid, mp 218-219 °C. ^1H NMR (400 MHz, DMSO-d_6) δ = 12.25 (s, 1H, NH), 9.77 (s, 1H, NH), 8.37 (s, 1H, CH), 6.82-8.36 (m, 8H, ArH), 4.63 (s, 1H, NH), 1.09 (s, 9H, $\underline{(CH_3)_3}C$) ppm; ^{13}C NMR (100 MHz, DMSO-d_6): δ = 162.2 (d, *J* = 240.3 Hz), 160.8, 145.5, 142.3, 141.6 (d, *J* = 11.3 Hz), 139.1, 137.2, 130.1 (d, *J* = 9.6 Hz), 127.2, 124.5, 123.4, 122.6, 115.1, 108.9 (d, *J* = 21.3 Hz), 106.2 (d, *J* = 26.4 Hz), 94.8, 55.3, 30.1 ppm. HRMS (ESI/FT-ICR) calcd for $C_{22}H_{21}FN_6O_3$ $[M+H]^+$ 437.1737, found 437.1720.

*3-(tert-Butylamino)-2-(4-cyanophenyl)-N-(3-fluorophenyl)-1H-imidazo[1,2-b]pyrazole-7-carboxamide (**4s**)*. Colorless solid, mp 234-235 °C. ^1H NMR (400 MHz, DMSO-d_6) δ = 12.16 (s, 1H, NH), 9.74 (s, 1H, NH), 8.28 (s, 1H, CH), 6.77-8.27 (m, 8H, ArH), 4.57 (s, 1H, NH), 1.07 (s, 9H, $\underline{(CH_3)_3}C$) ppm; ^{13}C NMR (100 MHz, DMSO-d_6): δ = 162.15 (d, *J* = 240.4 Hz), 160.8, 142.1, 141.6 (d, *J* = 11.5 Hz), 138.8, 135.1, 132.0, 130.1 (d, *J* = 9.4 Hz), 127.1, 123.9, 122.9, 118.9, 115.1, 109.0, 108.8 (d, *J* = 21.1 Hz), 106.1 (d, *J* = 26.3 Hz), 94.3, 55.1, 30.1 ppm. HRMS (ESI/FT-ICR) calcd for $C_{23}H_{21}FN_6O$ $[M+H]^+$ 417.1839, found 417.1815.

*Methyl 4-(3-(tert-butylamino)-7-(2-ethylphenylcarbamoyl)-1H-imidazo[1,2-b]pyrazol-2-yl)benzoate (**4t**)*. Colorless solid, mp 103-104 °C. ^1H NMR (400 MHz, DMSO-d_6) δ = 11.93 (s, 1H, NH), 9.05 (s, 1H, NH), 8.21 (s, 1H, CH), 7.13-8.18 (m, 8H, ArH), 3.84 (s, 3H, $CO_2\underline{CH_3}O$), 2.66 (q, *J* = 7.5 Hz, 2H, $\underline{CH_2}CH_3$), 1.13 (t, *J* = 7.7 Hz, 3H,

$CH_2\underline{CH_3}$), 1.08 (s, 9H, $\underline{(CH_3)_3}C$) ppm; ^{13}C NMR (100 MHz, DMSO-d_6): δ = 165.9, 160.9, 142.4, 139.5, 138.1, 136.0, 135.3, 129.0, 128.4, 127.6, 127.4, 126.5, 125.9, 123.4, 123.2, 94.1, 55.1, 52.1, 30.1, 24.0, 14.1 ppm. HRMS (ESI/FT-ICR) calcd for $C_{26}H_{29}N_5O_3$ $[M+H]^+$ 460.2349, found 460.2331.

*3-(tert-Butylamino)-N-(2-ethylphenyl)-2-(4-nitrophenyl)-1H-imidazo[1,2-b]pyrazole-7-carboxamide (**4u**).* Colorless solid, mp 99-100 °C. ^1H NMR (400 MHz, DMSO-d_6) δ = 12.08 (s, 1H, NH), 9.12 (s, 1H, NH), 8.35 (s, 1H, CH), 7.24-8.45 (m, 8H, ArH), 4.66 (s, 1H, NH), 2.65 (q, 2H, $\underline{CH_2}CH_3$), 1.10 (m, 12H, $CH_2\underline{CH_3 + (CH_3)_3}C$) ppm; ^{13}C NMR (100 MHz, DMSO-d_6): δ = 160.9, 145.5, 142.7, 139.5, 138.7, 137.3, 135.9, 128.7, 127.3, 127.0, 125.8, 124.5, 124.3, 123.5, 122.4, 94.2, 55.3, 30.1, 24.0, 14.1 ppm. HRMS (ESI/FT-ICR) calcd for $C_{24}H_{26}N_6O_3$ $[M-H]^+$ 445.1988, found 445.1960.

*3-(tert-Butylamino)-2-(4-cyanophenyl)-N-(2-ethylphenyl)-1H-imidazo[1,2-b]pyrazole-7-carboxamide (**4v**).* Colorless solid, mp 129-130 °C. ^1H NMR (400 MHz, DMSO-d_6) δ 11.98 (s, 1H, NH), 9.09 (s, 1H, NH), 8.18 (s, 1H, CH), 7.23-8.25 (m, 8H, ArH), 4.56 (s, 1H, NH), 2.65 (q, 2H, $\underline{CH_2}CH_3$), 1.08-1.14 (m, 12H, $CH_2\underline{CH_3}$ + $(\underline{CH_3})_3C$) ppm; ^{13}C NMR (100 MHz, DMSO-d_6): δ = 161.0, 142.5, 139.5, 138.4, 136.0, 135.2, 132.1, 128.4, 127.3, 126.9, 125.8, 125.8, 123.8, 122.7, 118.9, 109.0, 94.2, 55.1, 30.1, 24.0, 14.2 ppm. HRMS (ESI/FT-ICR) calcd for $C_{23}H_{24}FN_5O_2$ $[M-H]^+$ 425.2090, found 425.2062.

General procedure for the synthesis of *N*-aryl-2-benzyl-3-(*tert*-butylamino)-1*H*-imidazo[1,2-*b*]pyrazole-7-carboxamides 4w,x.

The appropriate 5-amino-*N*-aryl-1*H*-pyrazole-4-carboxamide **2a,b** (1 mmol) and 1 mmol of phenylpuryvic acid (**1'**) were dissolved in H_2O/EtOH (50:50, vol. %, 6 mL) mixture and stirred for 5 min. Then TFA (1 M water/ethanol solution, 10 mol %) was

added and the mixture was stirred for additional 15 min. After that *tert*-butylisocyanide (**3a**) (1 mmol) was introduced and the reaction was stirred for 48 h till completion. The formed precipitate was filtered off and dried under vacuum.

When purification is needed, the following procedure can be used: compound **4** is heated (at ca. 80 °C, 60 min) in H_2O/EtOH (50:50, vol. %) mixture and then is filtered off from the hot solution and dried under vacuum.

Characterization data for compounds 4w,x.

*2-Benzyl-3-(tert-butylamino)-N-(4-fluorophenyl)-1H-imidazo[1,2-b]pyrazole-7-carboxamide (**4w**).* Colorless solid, mp 174-175 °C. ^{1}H NMR (400 MHz, DMSO-d_6) δ = 11.62 (s, 1H, NH), 9.62 (s, 1H, NH), 8.14 (s, 1H, CH), 6.79-7.72 (m, 8H, ArH), 4.11 (s, 1H, NH), 3.93 (s, 1H, CH_2), 1.12 (s, 9H, $\underline{(CH_3)_3}$C) ppm; ^{13}C NMR (100 MHz, DMSO-d_6): δ = 162.1 (d, *J* = 240.1 Hz), 161.1, 141.8 (d, *J* = 11.3 Hz), 140.2 (d, *J* = 7.7 Hz), 139.4, 138.0, 130.0 (d, *J* = 9.2 Hz), 128.4, 128.1, 126.1, 124.0, 122.3, 114.9, 108.6 (d, *J* = 20.9 Hz), 105.8, 93.9, 53.6, 30.1, 29.9 ppm. HRMS (ESI/FT-ICR) calcd for $C_{23}H_{24}FN_5O$ $[M+H]^+$ 406.2043, found 406.2028.

*2-Benzyl-3-(tert-butylamino)-N-(3-fluorophenyl)-1H-imidazo[1,2-b]pyrazole-7-carboxamide (**4x**).* Colorless solid, mp 186-187 °C. ^{1}H NMR (400 MHz, DMSO-d_6) δ = 11.55 (s, 1H, NH), 9.48 (s, 1H, NH), 8.11 (s, 1H, CH), 7.06-7.69 (m, 8H, ArH), 4.10 (s, 1H, NH), 3.93 (s, 1H, CH_2), 1.12 (s, 9H, $\underline{(CH_3)_3}$C) ppm; ^{13}C NMR (100 MHz, DMSO-d_6): δ = 160.9, 157.6 (d, *J* = 238.7 Hz), 140.3, 140.1, 139.4, 137.9, 136.2, 128.38, 128.2, 126.1, 123.9, 122.2, 121.1 (d, *J* = 7.8 Hz), 115.0 (d, *J* = 22.0 Hz), 93.9, 53.6, 30.1, 29.9 ppm. HRMS (ESI/FT-ICR) calcd for $C_{23}H_{24}FN_5O$ $[M+H]^+$ 406.2043, found 406.2037.

General procedure for the synthesis of ethyl 2-(2-aryl-7-(arylcarbamoyl)-1*H*-imidazo[1,2-*b*]pyrazol-3-ylamino)acetates 6a–h.

The appropriate 5-amino-*N*-aryl-1*H*-pyrazole-4-carboxamide **2a,b** (1 mmol) and aromatic aldehyde **1d,e**,**f**,**g** (1 mmol) were dissolved in TFE (6 mL) and stirred for 5 min. Then, $HClO_4$ (1 M water solution, 10 mol %) was added and the mixture was stirred for additional 15 min. After that 1 mmol of ethyl 2-isocyanoacetate (**3b**) was introduced and the reaction was stirred for 24 h till completion. The formed precipitate was filtered off and dried under vacuum.

When purification is needed, the following procedure can be used: compound **6** is heated (at *ca*. 80 °C, 60 min) in H_2O/EtOH (50:50 vol. %) mixture and then is filtered off from the hot solution and dried under vacuum.

Characterization data for compounds 6a-h.

*Ethyl 2-(7-(4-fluorophenylcarbamoyl)-2-(4-methoxyphenyl)-1H-imidazo[1,2-b]pyrazol-3-ylamino)acetate (**6a**).* Yellowish solid, mp 159-161 °C. ^{1}H NMR (400 MHz, DMSO-d_6) δ = 11.65 (s, 1H, NH), 9.51 (s, 1H, NH), 7.80 (s, 1H, CH), 6.99-7.79 (m, 8H, ArH), 5.43 (s, 1H, NH), 4.14 (s, 2H, CH_2), 4.00 (q, *J* = 7.0 Hz, 2H, $\underline{CH_2}CH_3$), 3.78 (s, 3H, $\underline{CH_3}O$), 1.08 (t, *J* = 6.9 Hz, 3H, $CH_2\underline{CH_3}$) ppm; ^{13}C NMR (100 MHz, DMSO-d_6): δ = 171.3, 159.4 (d, *J* = 250.8 Hz), 156.4, 141.7, 137.5, 136.2, 127.9, 123.2, 122.6, 121.2 (d, *J* = 7.7 Hz), 116.3, 115.0 (d, *J* = 22.0 Hz), 113.9, 109.5, 94.1, 60.2, 55.1, 46.4, 14.0 ppm. HRMS (ESI/FT-ICR) calcd for $C_{23}H_{22}FN_5O_4$ $[M+H]^+$ 452.1734, found 452.1709.

*Ethyl 2-(2-(4-chlorophenyl)-7-(4-fluorophenylcarbamoyl)-1H-imidazo[1,2-b]pyrazol-3-ylamino)acetate (**6b**).* Yellowish solid, mp 149-151 °C. ^{1}H NMR (400 MHz, DMSO-d_6) δ = 11.79 (s, 1H, NH), 9.56 (s, 1H, NH), 7.90 (s, 1H, CH), 7.14-7.88 (m, 8H, ArH), 5.73 (s, 1H, NH), 4.22 (s, 2H, CH_2), 4.01 (q, *J* = 7.1 Hz, 2H, $\underline{CH_2}CH_3$), 1.08 (t, *J* = 7.1 Hz, 3H, $CH_2\underline{CH_3}$) ppm; ^{13}C NMR (100 MHz, DMSO-d_6): δ = 171.3, 160.5, 157.7 (d, *J* = 238.7 Hz), 142.1, 138.2, 136.1, 130.8, 129.1, 128.3, 127.9, 124.7, 121.2 (d, *J* = 7.6

Hz), 115.0 (d, J = 22.2 Hz), 114.5, 94.1, 60.2, 46.1, 13.9 ppm. HRMS (ESI/FT-ICR) calcd for $C_{22}H_{19}FN_5O_3$ $[M+H]^+$ 456.1239, found 456.1213.

*Methyl 4-(3-(2-ethoxy-2-oxoethylamino)-7-(4-fluorophenylcarbamoyl)-1H-imidazo[1,2-b]pyrazol-2-yl)benzoate (**6c**).* Yellowish solid, mp 189-190 °C. ^{1}H NMR (400 MHz, DMSO-d$_6$) δ = 11.83 (s, 1H, NH), 9.57 (s, 1H, NH), 8.24 (s, 1H, CH), 7.14-7.97 (m, 8H, ArH), 6.00 (s, 1H, NH), 4.30 (s, 2H, CH_2), 4.02 (q, J = 7.1 Hz, 2H, $\underline{CH_2}CH_3$), 3.85 (s, 3H, $CO_2\underline{CH_3}O$), 1.08 (t, J = 7.1 Hz, 3H, $CH_2\underline{CH_3}$) ppm; ^{13}C NMR (100 MHz, DMSO-d$_6$): δ = 171.3, 166.0, 160.5, 157.7 (d, J = 239.0 Hz), 142.7, 138.8, 136.1, 135.1, 129.3, 126.5, 126.3, 125.5, 121.3 (d, J = 7.6 Hz), 115.1 (d, J = 22.0 Hz), 113.6, 94.0, 60.3, 52.1, 45.9, 14.0 ppm. HRMS (ESI/FT-ICR) calcd for $C_{24}H_{22}FN_5O_5$ $[M+H]^+$ 480.1683, found 480.1654.

*Ethyl 2-(7-(4-fluorophenylcarbamoyl)-2-(4-nitrophenyl)-1H-imidazo[1,2-b]pyrazol-3-ylamino)acetate (**6d**).* Brown solid, mp 171-173 °C. ^{1}H NMR (400 MHz, DMSO-d$_6$) δ = 11.93 (s, 1H, NH), 9.62 (s, 1H, NH), 8.27 (s, 1H, CH), 7.14-8.23 (m, 8H, ArH), 6.31 (t, J = 6.5 Hz, 1H, NH), 4.37 (d, J = 6.0 Hz, 2H, CH_2), 4.03 (q, J = 7.1 Hz, 2H, $\underline{CH_2}CH_3$), 1.10 (t, J = 7.1 Hz, 3H, $CH_2\underline{CH_3}$) ppm; ^{13}C NMR (100 MHz, DMSO-d$_6$): δ = 171.2, 160.4, 157.7 (d, J = 239.1 Hz), 144.3, 143.1, 139.5, 137.2, 136.0, 127.7, 125.7, 123.7, 121.3 (d, J = 7.6 Hz), 115.1 (d, J = 22.1 Hz), 112.5, 94.0, 60.4, 45.8, 14.0 ppm. HRMS (ESI/FT-ICR) calcd for $C_{22}H_{19}FN_6O_5$ $[M-H]^+$ 465.1323, found 465.1307.

*Ethyl 2-(7-(3-fluorophenylcarbamoyl)-2-(4-methoxyphenyl)-1H-imidazo[1,2-b]pyrazol-3-ylamino)acetate (**6e**).* Yellowish solid, mp 124-126 °C. ^{1}H NMR (400 MHz, DMSO-d$_6$) δ = 11.72 (s, 1H, NH), 9.66 (s, 1H, NH), 8.22 (s, 1H, CH), 6.82-7.78 (m, 8H, ArH), 5.44 (s, 1H, NH), 4.14 (d, J = 5.5 Hz, 2H, CH_2), 4.01 (q, J = 7.1 Hz, 2H, $\underline{CH_2}CH_3$), 3.78 (s, 3H, $\underline{CH_3}O$), 1.09 (t, J = 7.1 Hz, 3H, $CH_2\underline{CH_3}$) ppm; ^{13}C NMR (100 MHz,

DMSO-d_6): δ = 171.3, 162.1 (d, *J* = 240.3 Hz), 160.8, 158.1, 141.8, 141.7, 137.6, 130.04 (d, *J* = 9.7 Hz), 128.0, 123.2, 122.5, 116.4, 115.0, 113.9, 108.7 (d, *J* = 21.2 Hz), 106.0 (d, *J* = 26.5 Hz), 94.1, 60.2, 55.2, 46.4, 14.0 ppm. HRMS (ESI/FT-ICR) calcd for $C_{23}H_{22}FN_5O_4$ $[M+H]^+$ 450.1578, found 450.1543.

*Ethyl 2-(2-(4-chlorophenyl)-7-(3-fluorophenylcarbamoyl)-1H-imidazo[1,2-b]pyrazol-3-ylamino)acetate (**6f**).* Yellowish solid, mp 153-155 °C. ^1H NMR (200 MHz, DMSO-d_6) δ = 11.87 (s, 1H, NH), 9.72 (s, 1H, NH), 7.91 (s, 1H, CH), 6.82-7.88 (m, 8H, ArH), 5.75 (s, 1H, NH), 4.22 (s, 2H, CH_2), 4.01 (q, *J* = 7.2 Hz, 2H, $\underline{CH_2}CH_3$), 1.08 (t, *J* = 6.9 Hz, 3H, $CH_2\underline{CH_3}$) ppm; ^{13}C NMR (100 MHz, DMSO-d_6): δ = 171.4, 162.2 (d, *J* = 241.0 Hz), 160.8, 142.2, 141.7 (d, J = 10.8 Hz), 138.4, 130.9, 130.1 (d, *J* = 10.1 Hz), 129.1, 128.4, 128.0, 124.8, 115.1, 114.6, 108.9 (d, *J* = 21.5 Hz), 106.1 (d, *J* = 25.3 Hz), 94.1, 60.3, 46.1, 14.0 ppm. HRMS (ESI/FT-ICR) calcd for $C_{22}H_{19}FN_5O_3$ $[M+H]^+$ 456.1239, found 456.1208.

*Methyl 4-(3-(2-ethoxy-2-oxoethylamino)-7-(3-fluorophenylcarbamoyl)-1H-imidazo[1,2-b]pyrazol-2-yl)benzoate (**6g**).* Yellowish solid, mp 171-172 °C. ^1H NMR (400 MHz, DMSO-d_6) δ = 11.89 (s, 1H, NH), 9.71 (s, 1H, NH), 8.27 (s, 1H, CH), 6.82-8.02 (m, 8H, ArH), 6.01 (s, 1H, NH), 4.30 (s, 2H, CH_2), 4.02 (q, *J* = 7.1 Hz, 2H, $\underline{CH_2}CH_3$), 3.85 (s, 3H, $CO_2\underline{CH_3}O$),1.08 (t, *J* = 7.1 Hz, 3H, $CH_2\underline{CH_3}$) ppm; ^{13}C NMR (100 MHz, DMSO-d_6): δ = 171.7, 166.4, 162.6 (d, *J* = 240.3 Hz), 161.2, 143.1, 142.1 (d, *J* = 11.3 Hz), 139.3, 135.5, 130.5 (d, *J* = 9.5 Hz), 129.7, 127.0, 126.7, 126.0, 115.5, 114.1, 109.3 (d, *J* = 21.2 Hz), 106.5 (d, *J* = 26.3 Hz), 94.4, 60.7, 52.4, 46.4, 14.4 ppm. HRMS (ESI/FT-ICR) calcd for $C_{24}H_{22}FN_5O_5$ $[M+H]^+$ 480.1683, found 480.1661.

*Ethyl 2-(7-(3-fluorophenylcarbamoyl)-2-(4-nitrophenyl)-1H-imidazo[1,2-b]pyrazol-3-ylamino)acetate (**6h**).* Brown solid, mp 183-185 °C. ^1H NMR (400 MHz, DMSO-d_6) δ

= 11.98 (s, 1H, NH), 9.75 (s, 1H, NH), 8.30 (s, 1H, CH), 6.83-8.23 (m, 8H, ArH), 6.31 (t, *J* = 6.7 Hz, 1H, NH), 4.37 (d, *J* = 6.3 Hz, 2H, CH_2), 4.03 (q, *J* = 7.1 Hz, 2H, $\underline{CH_2}CH_3$), 1.09 (t, *J* = 7.1 Hz, 3H, $CH_2\underline{CH_3}$) ppm; ^{13}C NMR (100 MHz, DMSO-d_6): δ = 171.3, 162.2 (d, *J* = 240.4 Hz), 160.7, 144.4, 143.2, 141.6 (d, *J* = 11.1 Hz), 139.6, 137.2, 130.2 (d, *J* = 9.8 Hz), 127.8, 125.7, 123.8, 115.1, 112.5, 109.0 (d, *J* = 21.6 Hz), 106.2 (d, *J* = 26.5 Hz), 94.0, 60.5, 45.8, 14.0 ppm. HRMS (ESI/FT-ICR) calcd for $C_{22}H_{19}FN_6O_5$ $[M+H]^+$ 467.1479, found 467.1457.

General procedure for the synthesis of *N*-(1-arylethyl-2-(*tert*-butylamino)-2-oxo)-*N*-(5-methylisoxazol-3-yl)-3-phenylpropiolamides 9a–g.

3-Amino-5-methylisoxazole (**7**) (1 mmol) and an appropriate aromatic aldehyde **1a-h** (1 mmol) were dissolved in MeOH (6 mL) and stirred for 1 h. Then phenylpropiolic acid (**8**) (1 mmol) and 1 mmol of *tert*-butylisocyanide (**3a**) were added successively and the reaction was stirred for 24 h till completion. After evaporating the solvent, the residue was treated with an H_2O/EtOH (50:50, vol. %, 6 mL) mixture and ultrasonicated for 15 min. The formed precipitate was filtered off and dried under vacuum.

Characterization data for compounds 9a–g.

*N-(2-(tert-Butylamino)-2-oxo-1-phenylethyl)-N-(5-methylisoxazol-3-yl)-3-phenylpropiolamide (**9a**).* Colorless solid, mp 134-136 °C. ^{1}H NMR (400 MHz, DMSO-d_6) δ = 7.97 (s, 1H, NH), 7.15-7.53 (m, 10H, ArH), 6.37 (s, 1H, CH), 6.09 (s, 1H, CH), 2.29 (s, 3H, CH_3), 1.24 (s, 9H, $\underline{(CH_3)_3}C$) ppm; ^{13}C NMR (100 MHz, DMSO-d_6): δ = 170.5, 167.2, 159.9, 152.7, 134.3, 132.2, 130.9, 129.5, 129.0, 128.2, 128.1, 119.0, 103.3, 90.1, 81.7, 62.5, 50.6, 28.3, 12.1 ppm. HRMS (EI) calcd for $C_{25}H_{25}N_3O_3$ $[M]^+$ 415.18959, found 415.20193.

N-(2-(tert-Butylamino)-1-(2-methoxyphenyl)-2-oxoethyl)-N-(5-methylisoxazol-3-yl)-3-phenylpropiolamide (***9b***). Colorless solid, mp 132-133 °C. ^{1}H NMR (300 MHz, DMSO-d_6) δ = 7.95 (s, 1H, NH), 6.77-7.55 (m, 9H, ArH), 6.28 (s, 1H, CH), 6.24 (s, 1H, CH), 3.78 (s, 3H, $\underline{CH_3}$O), 2.26 (s, 3H, CH_3), 1.24 (s, 9H, $\underline{(CH_3)_3}$C) ppm; ^{13}C NMR (150 MHz, DMSO-d_6): δ = 170.3, 167.7, 159.9, 157.6, 152.4, 132.1, 130.8, 130.0, 129.2, 129.00, 122.3, 119.7, 119.1, 110.9, 103.1, 89.6, 81.8, 57.5, 55.5, 50.5, 28.3, 12.0 ppm. HRMS (EI) calcd for $C_{26}H_{27}N_3O_4$ $[M]^+$ 445.20016, found 445.19872.

N-(2-(tert-Butylamino)-1-(3-methoxyphenyl)-2-oxoethyl)-N-(5-methylisoxazol-3-yl)-3-phenylpropiolamide (***9c***). Colorless solid, mp 126-128 °C. ^{1}H NMR (300 MHz, DMSO-d_6) δ = 7.98 (s, 1H, NH), 6.71-7.46 (m, 9H, ArH), 6.40 (s, 1H, CH), 6.05 (s, 1H, CH), 3.66 (s, 3H, $\underline{CH_3}$O), 2.31 (s, 3H, CH_3), 1.23 (s, 9H, $\underline{(CH_3)_3}$C) ppm; ^{13}C NMR (75 MHz, DMSO-d_6): δ = 170.5, 167.2, 159.8, 158.8, 152.7, 135.7, 132.3, 131.0, 129.2, 129.0, 121.8, 119.0, 115.2, 113.7, 103.3, 90.3, 81.7, 62.6, 55.0, 50.6, 28.2, 12.1 ppm. HRMS (EI) calcd for $C_{26}H_{27}N_3O_4$ $[M]^+$ 445.20016, found 445.19759.

N-(2-(tert-Butylamino)-1-(4-methoxyphenyl)-2-oxoethyl)-N-(5-methylisoxazol-3-yl)-3-phenylpropiolamide (***9d***). Colorless solid, mp 149-151 °C. ^{1}H NMR (400 MHz, DMSO-d_6) δ = 7.88 (s, 1H, NH), 6.80-7.55 (m, 9H, ArH), 6.36 (s, 1H, CH), 6.00 (s, 1H, CH), 3.68 (s, 3H, $\underline{CH_3}$O), 2.30 (s, 3H, CH_3), 1.23 (s, 9H, $\underline{(CH_3)_3}$C) ppm; ^{13}C NMR (75 MHz, DMSO-d_6): δ = 170.5, 167.7, 160.00, 158.9, 152.7, 132.2, 130.9, 129.00, 125.9, 119.00, 113.5, 103.4, 89.9, 81.7, 61.9, 55.0, 50.5, 28.3, 12.1 ppm. HRMS (EI) calcd for $C_{26}H_{27}N_3O_4$ $[M]^+$ 445.20016, found 445.19863.

N-(2-(tert-Butylamino)-1-(4-chlorophenyl)-2-oxoethyl)-N-(5-methylisoxazol-3-yl)-3-phenylpropiolamide (***9e***). Colorless solid, mp 148-150 °C. ^{1}H NMR (300 MHz, DMSO-d_6) δ = 8.00 (s, 1H, NH), 7.54 – 7.18 (m, 9H, ArH), 6.42 (s, 1H, CH), 6.07 (s, 1H, CH),

2.32 (s, 3H, CH_3), 1.22 (s, 9H, $(CH_3)_3C$) ppm; ^{13}C NMR (150 MHz, DMSO-d_6): δ = 171.1, 166.8, 159.8, 152.7, 133.5, 132.9, 132.2, 131.4, 131.0, 129.0, 128.2, 118.9, 103.5, 90.3, 81.5, 61.8, 50.6, 28.2, 12.1 ppm. HRMS (EI) calcd for $C_{25}H_{24}ClN_3O_3$ $[M]^+$ 449.15062, found 449.14901.

*Methyl 4-(2-(tert-Butylamino)-1-(N-(5-methylisoxazol-3-yl)-3-phenylpropiolamido)-2-oxoethyl)benzoate (**9f**).* Colorless solid, mp 136-137 °C. ^1H NMR (300 MHz, DMSO-d_6) δ = 8.04 (s, 1H, NH), 7.21-7.85 (m, 9H, ArH), 6.43 (s, 1H, CH), 6.16 (s, 1H, CH), 3.81 (s, 3H, CO_2CH_3O), 2.31 (s, 3H, CH_3), 1.22 (s, 9H, $(CH_3)_3C$) ppm; ^{13}C NMR (100 MHz, DMSO-d_6): δ = 170.9, 166.6, 165.8, 159.8, 152.7, 139.8, 132.3, 131.0, 129.9, 129.3, 129.0, 128.9, 118.9, 103.4, 90.5, 81.5, 62.3, 52.1, 50.7, 28.2, 12.1 ppm. HRMS (ESI/FT-ICR) calcd for $C_{27}H_{27}N_3O_5$ $[M+H]^+$ 474.2029, found 474.2032.

*N-(2-(tert-Butylamino)-1-(4-cyanophenyl)-2-oxoethyl)-N-(5-methylisoxazol-3-yl)-3-phenylpropiolamide (**9h**).* Colorless solid, mp 119-121 °C. ^1H NMR (300 MHz, DMSO-d_6) δ = 8.09 (s, 1H, NH), 7.25-7.77 (m, 9H, Ar), 6.45 (s, 1H, CH), 6.16 (s, 1H, CH), 2.34 (s, 3H, CH_3), 1.20 (s, 9H, $(CH_3)_3C$) ppm; HRMS (EI) calcd for $C_{26}H_{24}N_3O_3$ $[M]^+$ 440.18484, found 440.18146.

3. X-ray analysis

The crystals of **4e** ($C_{22}H_{21}FClN_5O·H_2O$) are monoclinic. At 293 K, a = 10.9799(4) Å, b = 15.7291(5) Å, c = 13.1112(5) Å, β = 105.336(4)°, V = 2183.7(1) $Å^3$, M_r = 443.90, Z = 4, space group $P2_1/c$, d_{calc} = 1.350 g/cm^3, μ(MoK_α) = 0.212 mm^{-1}, F(000) = 928.

The crystals of **9e** ($C_{25}H_{24}ClN_3O_3$, 0.17(CH_2Cl_2)) are trigonal. At 293 K a = b = 34.6375(8) Å, c = 10.7015(2) Å, V = 11119.1(4) $Å^3$, M_r = 464.08, Z = 18, space group R $\bar{3}$, d_{calc} = 1.248 g/cm^3, μ(MoK_α) = 0.221 mm^{-1}, F(000) = 4374.

Intensities of 20702 reflections (3853 independent, R_{int} = 0.028) for **4e** and 46110 reflections (4340 independent, R_{int} = 0.056) for **9e** were measured on an Xcalibur 3 diffractometer (graphite monochromated MoK_α radiation, CCD-detector, ω scanning, 2Θmax = 50°).

The structures were solved by direct method using SHELXTL package [2]. Positions of hydrogen atoms were located from electron density difference maps and refined using riding model with $U_{iso} = nU_{eq}$ (n = 1.5 for methyl groups and water molecule and 1.2 for other hydrogen atoms). The hydrogen atoms of the amino groups were refined isotropically in structure **9e**. Full-matrix least-squares refinement against F^2 in anisotropic approximation for non-hydrogen atoms was converged to wR_2 = 0.122 for 3848 reflections (R_1 = 0.041 for 3116 reflections with $F > 4\sigma(F)$, S = 1.062) in the case of **4e**, and wR_2 = 0.148 for 4340 reflections (R_1 = 0.048 for 3179 reflections with F > 4σ(F), S = 1.084) in the case of **9e**.

Final atomic coordinates, geometrical parameters and crystallographic data have been deposited with the Cambridge Crystallographic Data Centre, 11 Union Road, Cambridge, CB2 1EZ, UK (E-mail: deposit@ccdc.cam.ac.uk; fax: +441223336033) and are available on request quoting the deposition numbers 1503406 (**4e**) and 1503405 (**9e**).

4. Biology

Study of antimicrobial activities of the compounds was performed in vitro by the microdilution method. In order to prepare microdilution trays two-fold serial diluting the solutions of antimicrobial agents in a liquid culture medium (Mueller–Hinton broth (MHB)), ("HiMedia Laboratories", India) was made. Disposable polystyrene 96-well cell culture plates for immunological studies (DELTALAB, Spain) and Proline Plus 8-channel mechanical pipettes were used during the experiment. The analyses were carried

out according to recommendations of CLSI (Clinical and Laboratory Standards Institute, USA) [3–6].

In the beginning the solutions of the compounds studied in concentrations 500, 250, 125, 62.5, 31.25 and 15.6 mg/l were put into the wells of the plate using the two-fold serial dilutions method (the total volume of 200 μl was constant). Collectable reference strains of bacteria were used as test cultures: *Bacillus subtilis* (strain 1211), *Staphylococcus aureus* (strain 2231) – gram-positive cultures; *Escherichia coli* (strain 1257) and *Pseudomonas aeruginosa* (strain 1111) – gram-negative cultures. For preparation of an inoculum from the isolated colonies of microorganisms being in the phase of exponential growth a suspension in a saline was made according to McFarland standard (0.5). The suspension was diluted with MHB to concentration of 10^5 CFU/ml (colony-forming unit in 1 ml) and 100 μl of it was put to the walls of the plate that already contained 100 μl of corresponding solutions of the compounds studied. The wells containing only the suspensions diluted with MHB without adding any substance served as the "negative control". Sterility of the medium was controlled using the dedicated wells in microtiter plate containing neither the solution of the substance nor microbial suspension. Inoculated plates were incubated at 37 °C for 18 ± 2 hours.

The culture growth was assessed visually and spectrophotometrically comparing the growth of a microorganism in the presence of the compound studied with the growth of the culture in the well without the compound. The minimum inhibitory concentration (MIC) was defined as the lowest concentration of the antimicrobial agent (mg/l) able to inhibit any visible growth of the culture. The minimum bactericidal concentration (MBC) was determined by sowing out the content of those tubes with the absence of any signs of growth on peptone-meat extract agar in Petri dishes. The absorbance of the medium

during the growth of the cultures was measured with the help of microplate absorbance reader Sunrise RC (Tecan Austria GmbH, Switzerland) at 492 nm (reference wavelength 620 nm).

Nitroxoline, the quinoline based drug, was used as a comparison standard.

References

(1) Komikhov, S. O.; Petrova, M. H.; Desenko, S. M.; Afanasiadi, L. M. Derivatives of 3-amino-4-carboxamido-1*H*-pyrazole and the process for preparation thereof. Patent UA 82262, March 25, 2008. http://base.uipv.org/searchINV/search.php?action=viewdetails&IdClaim=27376).

(2) Sheldrick, G. M. *Acta Crystallogr. Sect. A Found. Crystallogr.* **2008**, *A64*, 112–122.

(3) CLSI. *Methods for Dilution Antimicrobial Susceptibility Tests for Bacteria That Grow Aerobically; Approved Standard—Tenth Edition.* CLSI document M07-A10. Wayne, PA: Clinical and Laboratory Standards Institute; **2015**. http://shop.clsi.org/microbiology-documents/M07-M100-PK.html

(4) ISO 20776-1:2006. *Clinical laboratory testing and in vitro diagnostic test systems - Susceptibility testing of infectious agents and evaluation of performance of antimicrobial susceptibility test devices - Part 1: Reference method for testing the in vitro activity of antimicrobial agents against rapidly growing aerobic bacteria involved in infectious diseases*; **2006**. https://www.iso.org/standard/41630.html

(5) Eucast Definitive Document. *Methods for the determination of susceptibility of bacteria to antimicrobial agents. Terminology. Clin. Microbiol. Infect.* **1998**, *4*(5), 291-296. http://www.sciencedirect.com/science/article/pii/S1198743X14650473

(6) Cornaglia, G.; Hryniewicz, W.; Jarlier, V.; Kahlmeter, G.; Mittermayer, H.; Stratchounski, L.; Baquero, F. *Clin. Microbiol. Infect.* **2004**, *10*, 349–383.
