# Supplementary material for: New tricks of well-known aminoazoles in isocyanide-based multicomponent reactions and antibacterial activity of the compounds synthesized
Source: Beilstein J Org Chem. 2017 May 31;13:1050–63. doi: 10.3762/bjoc.13.104 (PMC5480327; doi:10.3762/bjoc.13.104)

Supporting Information File 2

for

New tricks of well-known aminoazoles in isocyanide-based multicomponent reactions and antibacterial activity of the compounds synthesized

Maryna V. Murlykina[1,2], Maryna N. Kornet[3], Sergey M. Desenko[1,4], Svetlana V. Shishkina[1,4], Oleg V. Shishkin[1], Aleksander A. Brazhko[3], Vladimir I. Musatov[1], Erik V. Van der Eycken[2] and Valentin A. Chebanov[*,1,2,4]

Address: [a]Division of Chemistry of Functional Materials, State Scientific Institution "Institute for Single Crystals" of National Academy of Sciences of Ukraine, Nauky Ave., 60, 61001, Kharkiv, Ukraine, [b]Laboratory for Organic & Microwave-Assisted Chemistry (LOMAC), KU Leuven, Celestijnenlaan 200F, B-3001, Leuven, Belgium, [c]Laboratory of Biotechnology of Physiologically Active Substances, Zaporizhzhya National University, Zhukovsky str., 66, Zaporizhzhya, Ukraine, 69600 and [d]Faculty of Chemistry, V. N. Karazin Kharkiv National University, Svobody sq., 4, 61077, Kharkiv, Ukraine

Email: Valentin A. Chebanov - chebanov@isc.kharkov.com

*Corresponding author

NMR spectra

^{1}H and ^{13}C NMR spectra for compounds synthesized

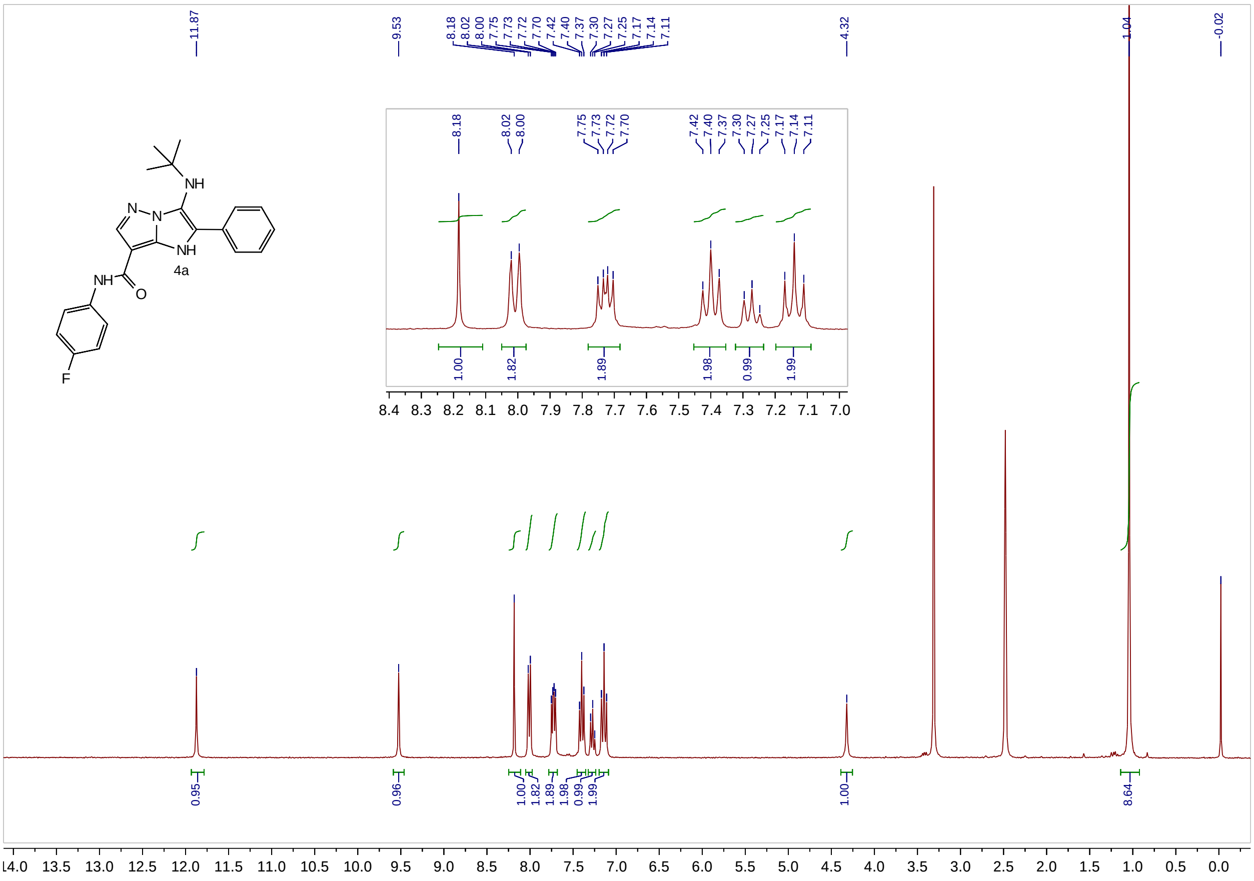

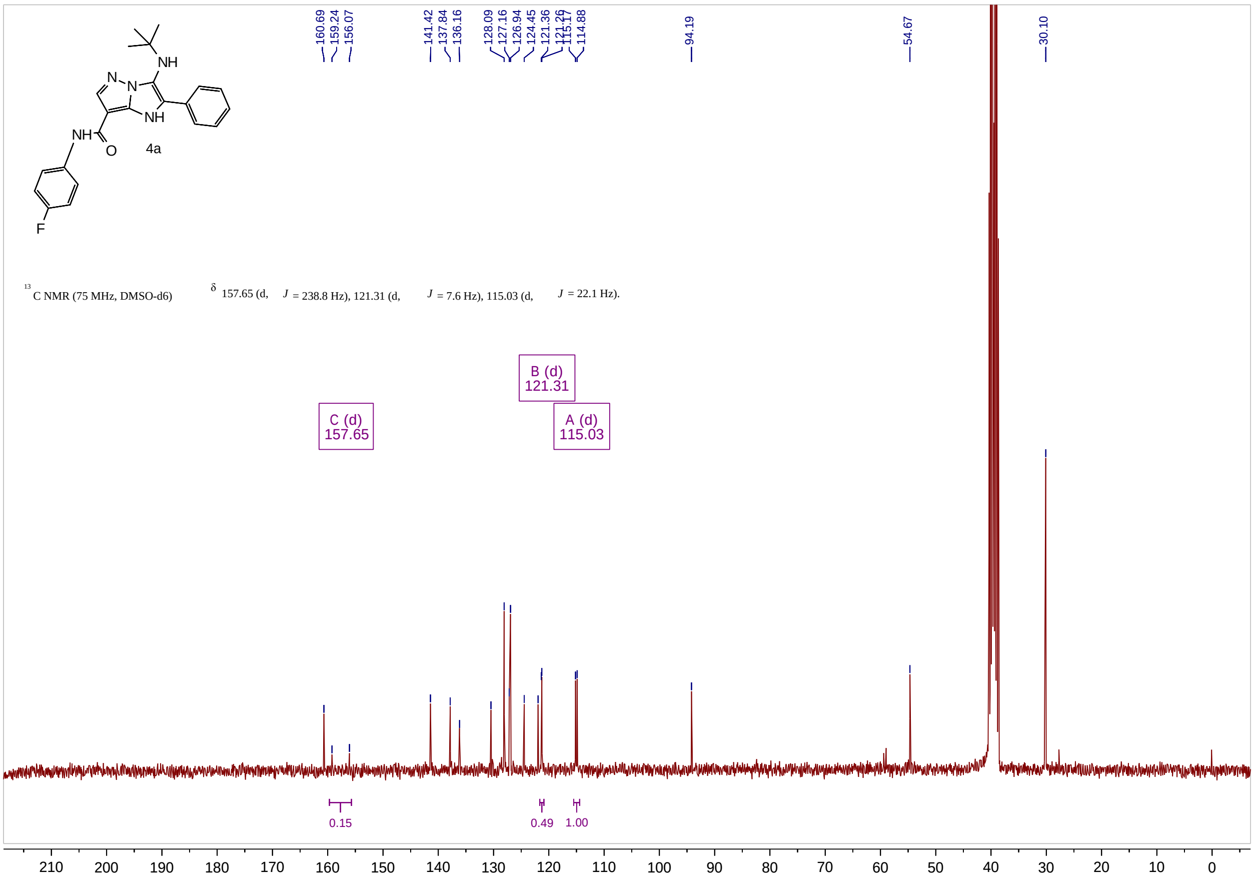

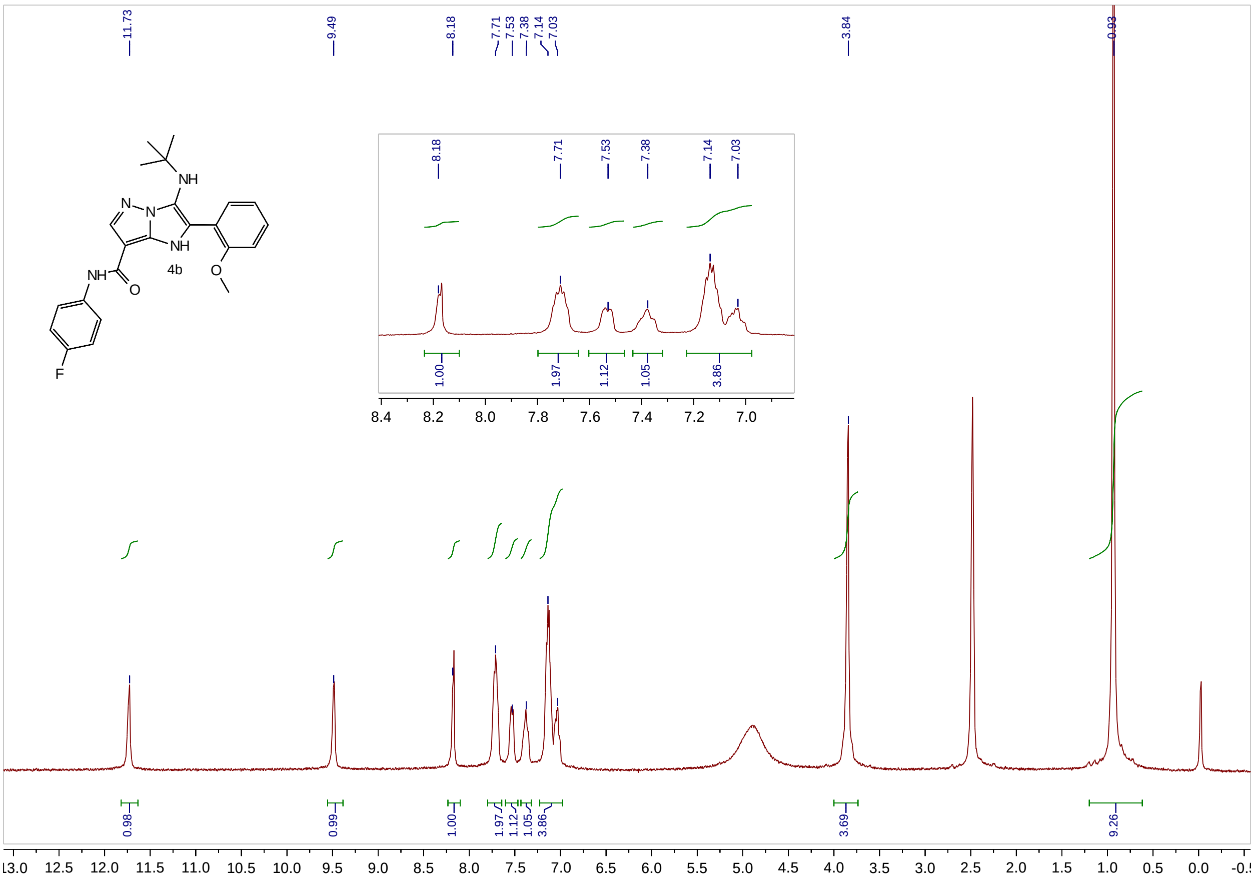
11.73
9.49
8.18
7.71
7.53
7.38
7.14
7.03
3.84
0.93
4b
NH
N
O
F
1.00
1.97
1.12
1.05
3.86
0.98
0.99
3.69
9.26
8.4 8.2 8.0 7.8 7.6 7.4 7.2 7.0
13.0 12.5 12.0 11.5 11.0 10.5 10.0 9.5 9.0 8.5 8.0 7.5 7.0 6.5 6.0 5.5 5.0 4.5 4.0 3.5 3.0 2.5 2.0 1.5 1.0 0.5 0.0

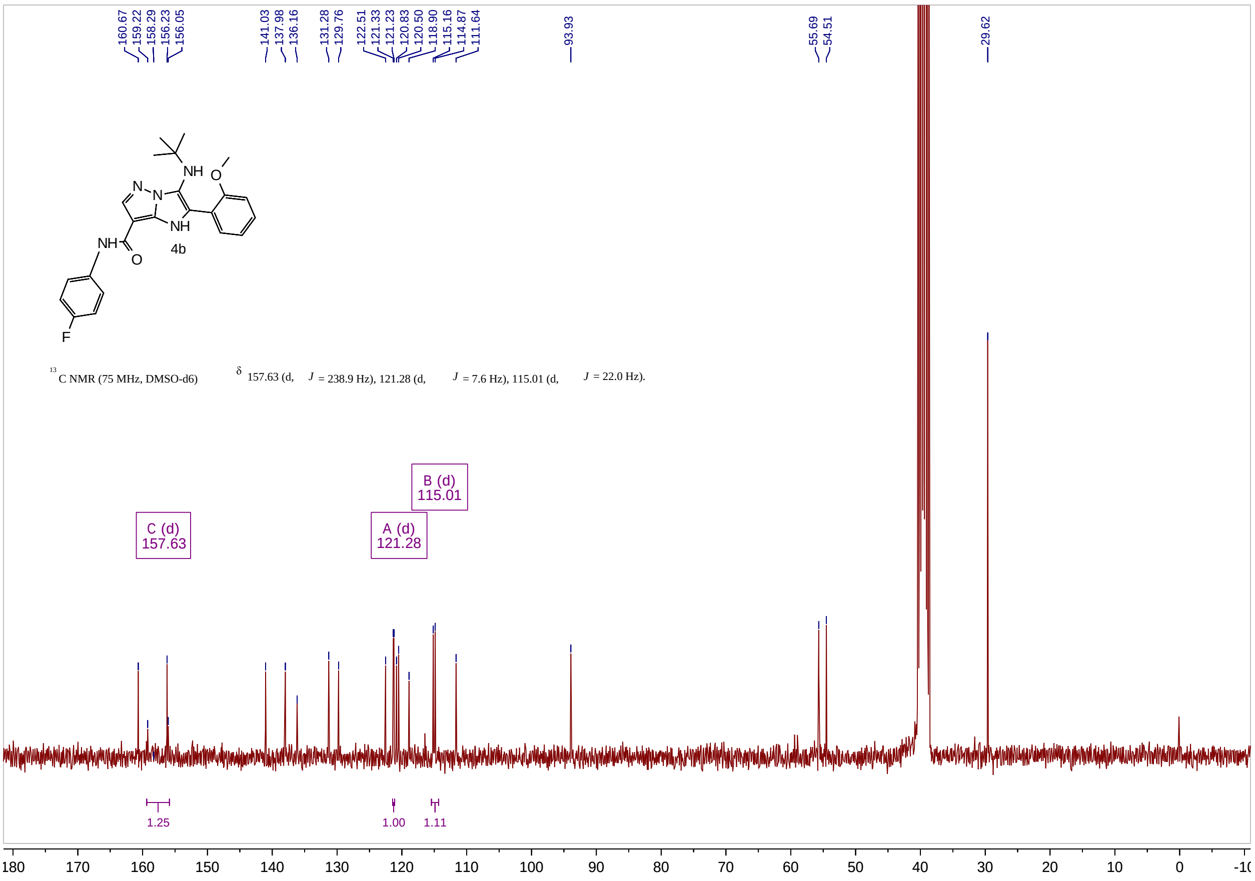
160.67
159.22
158.29
156.23
156.05
141.03
137.98
136.16
131.28
129.76
122.51
121.33
121.23
120.83
120.50
118.90
115.16
114.87
111.64
93.93
55.69
54.51
29.62
4b
NH
N
O
F
13C NMR (75 MHz, DMSO-d6) δ 157.63 (d, J = 238.9 Hz), 121.28 (d, J = 7.6 Hz), 115.01 (d, J = 22.0 Hz).
B (d)
115.01
C (d)
157.63
A (d)
121.28
1.25
1.00
1.11
180 170 160 150 140 130 120 110 100 90 80 70 60 50 40 30 20 10 0

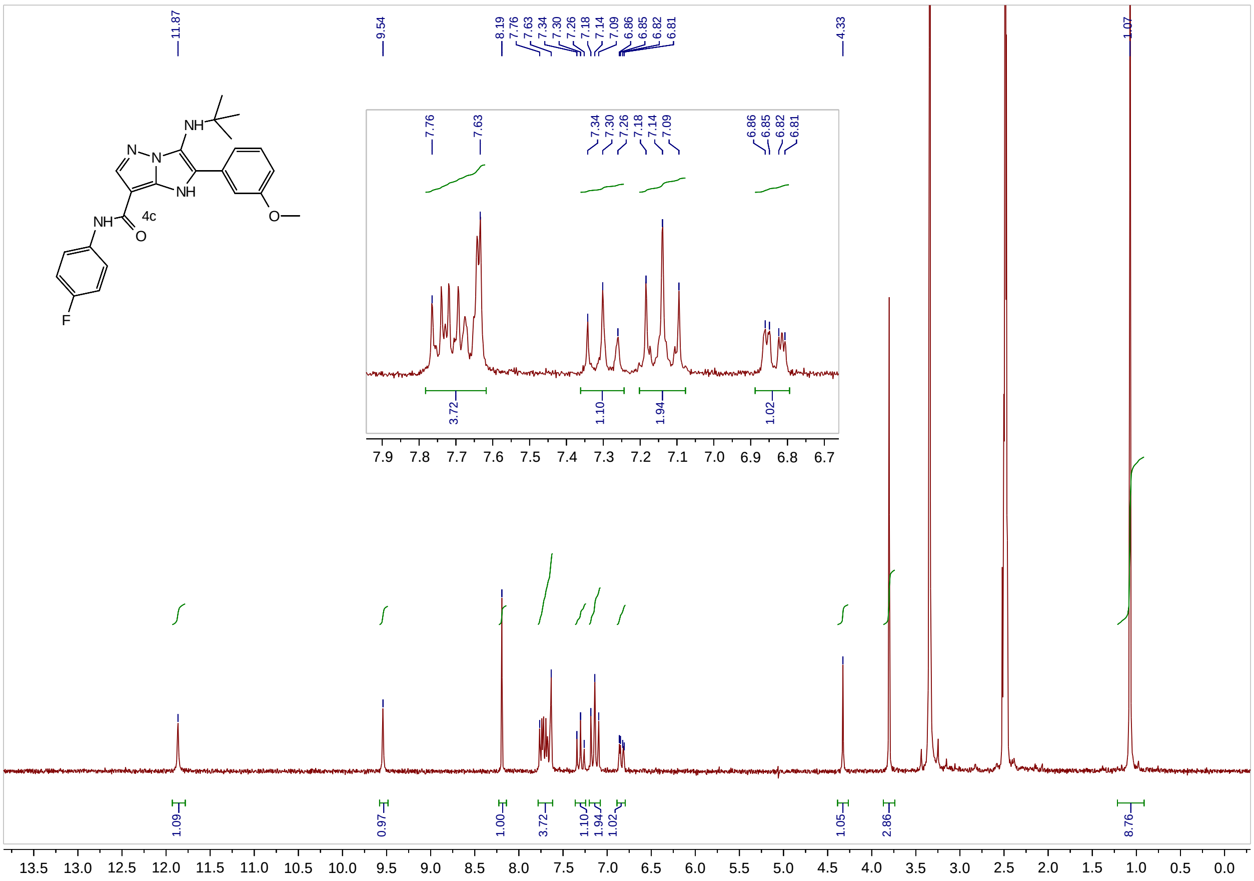

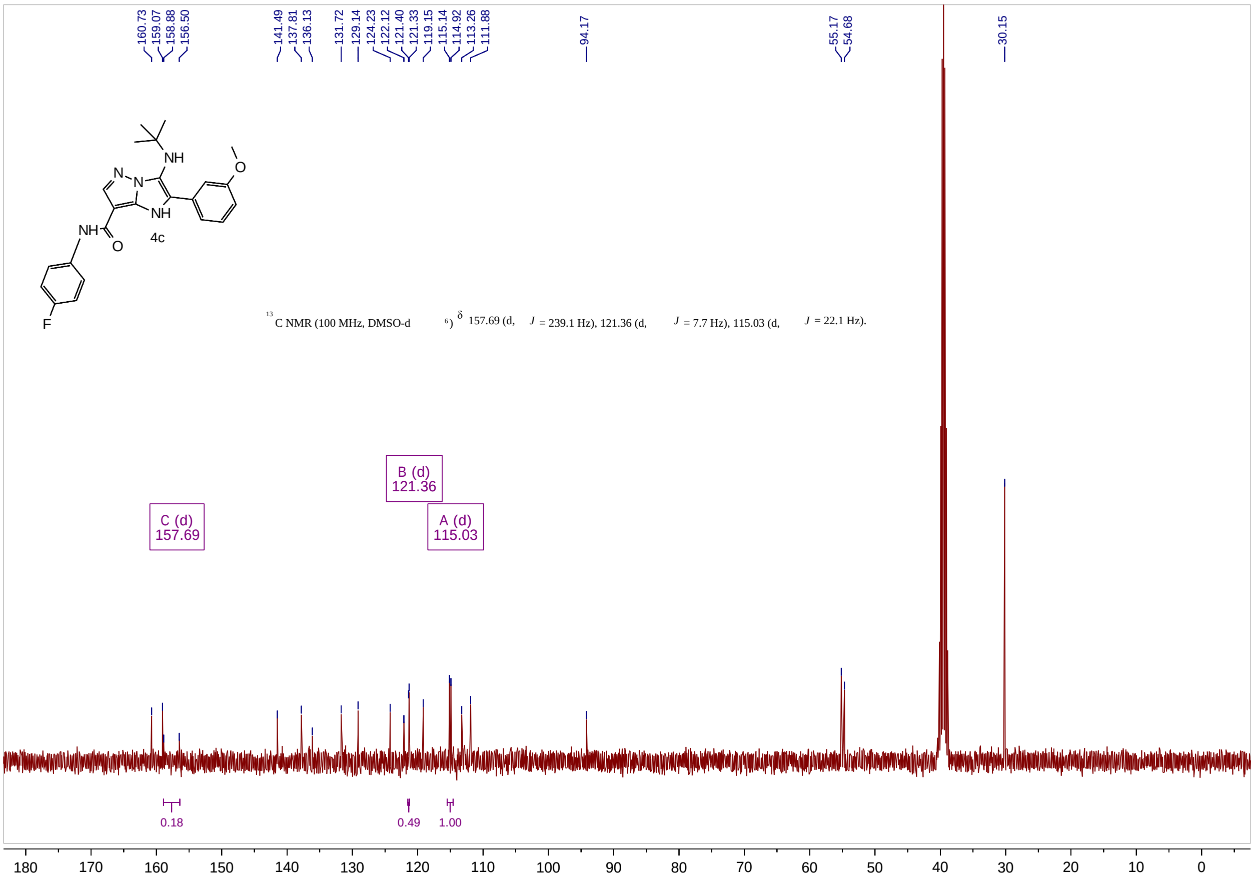

[13] C NMR (100 MHz, DMSO-d[6]) δ 157.69 (d, *J* = 239.1 Hz), 121.36 (d, *J* = 7.7 Hz), 115.03 (d, *J* = 22.1 Hz).

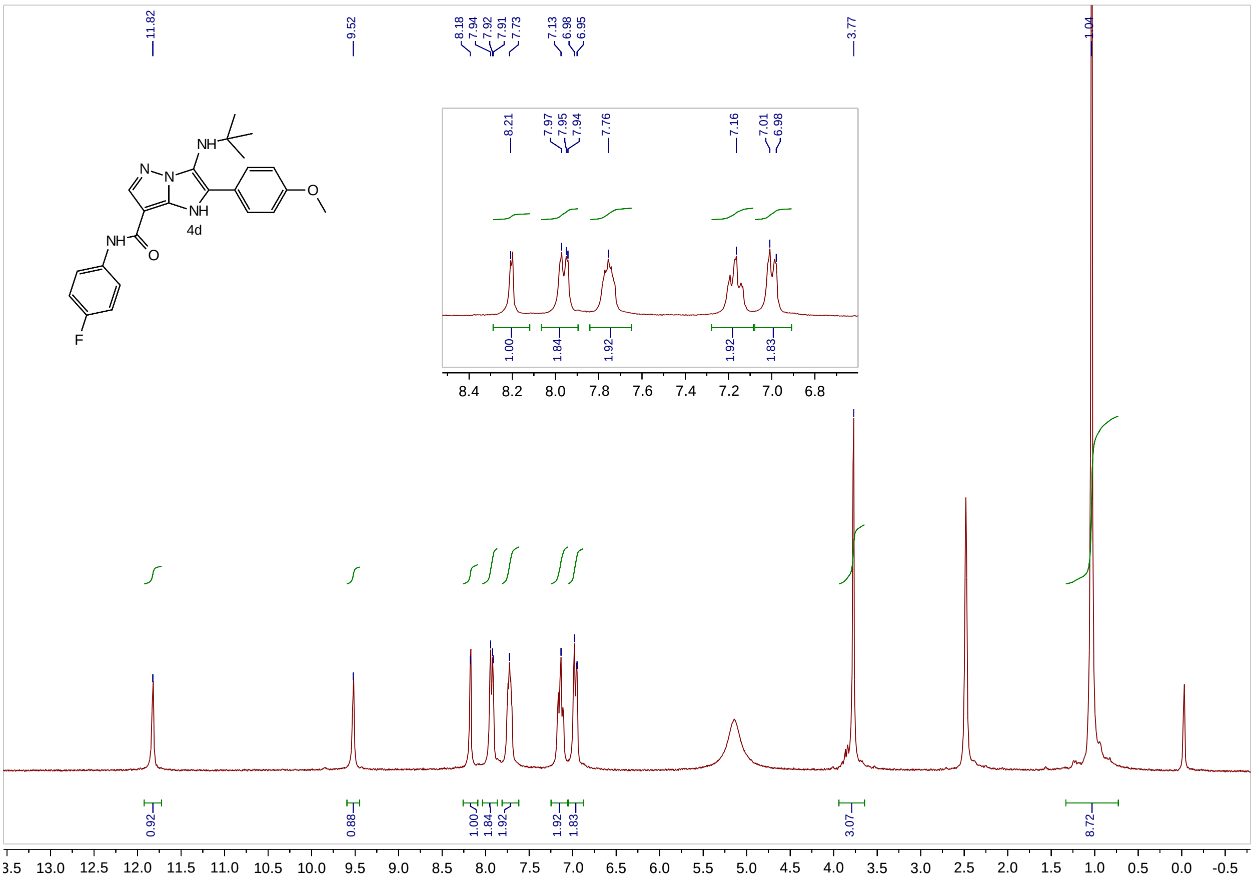
11.82
9.52
8.18
7.94
7.92
7.91
7.73
7.13
6.98
6.95
3.77
1.04
NH
N
N
NH
O
4d
NH
O
F
8.21
7.97
7.95
7.94
7.76
7.16
7.01
6.98
1.00
1.84
1.92
1.92
1.83
0.92
0.88
1.00
1.84
1.92
1.92
1.83
3.07
8.72

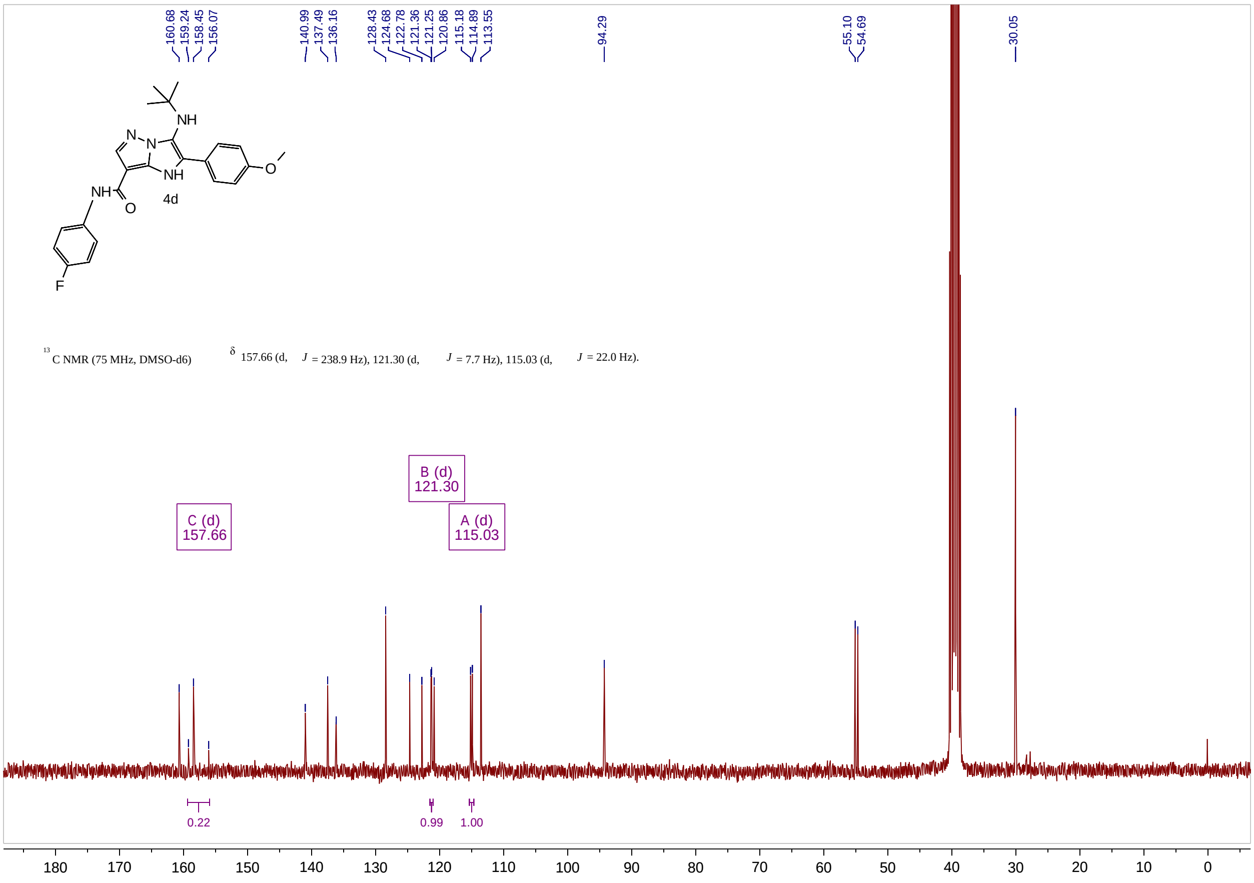
160.68
159.24
158.45
156.07
140.99
137.49
136.16
128.43
124.68
122.78
121.36
121.25
120.86
115.18
114.89
113.55
94.29
55.10
54.69
30.05
NH
N
N
NH
O
4d
NH
O
F
13C NMR (75 MHz, DMSO-d6) δ 157.66 (d, J = 238.9 Hz), 121.30 (d, J = 7.7 Hz), 115.03 (d, J = 22.0 Hz).
B (d)
121.30
C (d)
157.66
A (d)
115.03
0.22
0.99
1.00

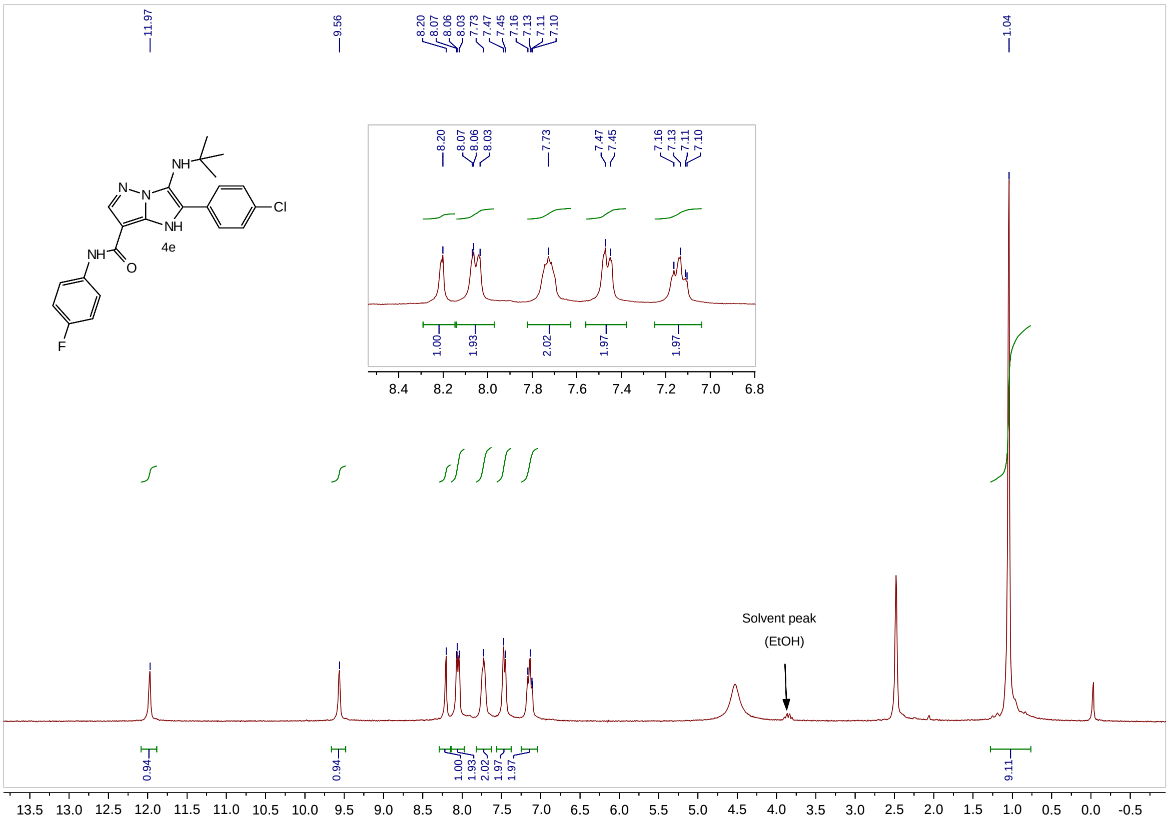
11.97
9.56
8.20 8.07 8.06 8.03 7.73 7.47 7.45 7.16 7.13 7.11 7.10
1.04
NH
N
N
Cl
NH
4e
NH
O
F
Solvent peak
(EtOH)
0.94
0.94
1.00 1.93 2.02 1.97 1.97
9.11
13.5 13.0 12.5 12.0 11.5 11.0 10.5 10.0 9.5 9.0 8.5 8.0 7.5 7.0 6.5 6.0 5.5 5.0 4.5 4.0 3.5 3.0 2.5 2.0 1.5 1.0 0.5 0.0 -0.5

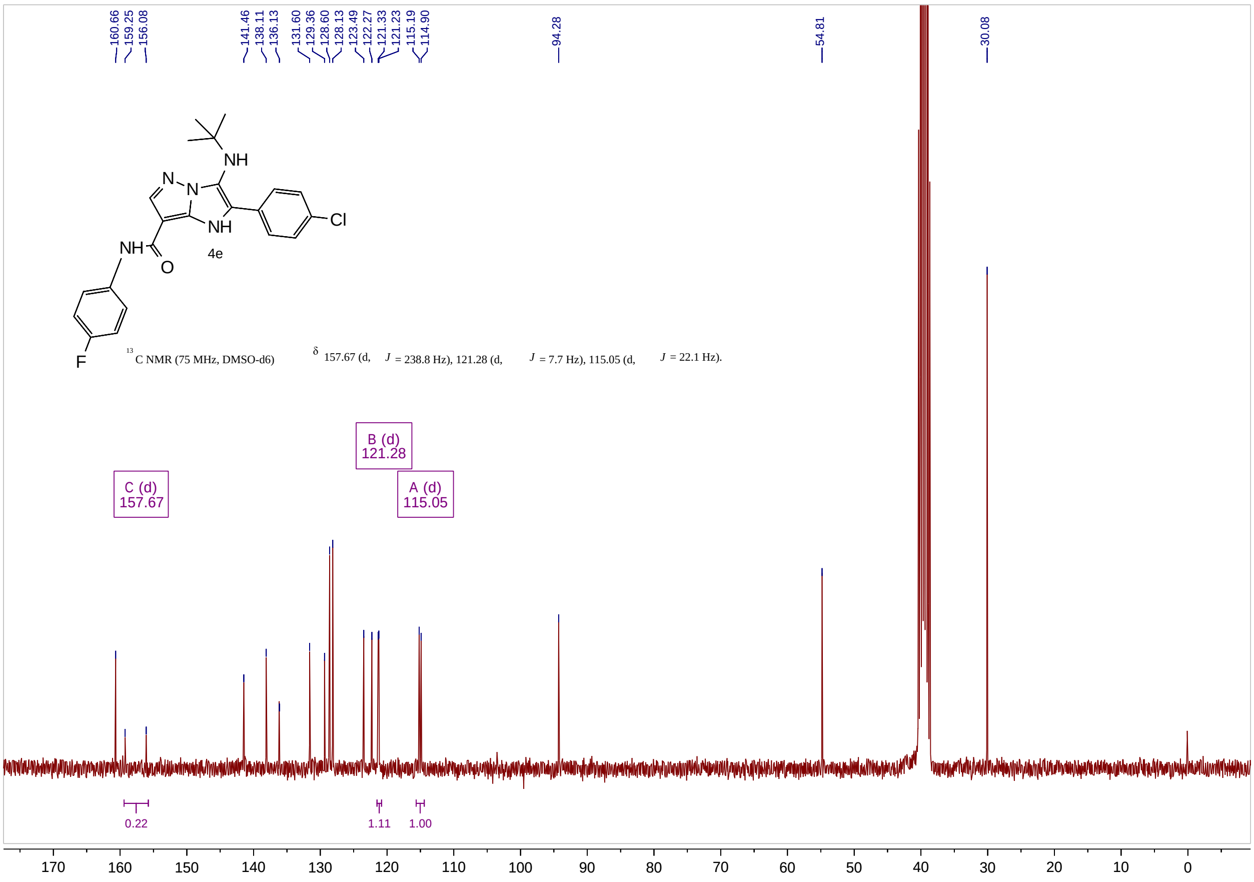
160.66 159.25 156.08
141.46 138.11 136.13 131.60 129.36 128.60 128.13 123.49 122.27 121.33 121.23 115.19 114.90
94.28
54.81
30.08
NH
N
N
Cl
NH
4e
NH
O
F
13C NMR (75 MHz, DMSO-d6) δ 157.67 (d, J = 238.8 Hz), 121.28 (d, J = 7.7 Hz), 115.05 (d, J = 22.1 Hz).
B (d) 121.28
C (d) 157.67
A (d) 115.05
0.22
1.11 1.00
170 160 150 140 130 120 110 100 90 80 70 60 50 40 30 20 10 0

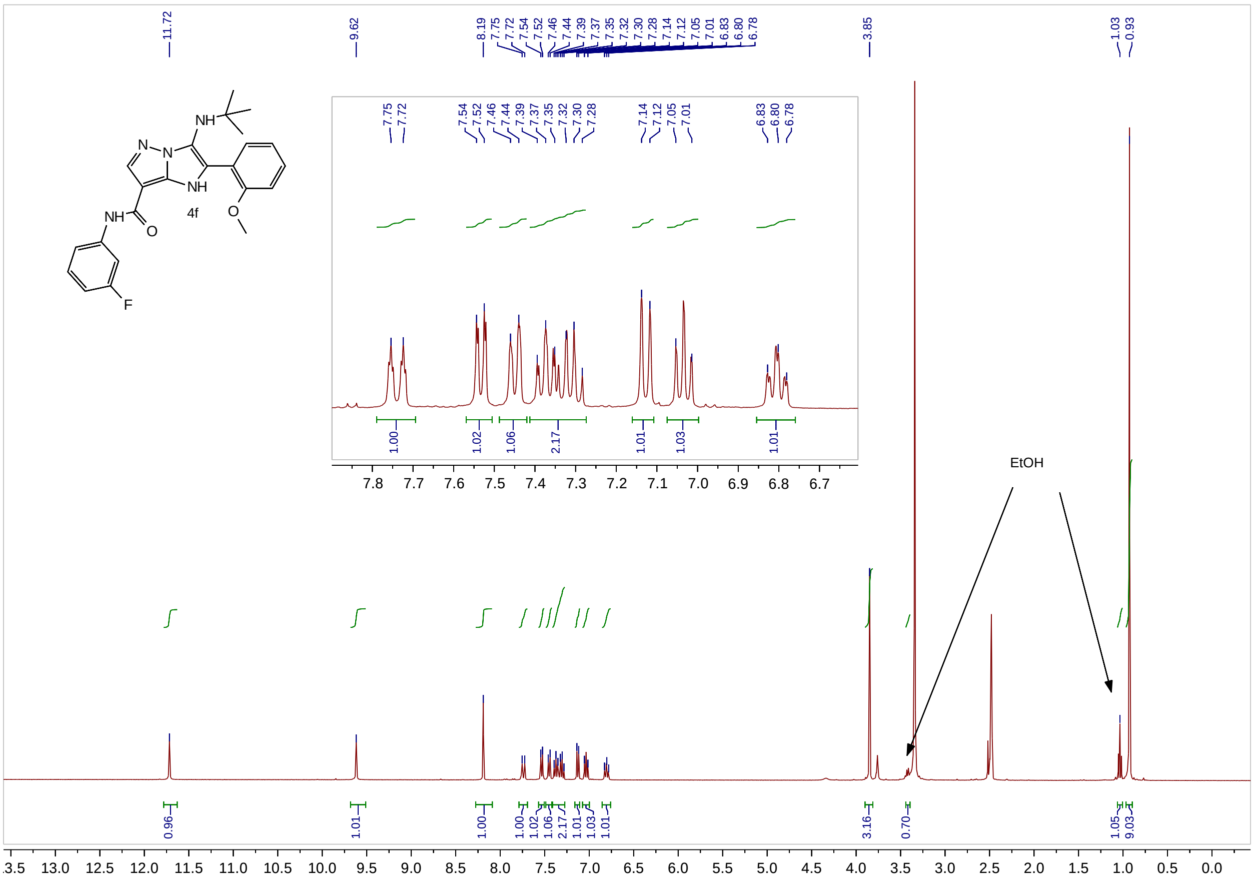
11.72
9.62
8.19 7.75 7.72 7.54 7.52 7.46 7.44 7.39 7.37 7.35 7.32 7.30 7.28 7.14 7.12 7.05 7.01 6.83 6.80 6.78
3.85
1.03 0.93
NH
N
N
NH
NH
O
O
F
4f
7.75 7.72 7.54 7.52 7.46 7.44 7.39 7.37 7.35 7.32 7.30 7.28 7.14 7.12 7.05 7.01 6.83 6.80 6.78
1.00 1.02 1.06 2.17 1.01 1.03 1.01
EtOH
0.96 1.01 1.00 1.00 1.02 1.06 2.17 1.01 1.03 1.01 3.16 0.70 1.05 9.03

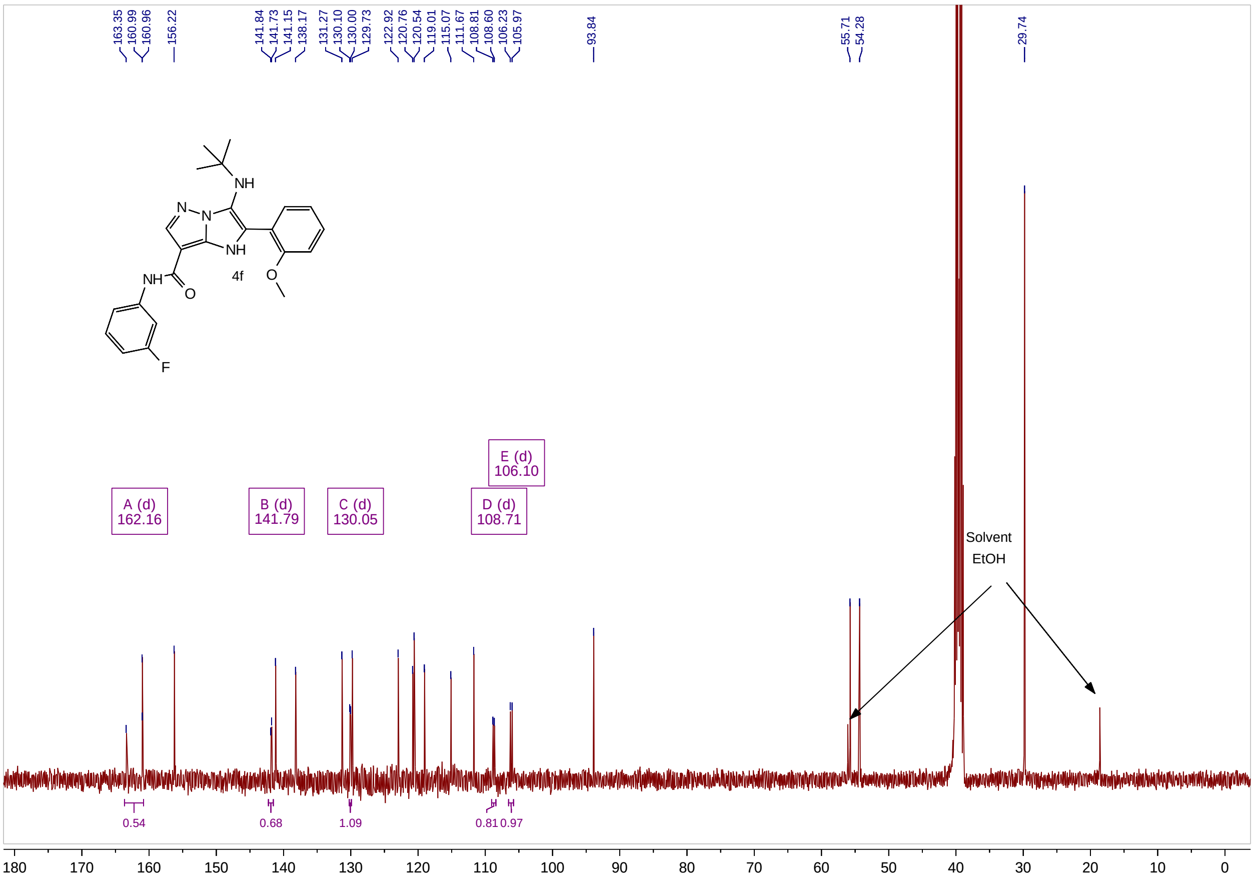
163.35 160.99 160.96 156.22
141.84 141.73 141.15 138.17 131.27 130.10 130.00 129.73 122.92 120.76 120.54 119.01 115.07 111.67 108.81 108.60 106.23 105.97
93.84
55.71 54.28
29.74
NH
N
N
NH
NH
O
O
F
4f
A (d) 162.16
B (d) 141.79
C (d) 130.05
D (d) 108.71
E (d) 106.10
Solvent
EtOH
0.54 0.68 1.09 0.81 0.97

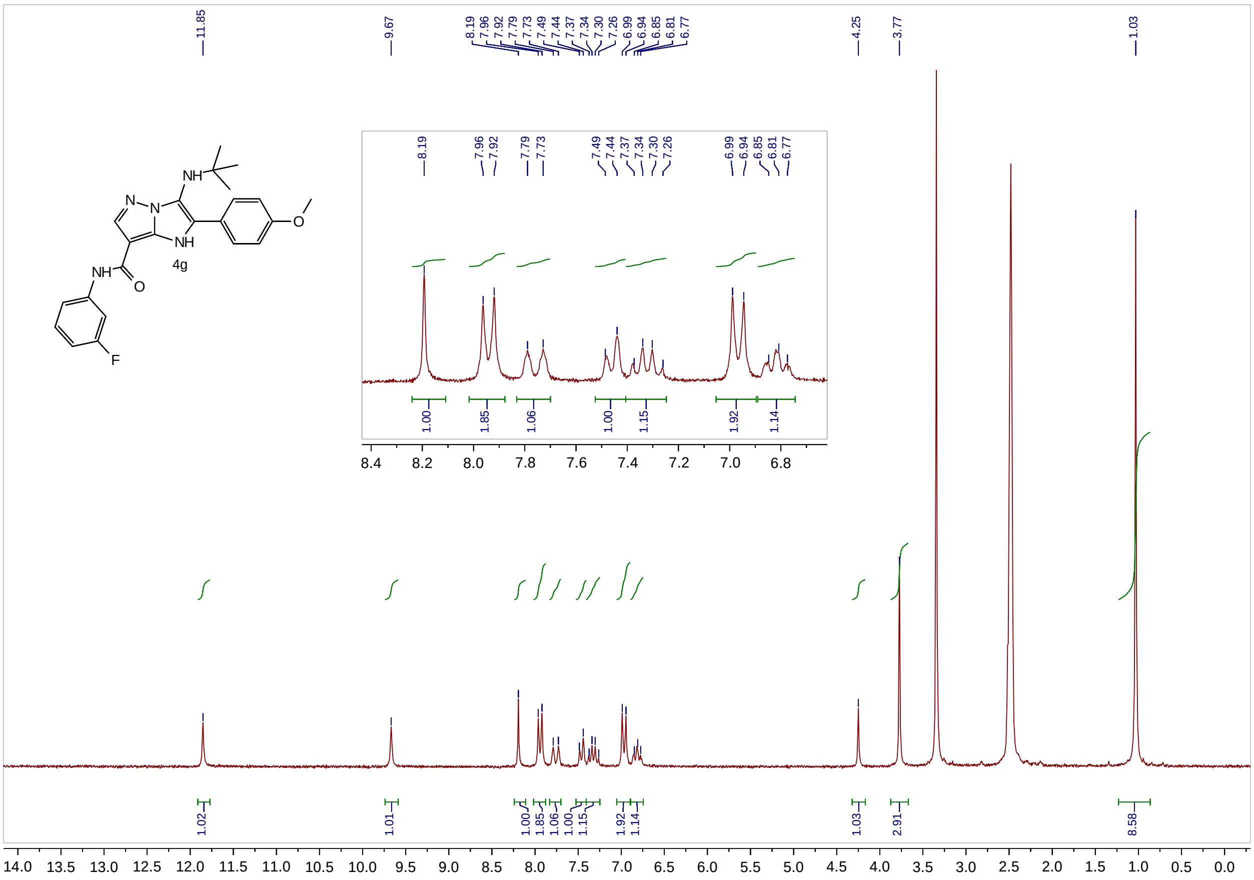
11.85
9.67
8.19 7.96 7.92 7.79 7.73 7.49 7.44 7.37 7.34 7.30 7.26 6.99 6.94 6.85 6.81 6.77
4.25
3.77
1.03
NH
N
N
NH
O
4g
NH
O
F
1.00 1.85 1.06 1.00 1.15 1.92 1.14
8.4 8.2 8.0 7.8 7.6 7.4 7.2 7.0 6.8
1.02 1.01 1.00 1.85 1.06 1.00 1.15 1.92 1.14 1.03 2.91 8.58
14.0 13.5 13.0 12.5 12.0 11.5 11.0 10.5 10.0 9.5 9.0 8.5 8.0 7.5 7.0 6.5 6.0 5.5 5.0 4.5 4.0 3.5 3.0 2.5 2.0 1.5 1.0 0.5 0.0

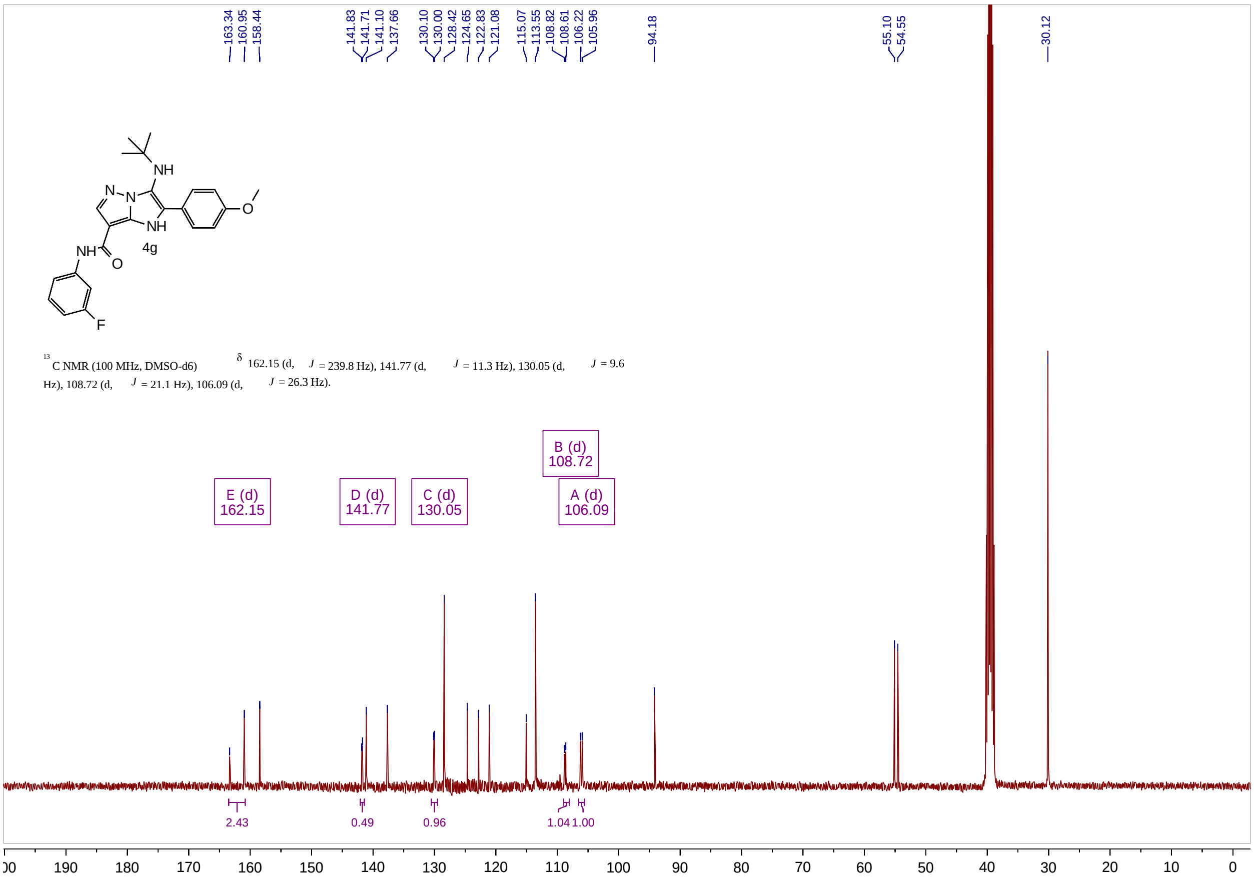
163.34 160.95 158.44 141.83 141.71 141.10 137.66 130.10 130.00 128.42 124.65 122.83 121.08 115.07 113.55 108.82 108.61 106.22 105.96 94.18 55.10 54.55 30.12
NH
N
N
NH
O
4g
NH
O
F
13C NMR (100 MHz, DMSO-d6) δ 162.15 (d, J = 239.8 Hz), 141.77 (d, J = 11.3 Hz), 130.05 (d, J = 9.6 Hz), 108.72 (d, J = 21.1 Hz), 106.09 (d, J = 26.3 Hz).
B (d) 108.72
E (d) 162.15
D (d) 141.77
C (d) 130.05
A (d) 106.09
2.43 0.49 0.96 1.04 1.00
00 190 180 170 160 150 140 130 120 110 100 90 80 70 60 50 40 30 20 10 0

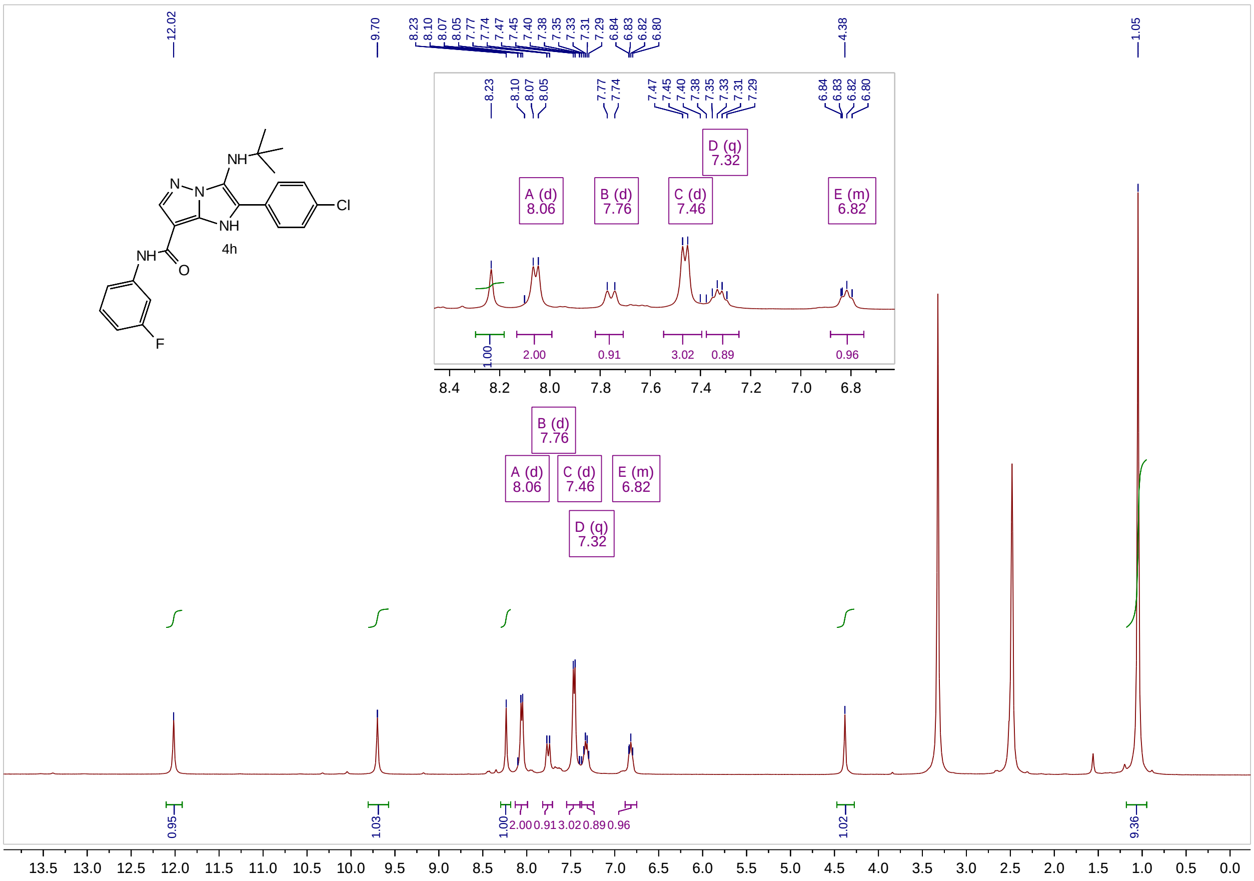

4h
A (d) 8.06
B (d) 7.76
C (d) 7.46
D (q) 7.32
E (m) 6.82

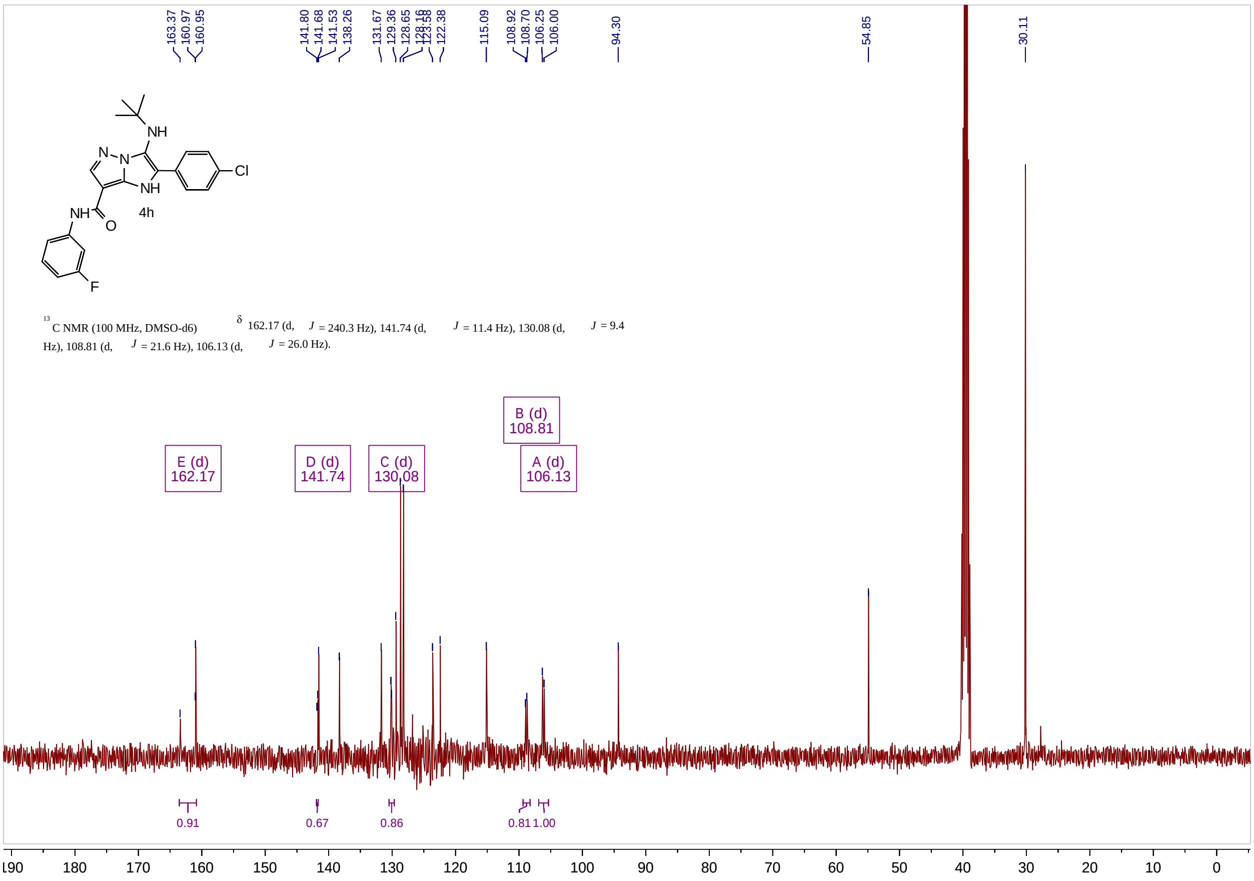

4h
^{13}C NMR (100 MHz, DMSO-d6) δ 162.17 (d, J = 240.3 Hz), 141.74 (d, J = 11.4 Hz), 130.08 (d, J = 9.4 Hz), 108.81 (d, J = 21.6 Hz), 106.13 (d, J = 26.0 Hz).
E (d) 162.17
D (d) 141.74
C (d) 130.08
B (d) 108.81
A (d) 106.13

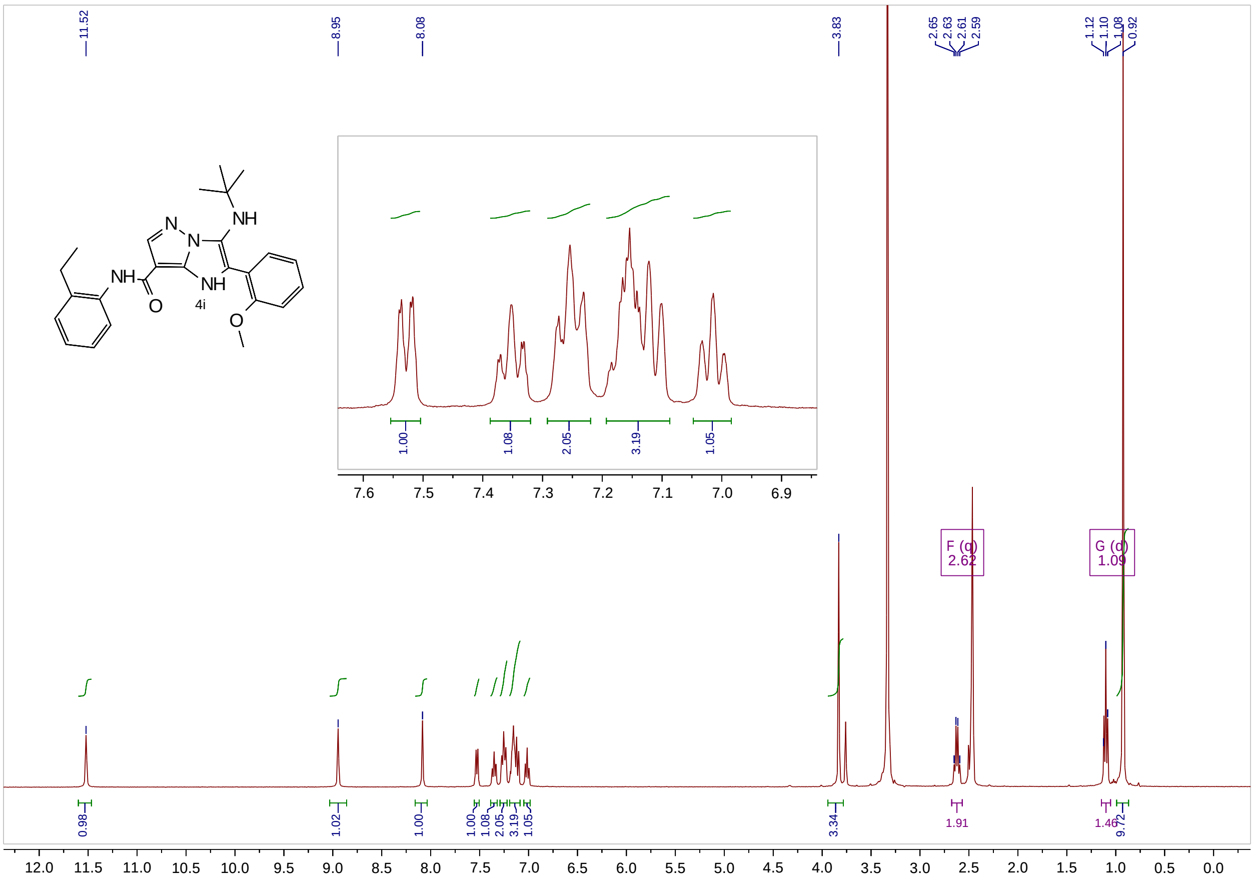
11.52
8.95
8.08
3.83
2.65 2.63 2.61 2.59
1.12 1.10 1.08 0.92
NH
N
N
NH
NH
O
4i
O
1.00
1.08
2.05
3.19
1.05
7.6 7.5 7.4 7.3 7.2 7.1 7.0 6.9
F (q)
2.62
G (d)
1.09
0.98
1.02
1.00
1.00 1.08 2.05 3.19 1.05
3.34
1.91
1.46
9.72
12.0 11.5 11.0 10.5 10.0 9.5 9.0 8.5 8.0 7.5 7.0 6.5 6.0 5.5 5.0 4.5 4.0 3.5 3.0 2.5 2.0 1.5 1.0 0.5 0.0

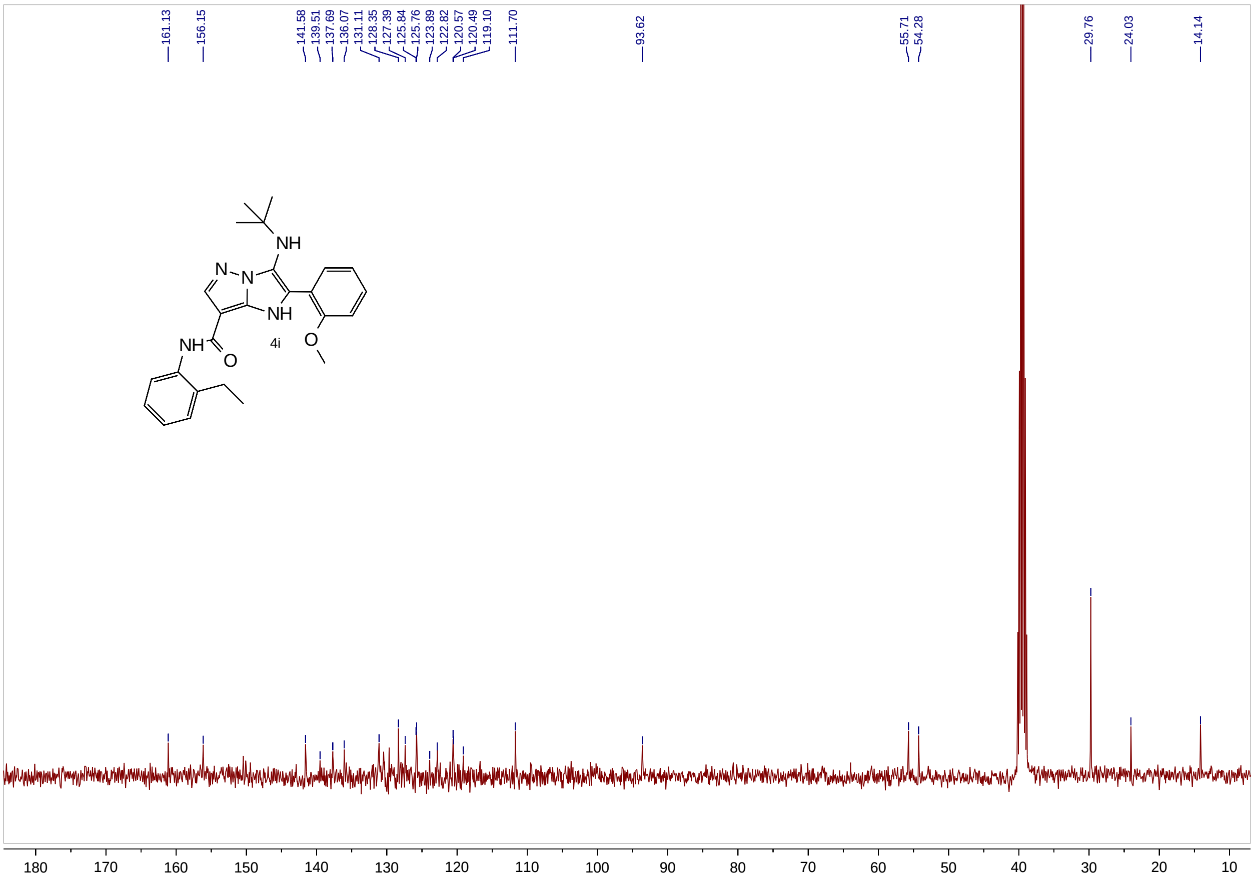
161.13
156.15
141.58 139.51 137.69 136.07 131.11 128.35 127.39 125.84 125.76 123.89 122.82 120.57 120.49 119.10
111.70
93.62
55.71 54.28
29.76
24.03
14.14
NH
N
N
NH
NH
O
4i
O
180 170 160 150 140 130 120 110 100 90 80 70 60 50 40 30 20 10

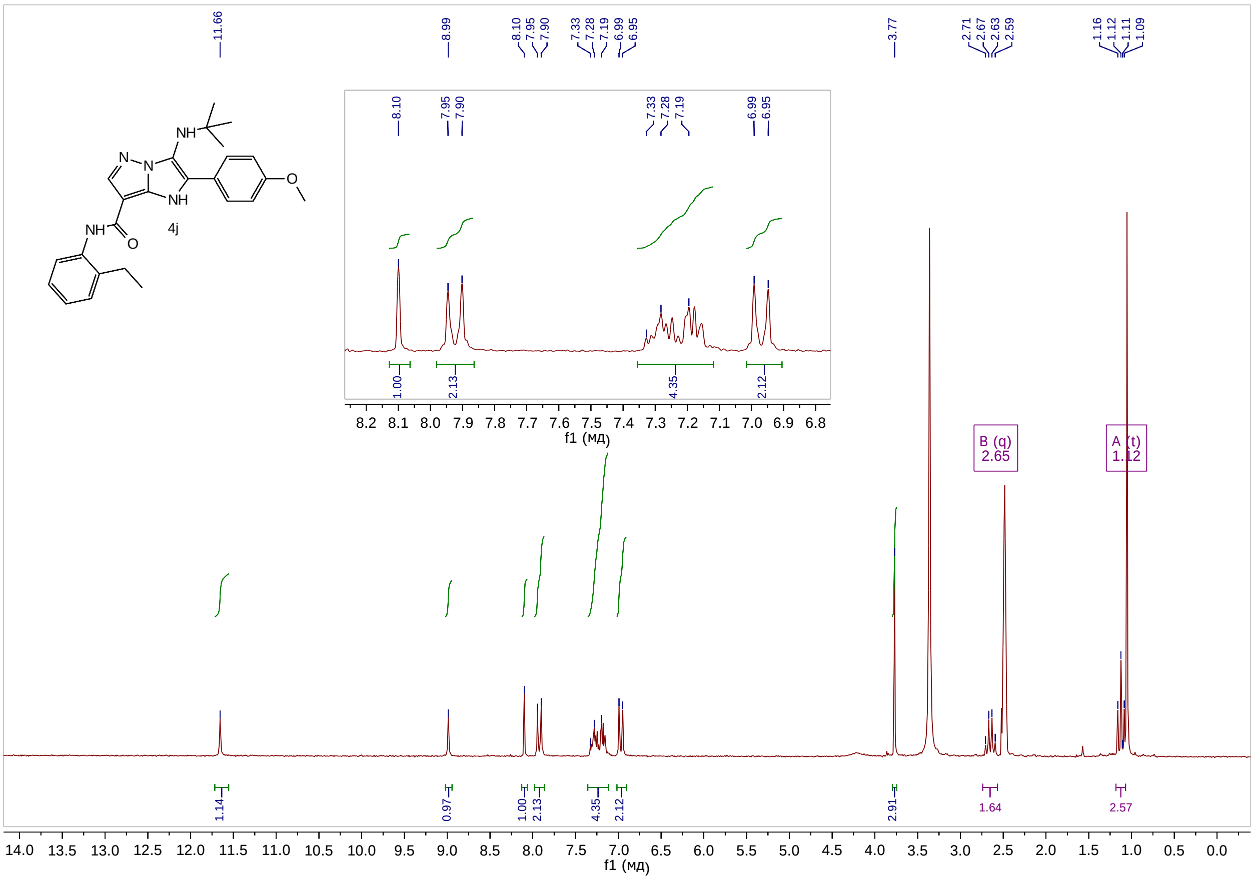
11.66
8.99
8.10
7.95
7.90
7.33
7.28
7.19
6.99
6.95
3.77
2.71
2.67
2.63
2.59
1.16
1.12
1.11
1.09
NH
N
N
NH
O
NH
O
4j
B (q)
2.65
A (t)
1.12
1.00
2.13
4.35
2.12
f1 (мд)
1.14
0.97
1.00
2.13
4.35
2.12
2.91
1.64
2.57
f1 (мд)

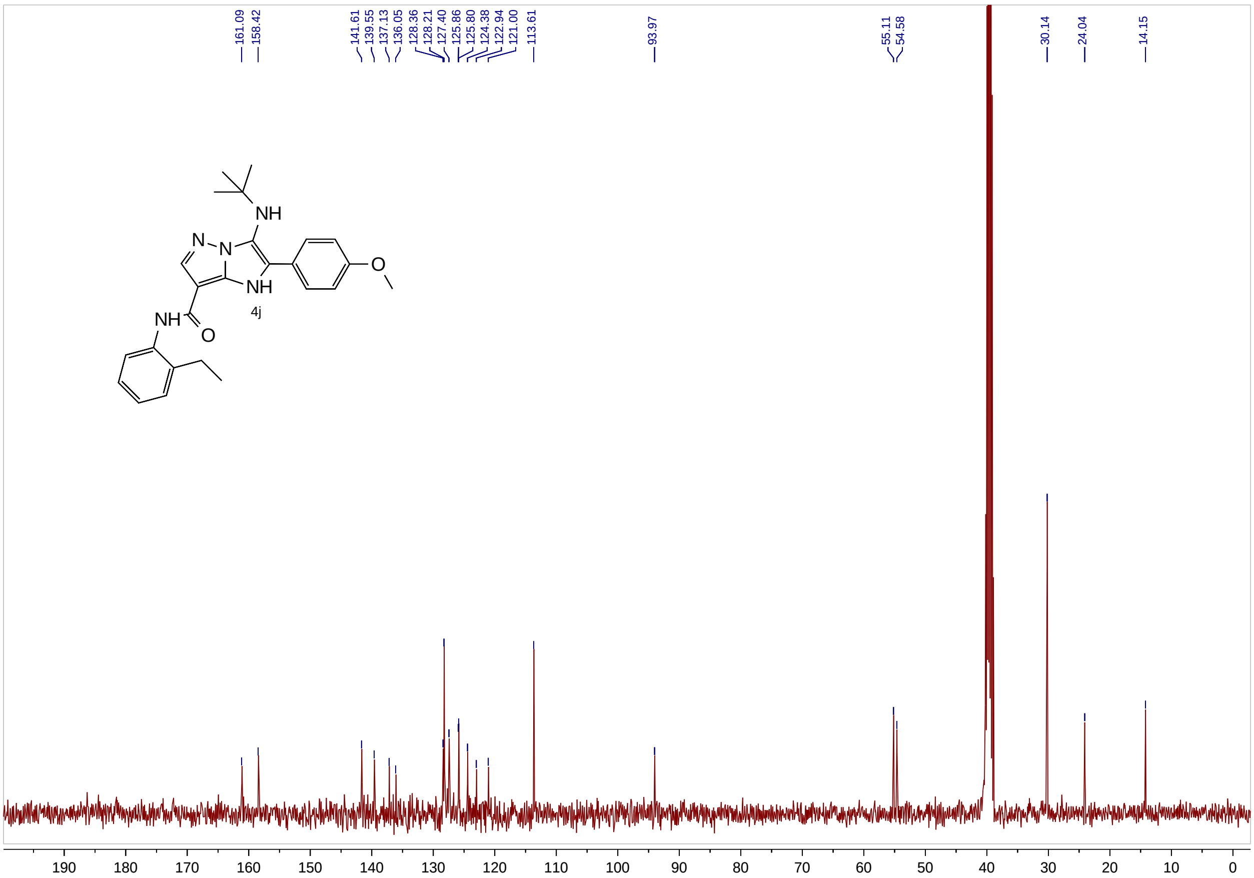
161.09
158.42
141.61
139.55
137.13
136.05
128.36
128.21
127.40
125.86
125.80
124.38
122.94
121.00
113.61
93.97
55.11
54.58
30.14
24.04
14.15
NH
N
N
NH
O
NH
O
4j

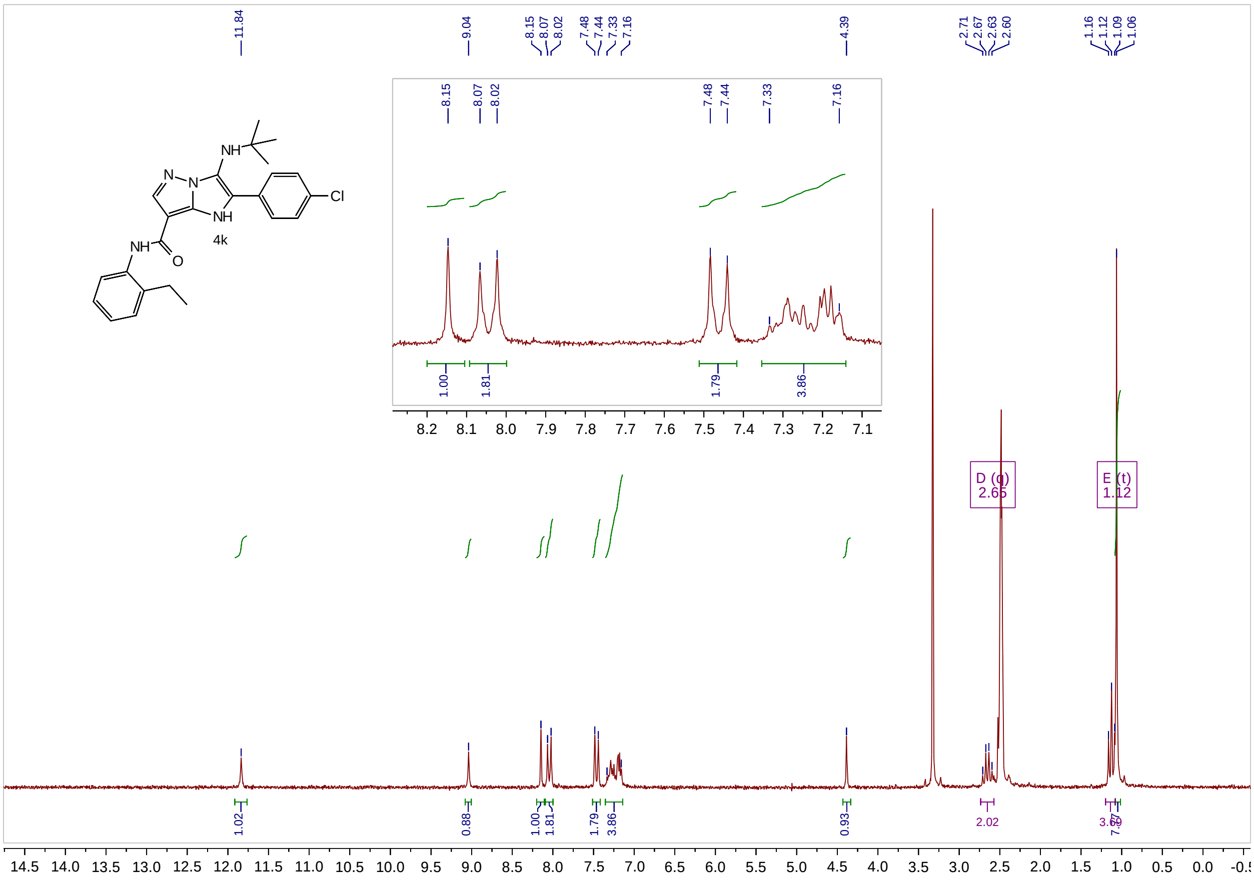
11.84
9.04
8.15
8.07
8.02
7.48
7.44
7.33
7.16
4.39
2.71
2.67
2.63
2.60
1.16
1.12
1.09
1.06
NH
N
N
NH
Cl
NH
O
4k
D (q)
2.65
E (t)
1.12
1.02
0.88
1.00
1.81
1.79
3.86
0.93
2.02
14.5 14.0 13.5 13.0 12.5 12.0 11.5 11.0 10.5 10.0 9.5 9.0 8.5 8.0 7.5 7.0 6.5 6.0 5.5 5.0 4.5 4.0 3.5 3.0 2.5 2.0 1.5 1.0 0.5 0.0

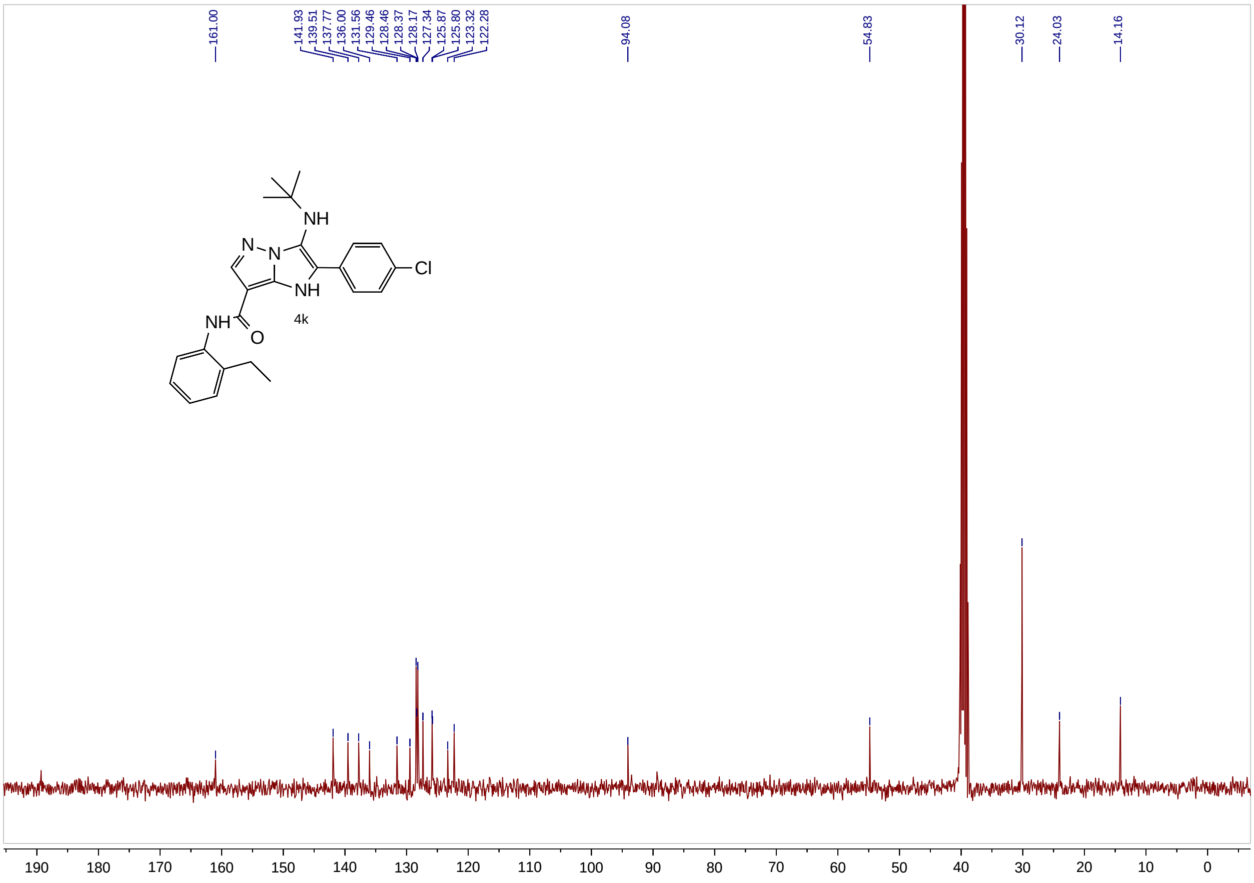
161.00
141.93
139.51
137.77
136.00
131.56
129.46
128.46
128.37
128.17
127.34
125.87
125.80
123.32
122.28
94.08
54.83
30.12
24.03
14.16
NH
N
N
NH
Cl
NH
O
4k
190 180 170 160 150 140 130 120 110 100 90 80 70 60 50 40 30 20 10 0

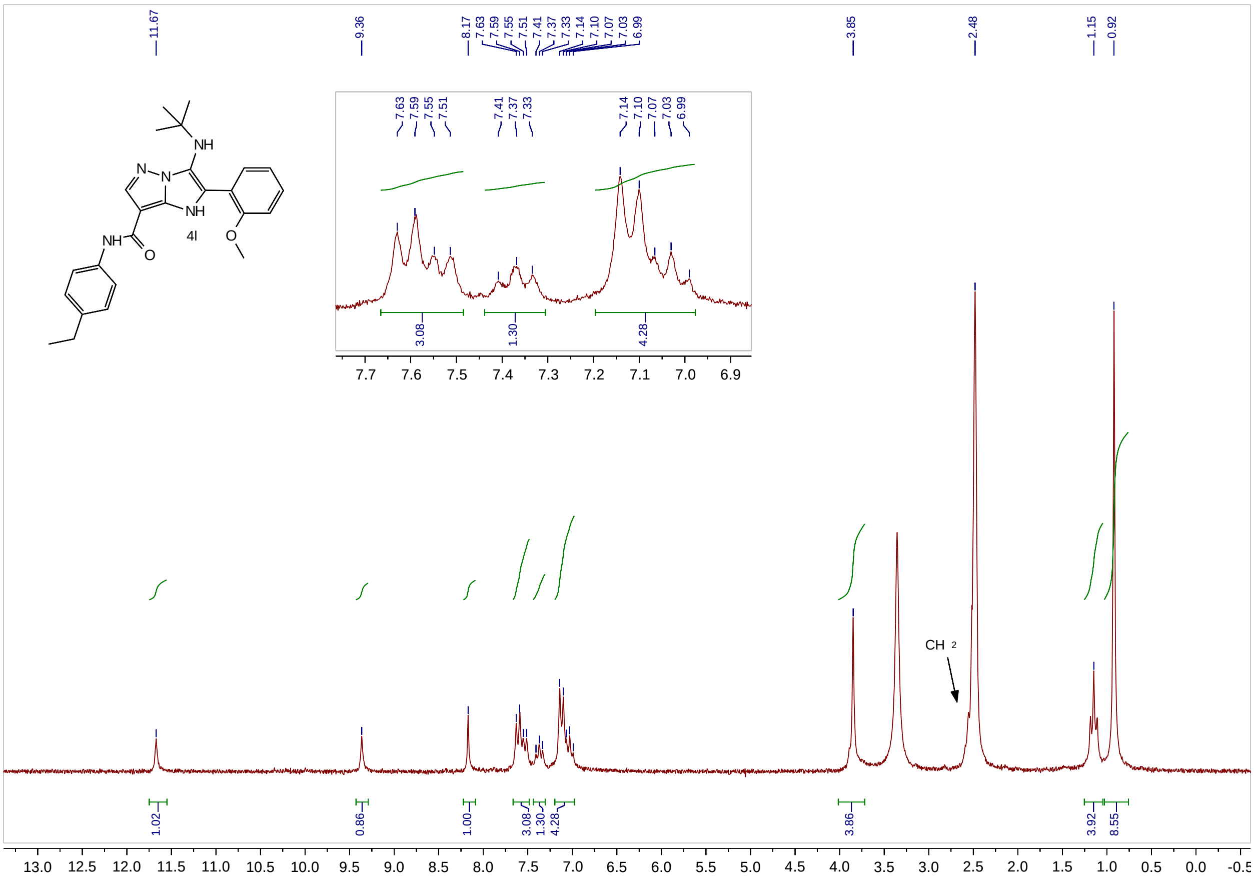
11.67
9.36
8.17
7.63
7.59
7.55
7.51
7.41
7.37
7.33
7.14
7.10
7.07
7.03
6.99
3.85
2.48
1.15
0.92
NH
N
N
NH
NH
O
O
4l
CH 2
3.08
1.30
4.28
1.02
0.86
1.00
3.86
3.92
8.55
13.0 12.5 12.0 11.5 11.0 10.5 10.0 9.5 9.0 8.5 8.0 7.5 7.0 6.5 6.0 5.5 5.0 4.5 4.0 3.5 3.0 2.5 2.0 1.5 1.0 0.5 0.0 -0.5
7.7 7.6 7.5 7.4 7.3 7.2 7.1 7.0 6.9

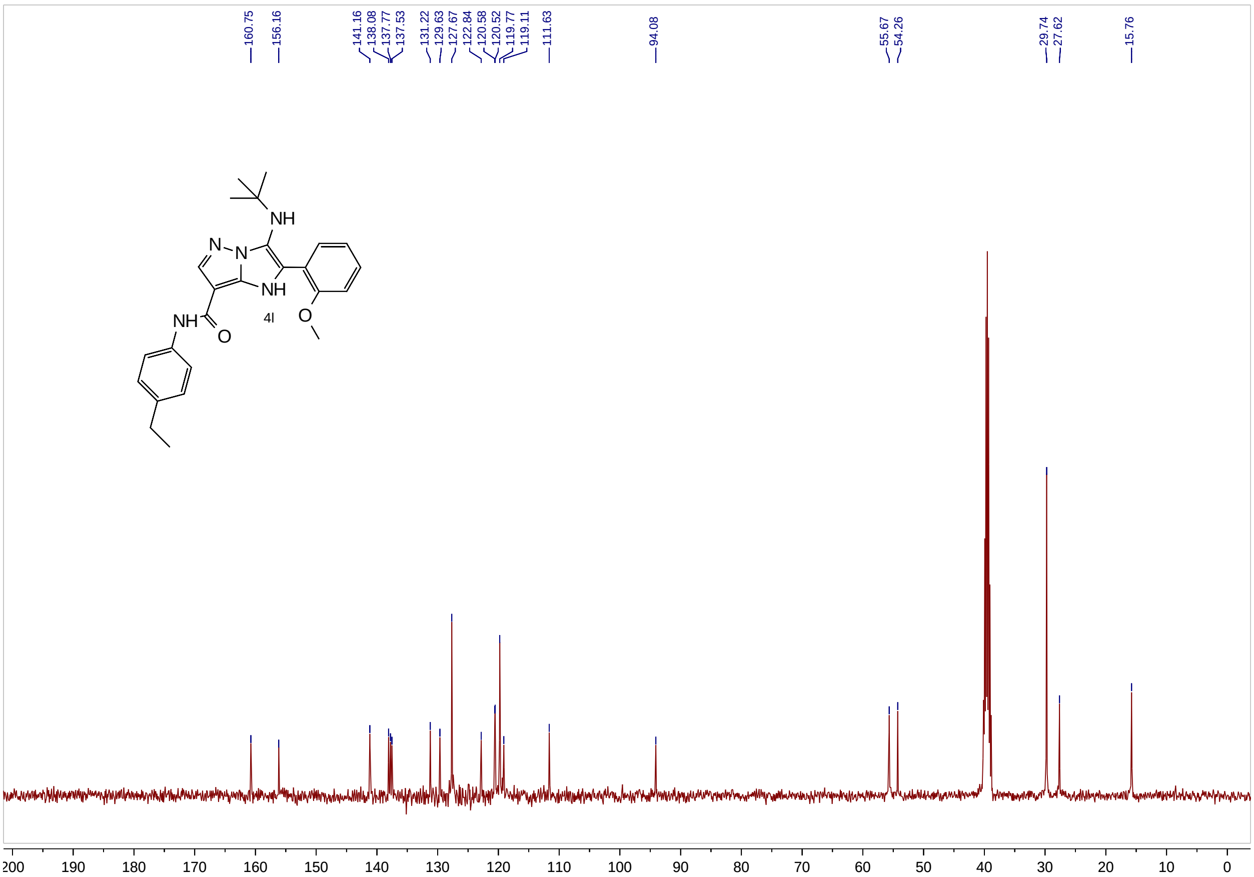
160.75
156.16
141.16
138.08
137.77
137.53
131.22
129.63
127.67
122.84
120.58
120.52
119.77
119.11
111.63
94.08
55.67
54.26
29.74
27.62
15.76
NH
N
N
NH
NH
O
O
4l
200 190 180 170 160 150 140 130 120 110 100 90 80 70 60 50 40 30 20 10 0

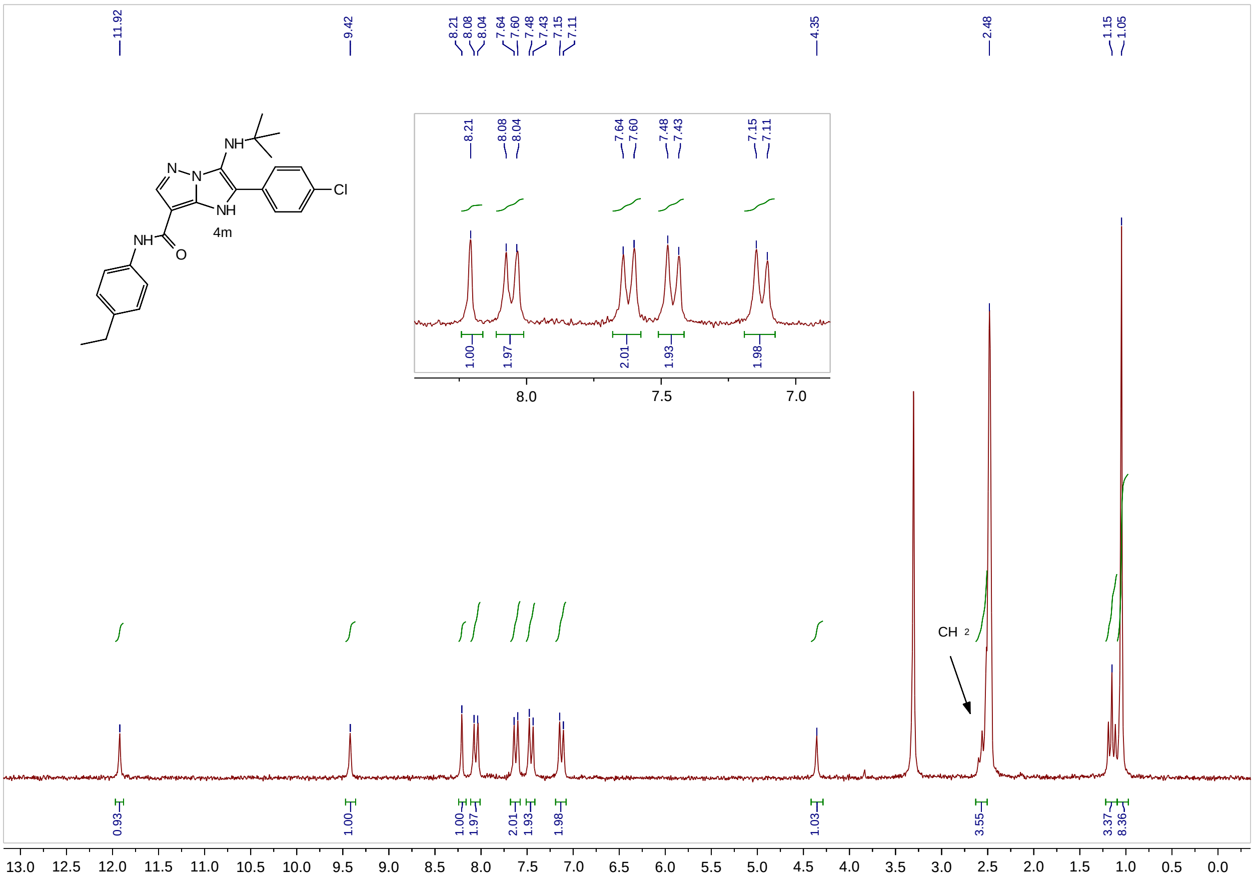
11.92
9.42
8.21
8.08
8.04
7.64
7.60
7.48
7.43
7.15
7.11
4.35
2.48
1.15
1.05
NH
N
N
Cl
NH
4m
NH
O
CH 2
0.93
1.00
1.00
1.97
2.01
1.93
1.98
1.03
3.55
3.37
8.36
13.0 12.5 12.0 11.5 11.0 10.5 10.0 9.5 9.0 8.5 8.0 7.5 7.0 6.5 6.0 5.5 5.0 4.5 4.0 3.5 3.0 2.5 2.0 1.5 1.0 0.5 0.0

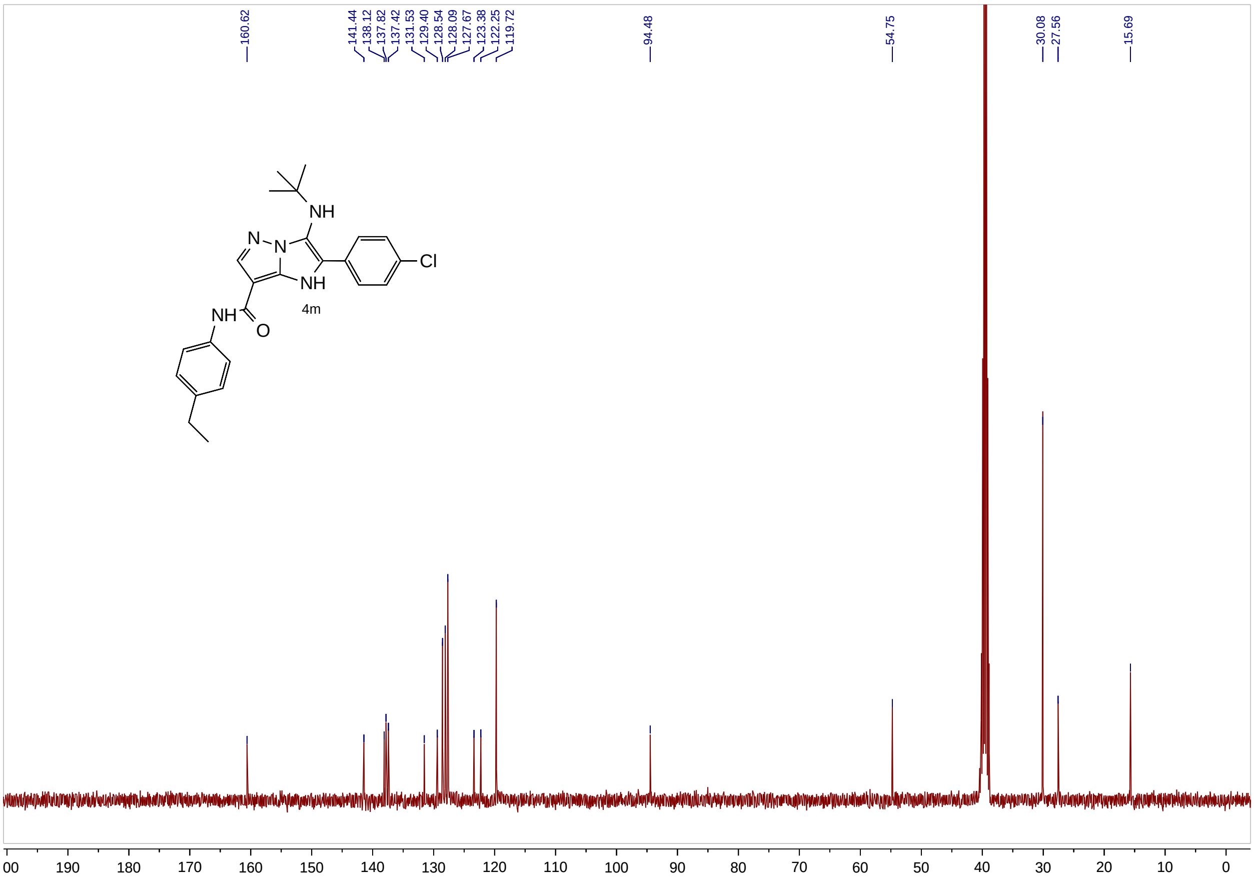
160.62
141.44
138.12
137.82
137.42
131.53
129.40
128.54
128.09
127.67
123.38
122.25
119.72
94.48
54.75
30.08
27.56
15.69
NH
N
N
Cl
NH
4m
NH
O
00 190 180 170 160 150 140 130 120 110 100 90 80 70 60 50 40 30 20 10 0

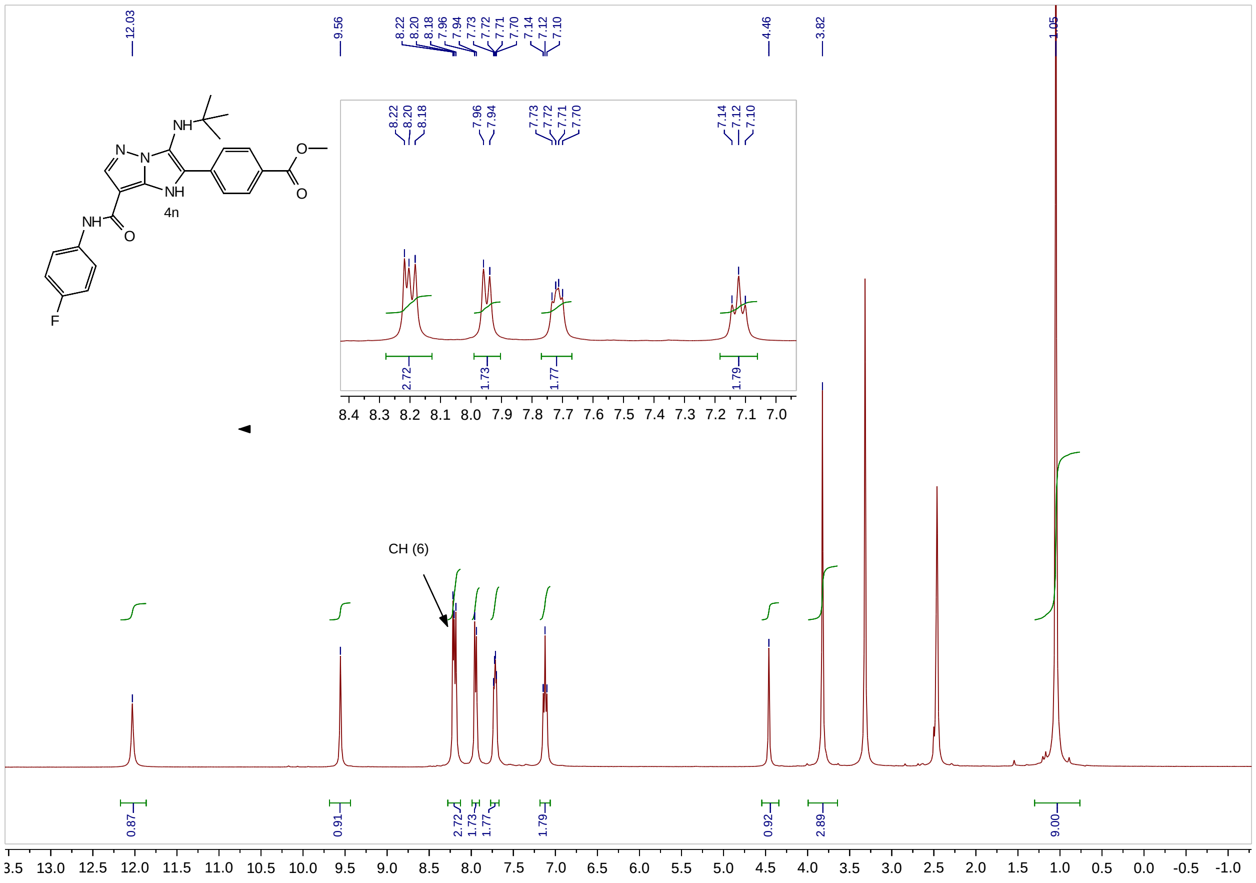
4n
CH (6)

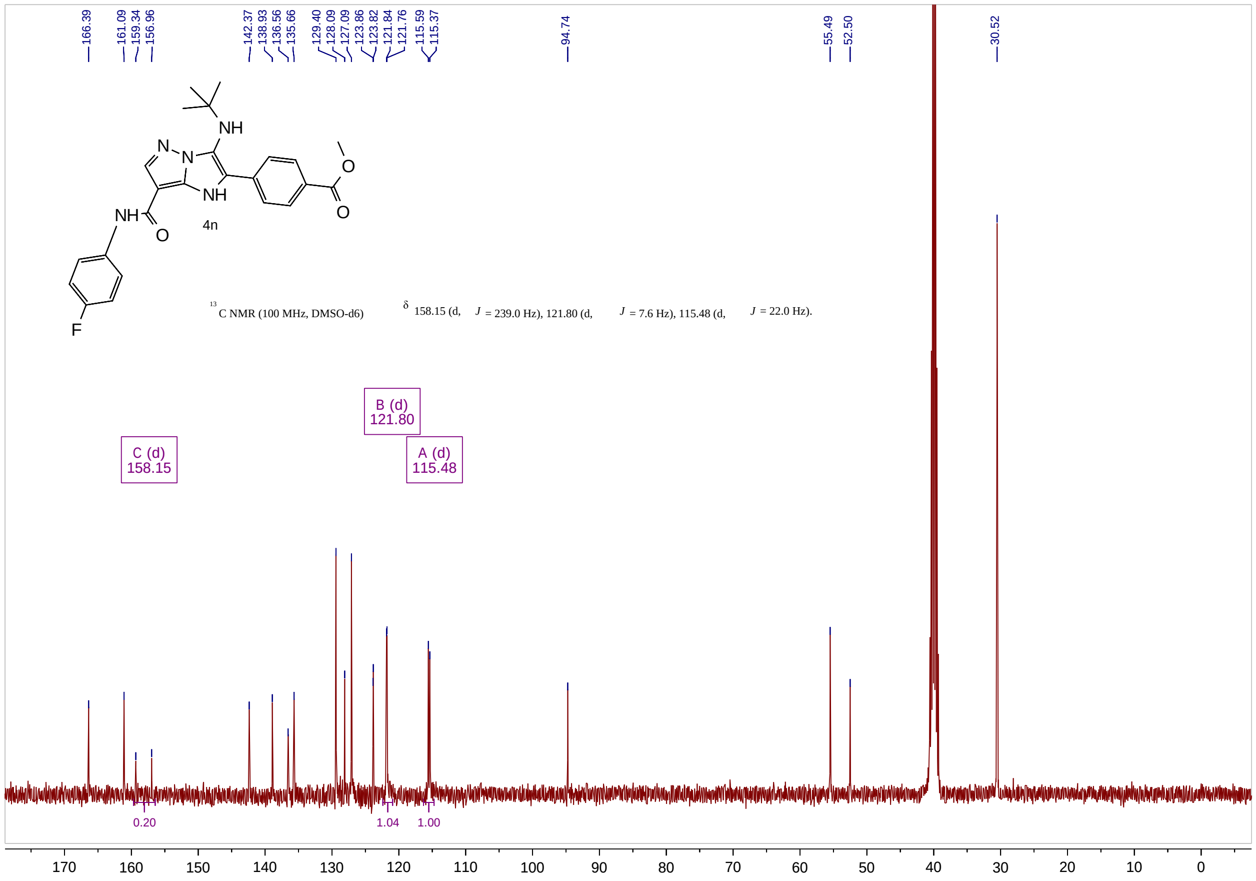
4n
13C NMR (100 MHz, DMSO-d6) δ 158.15 (d, J = 239.0 Hz), 121.80 (d, J = 7.6 Hz), 115.48 (d, J = 22.0 Hz).
B (d)
121.80
C (d)
158.15
A (d)
115.48

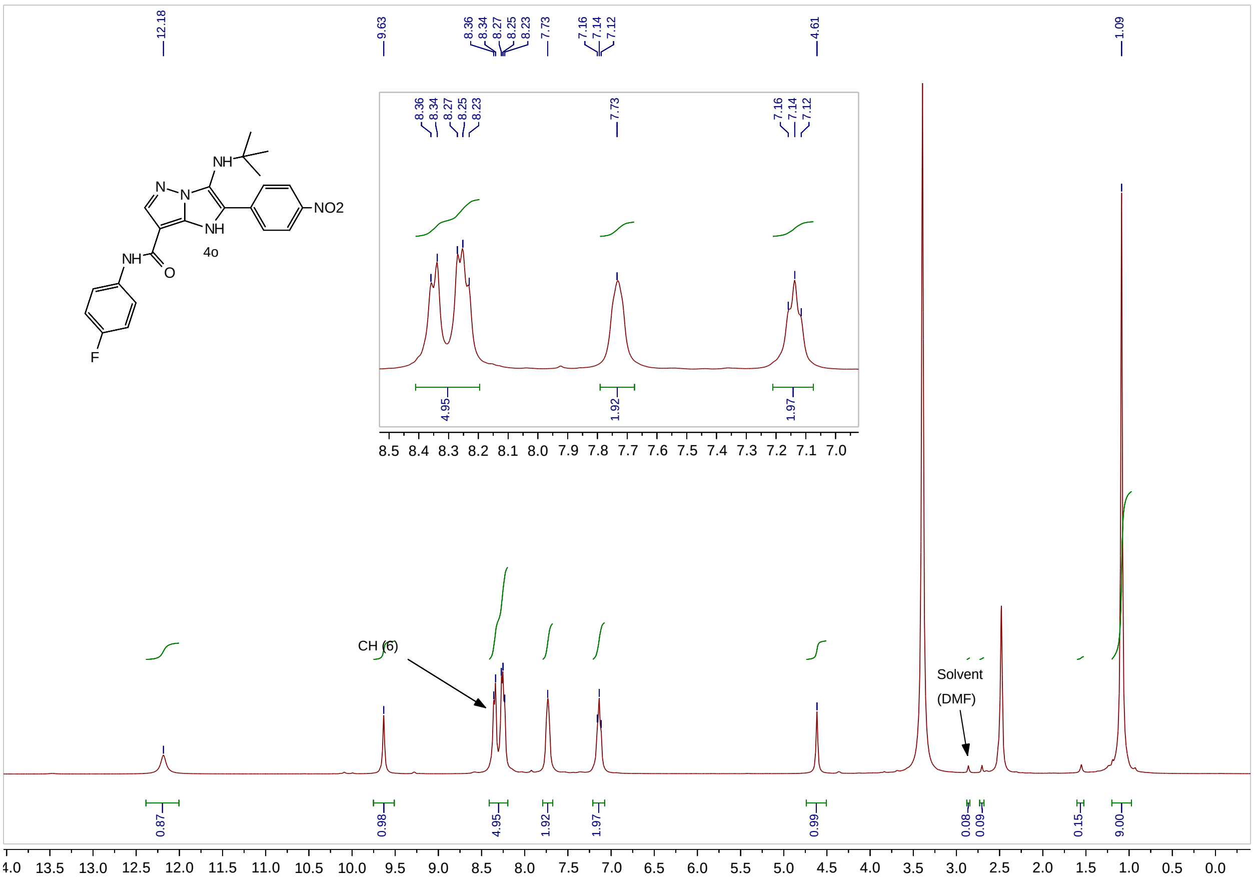
12.18
9.63
8.36
8.34
8.27
8.25
8.23
7.73
7.16
7.14
7.12
4.61
1.09
NH
N
N
NO2
NH
NH
O
4o
F
CH (6)
Solvent
(DMF)
4.95
1.92
1.97
0.87
0.98
0.99
0.08
0.09
0.15
9.00

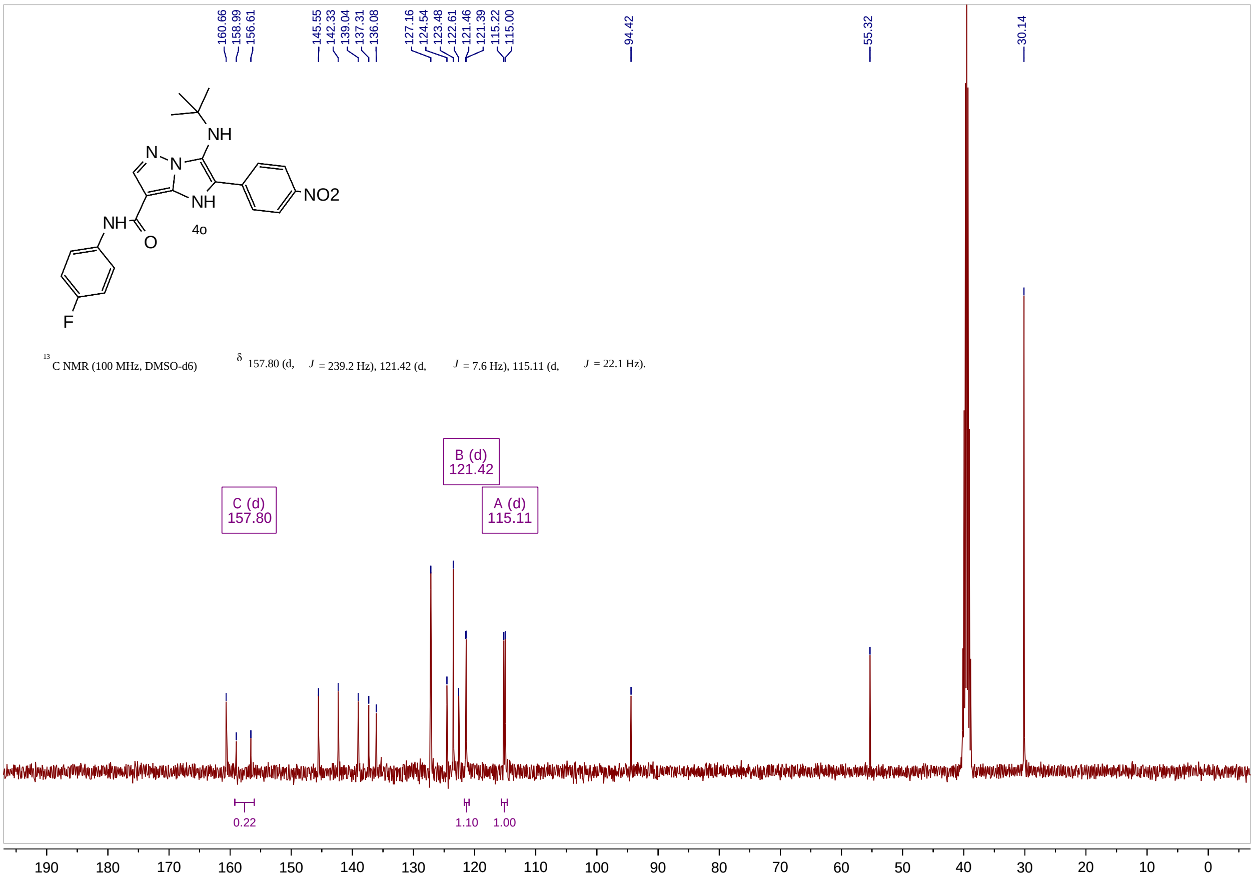
160.66
158.99
156.61
145.55
142.33
139.04
137.31
136.08
127.16
124.54
123.48
122.61
121.46
121.39
115.22
115.00
94.42
55.32
30.14
NH
N
N
NO2
NH
NH
O
4o
F
13C NMR (100 MHz, DMSO-d6) δ 157.80 (d, J = 239.2 Hz), 121.42 (d, J = 7.6 Hz), 115.11 (d, J = 22.1 Hz).
B (d)
121.42
C (d)
157.80
A (d)
115.11
0.22
1.10
1.00

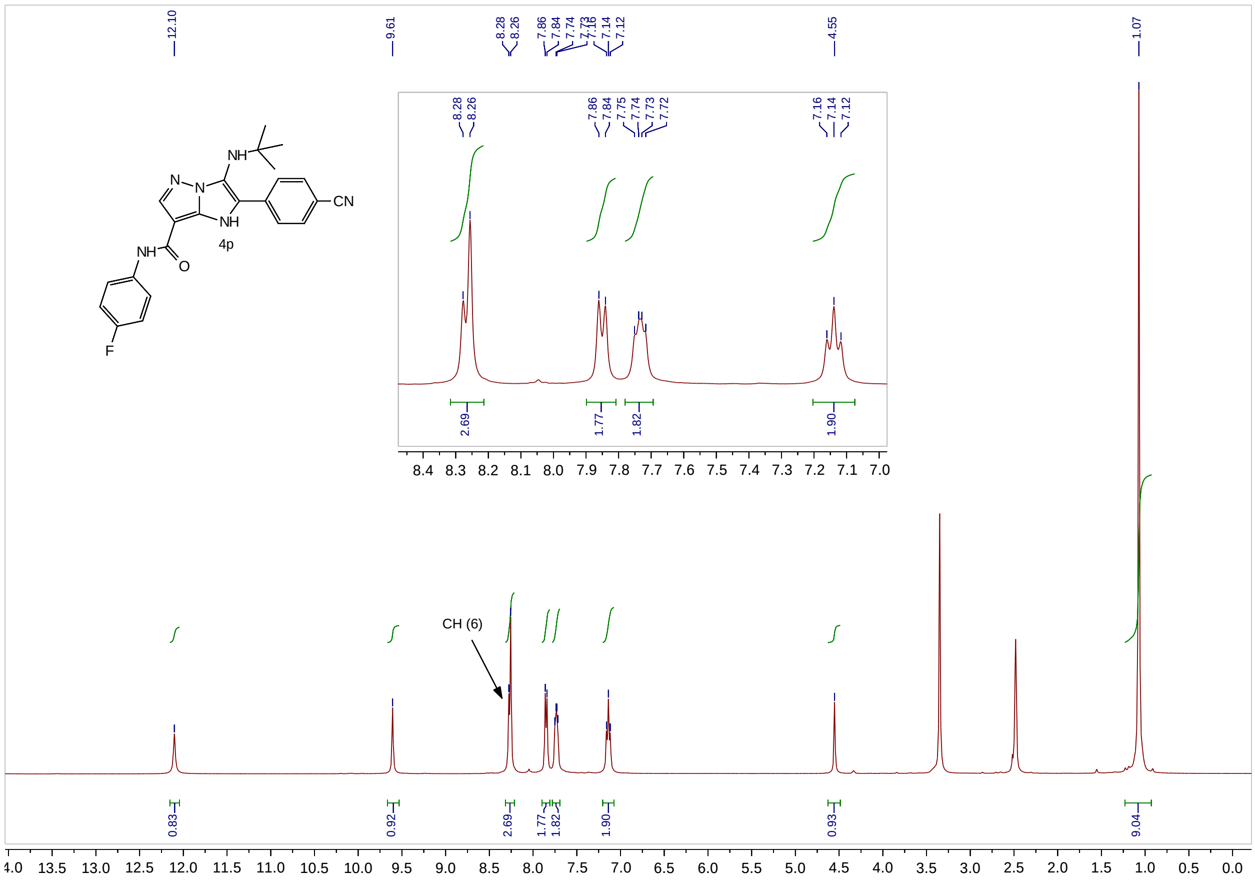
4p
CH (6)
12.10
9.61
8.28
8.26
7.86
7.84
7.74
7.73
7.16
7.14
7.12
4.55
1.07
0.83
0.92
2.69
1.77
1.82
1.90
0.93
9.04

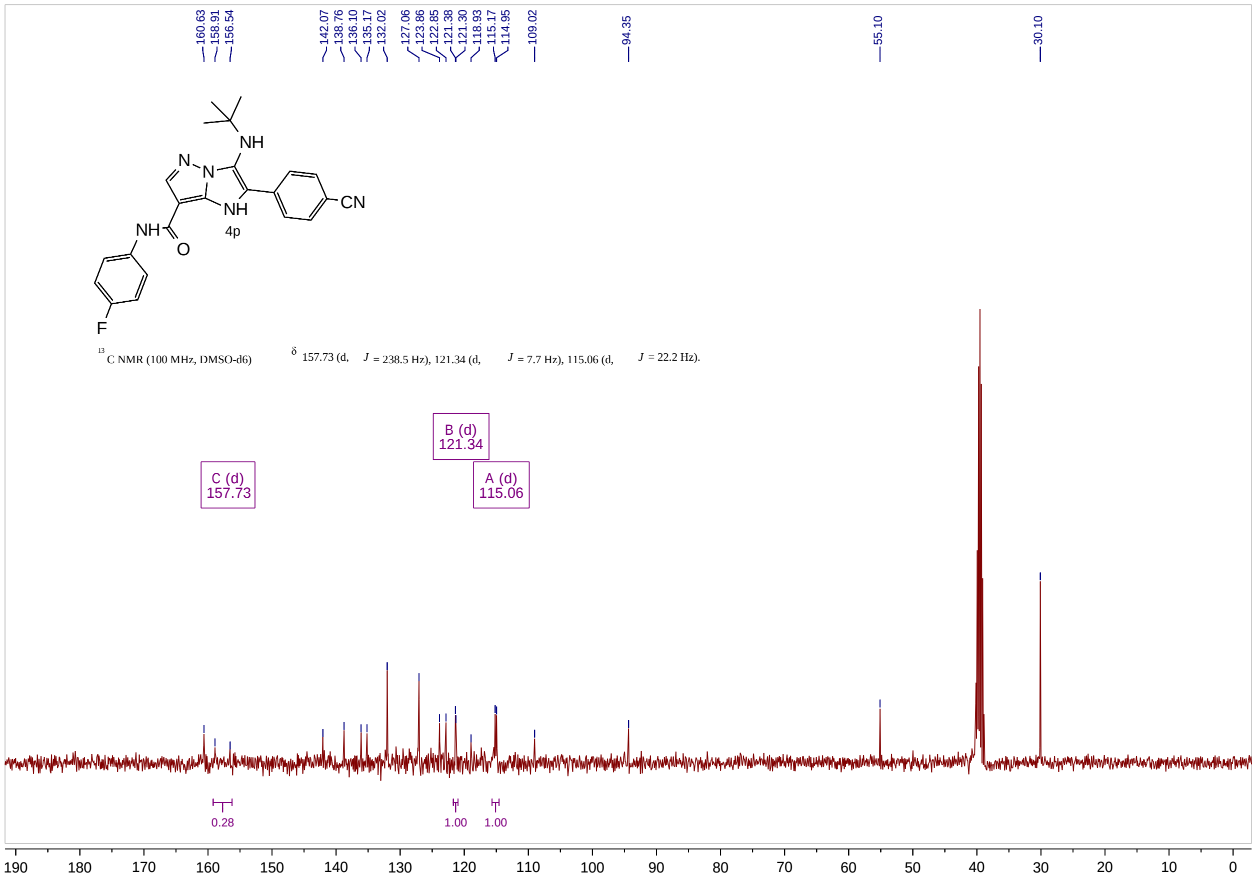
4p
13C NMR (100 MHz, DMSO-d6) δ 157.73 (d, J = 238.5 Hz), 121.34 (d, J = 7.7 Hz), 115.06 (d, J = 22.2 Hz).
C (d)
157.73
B (d)
121.34
A (d)
115.06
160.63
158.91
156.54
142.07
138.76
136.10
135.17
132.02
127.06
123.86
122.85
121.38
121.30
118.93
115.17
114.95
109.02
94.35
55.10
30.10
0.28
1.00
1.00

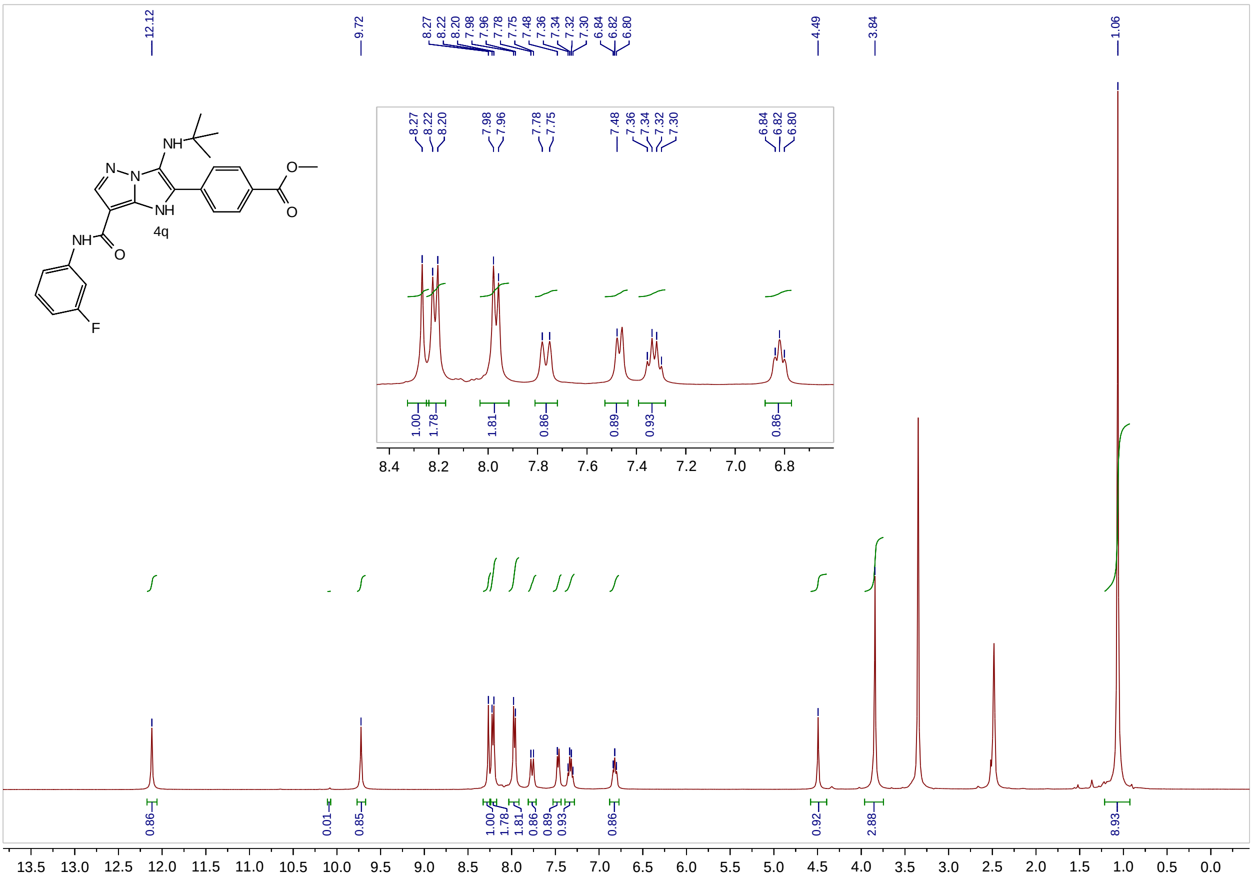
12.12
9.72
8.27 8.22 8.20 7.98 7.96 7.78 7.75 7.48 7.36 7.34 7.32 7.30 6.84 6.82 6.80
4.49
3.84
1.06
4q
NH
N
O
F
0.86 0.01 0.85 1.00 1.78 1.81 0.86 0.89 0.93 0.86 0.92 2.88 8.93

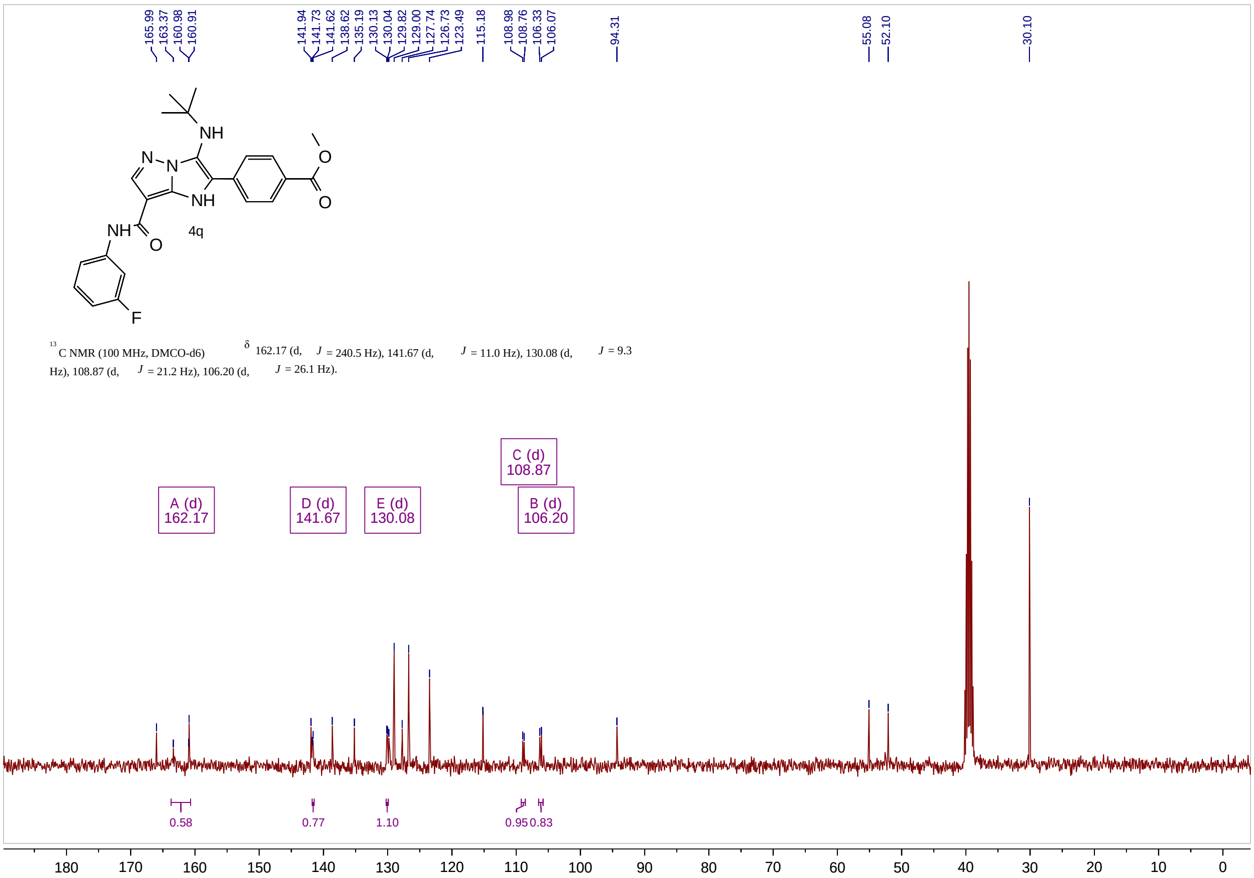
165.99 163.37 160.98 160.91
141.94 141.73 141.62 138.62 135.19 130.13 130.04 129.82 129.00 127.74 126.73 123.49
115.18
108.98 108.76 106.33 106.07
94.31
55.08 52.10
30.10
4q
13C NMR (100 MHz, DMCO-d6) δ 162.17 (d, J = 240.5 Hz), 141.67 (d, J = 11.0 Hz), 130.08 (d, J = 9.3 Hz), 108.87 (d, J = 21.2 Hz), 106.20 (d, J = 26.1 Hz).
A (d) 162.17
D (d) 141.67
E (d) 130.08
C (d) 108.87
B (d) 106.20
0.58 0.77 1.10 0.95 0.83

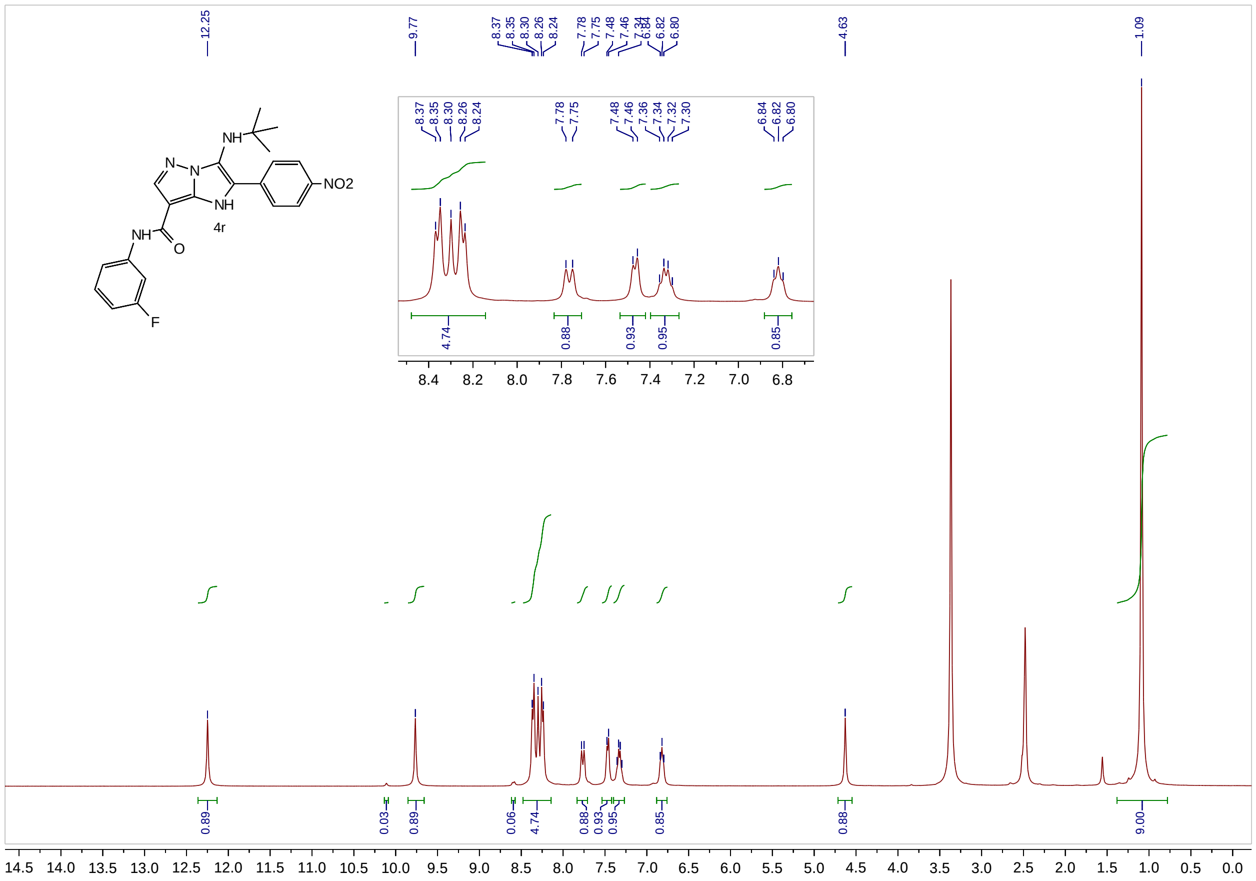
4r
NO2
12.25
9.77
4.63
1.09

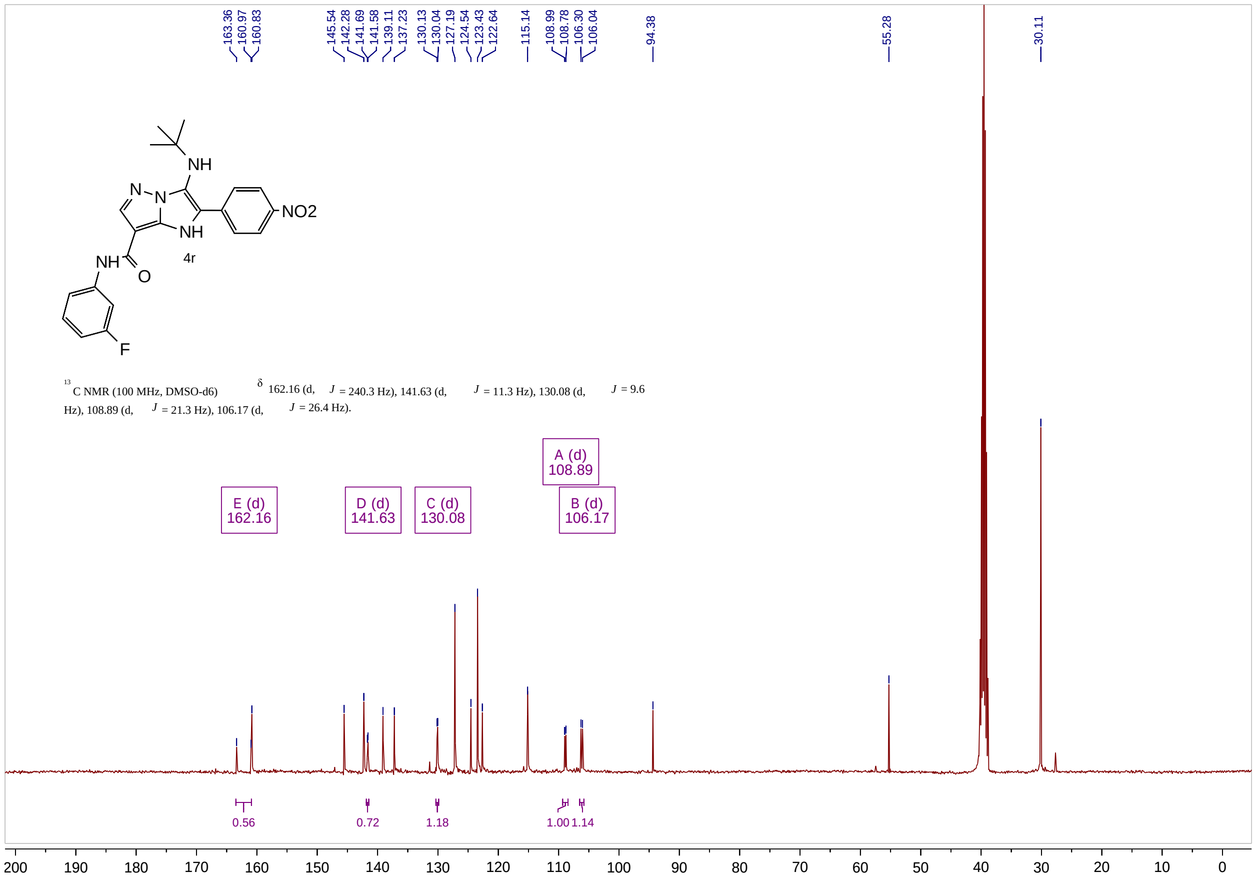
4r
NO2
13C NMR (100 MHz, DMSO-d6) δ 162.16 (d, J = 240.3 Hz), 141.63 (d, J = 11.3 Hz), 130.08 (d, J = 9.6 Hz), 108.89 (d, J = 21.3 Hz), 106.17 (d, J = 26.4 Hz).
A (d) 108.89
B (d) 106.17
C (d) 130.08
D (d) 141.63
E (d) 162.16

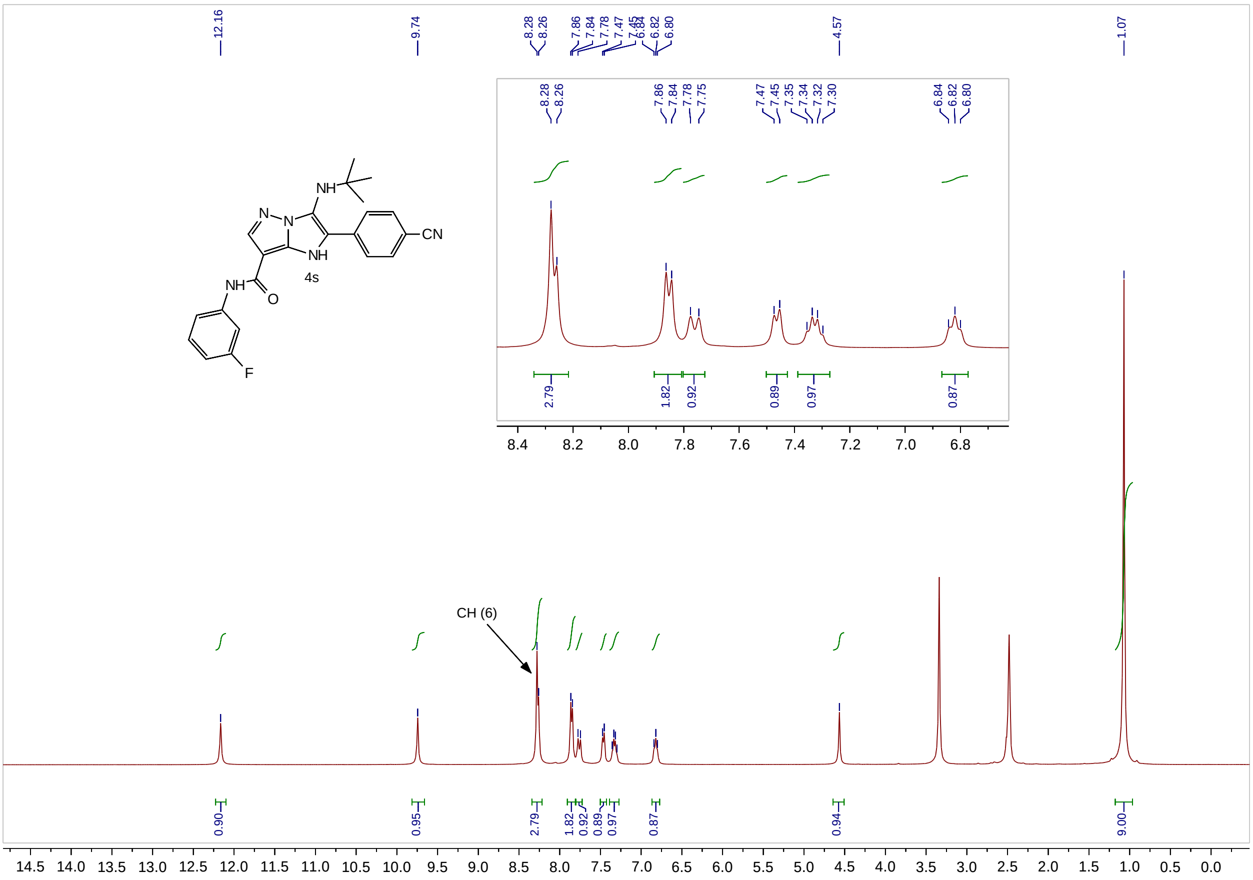
12.16
9.74
8.28
8.26
7.86
7.84
7.78
7.47
7.45
6.84
6.82
6.80
4.57
1.07
NH
N
N
NH
CN
NH
O
4s
F
CH (6)
0.90
0.95
2.79
1.82
0.92
0.89
0.97
0.87
0.94
9.00
7.75
7.35
7.34
7.32
7.30
14.5 14.0 13.5 13.0 12.5 12.0 11.5 11.0 10.5 10.0 9.5 9.0 8.5 8.0 7.5 7.0 6.5 6.0 5.5 5.0 4.5 4.0 3.5 3.0 2.5 2.0 1.5 1.0 0.5 0.0
8.4 8.2 8.0 7.8 7.6 7.4 7.2 7.0 6.8

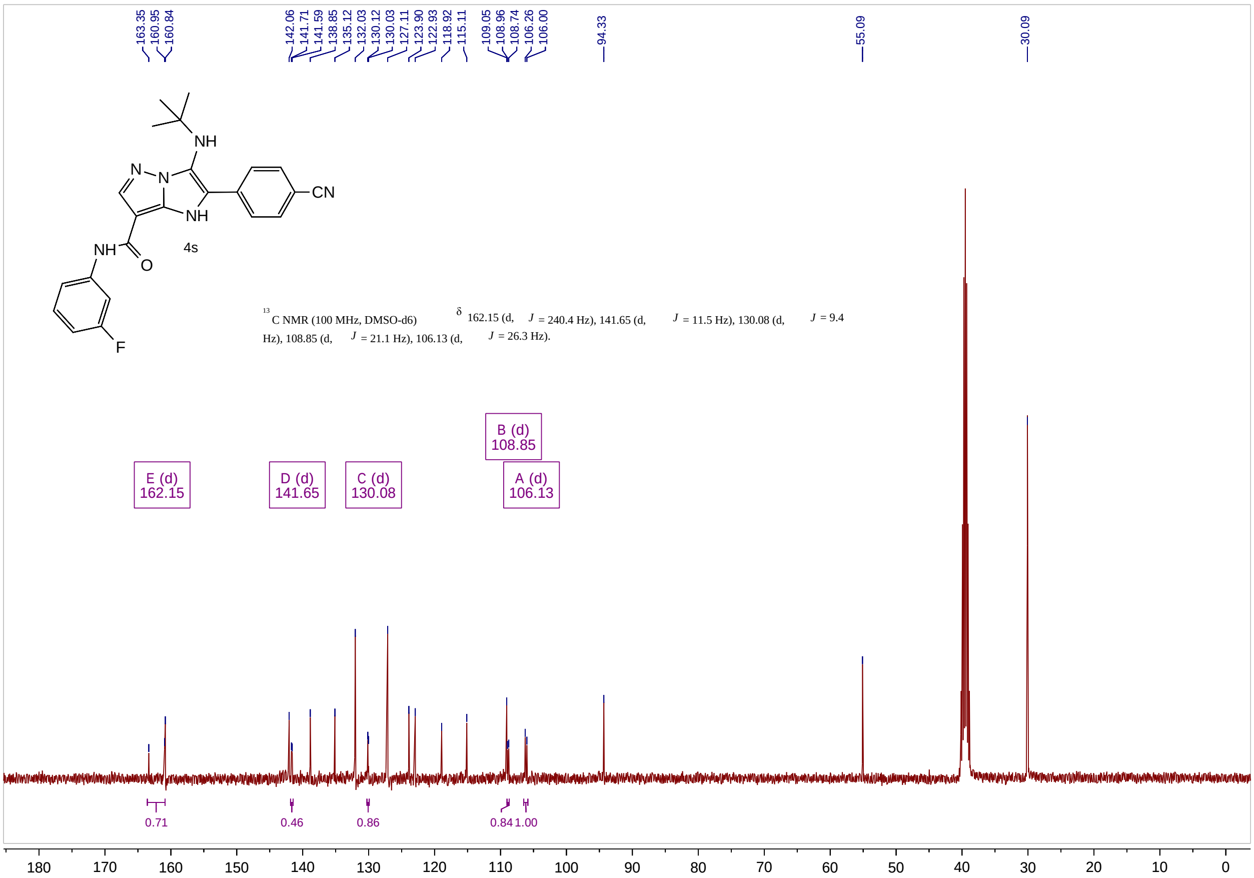
163.35
160.95
160.84
142.06
141.71
141.59
138.85
135.12
132.03
130.12
130.03
127.11
123.90
122.93
118.92
115.11
109.05
108.96
108.74
106.26
106.00
94.33
55.09
30.09
NH
N
N
NH
CN
NH
O
4s
F
13C NMR (100 MHz, DMSO-d6) δ 162.15 (d, J = 240.4 Hz), 141.65 (d, J = 11.5 Hz), 130.08 (d, J = 9.4 Hz), 108.85 (d, J = 21.1 Hz), 106.13 (d, J = 26.3 Hz).
B (d) 108.85
E (d) 162.15
D (d) 141.65
C (d) 130.08
A (d) 106.13
0.71
0.46
0.86
0.84
1.00
180 170 160 150 140 130 120 110 100 90 80 70 60 50 40 30 20 10 0

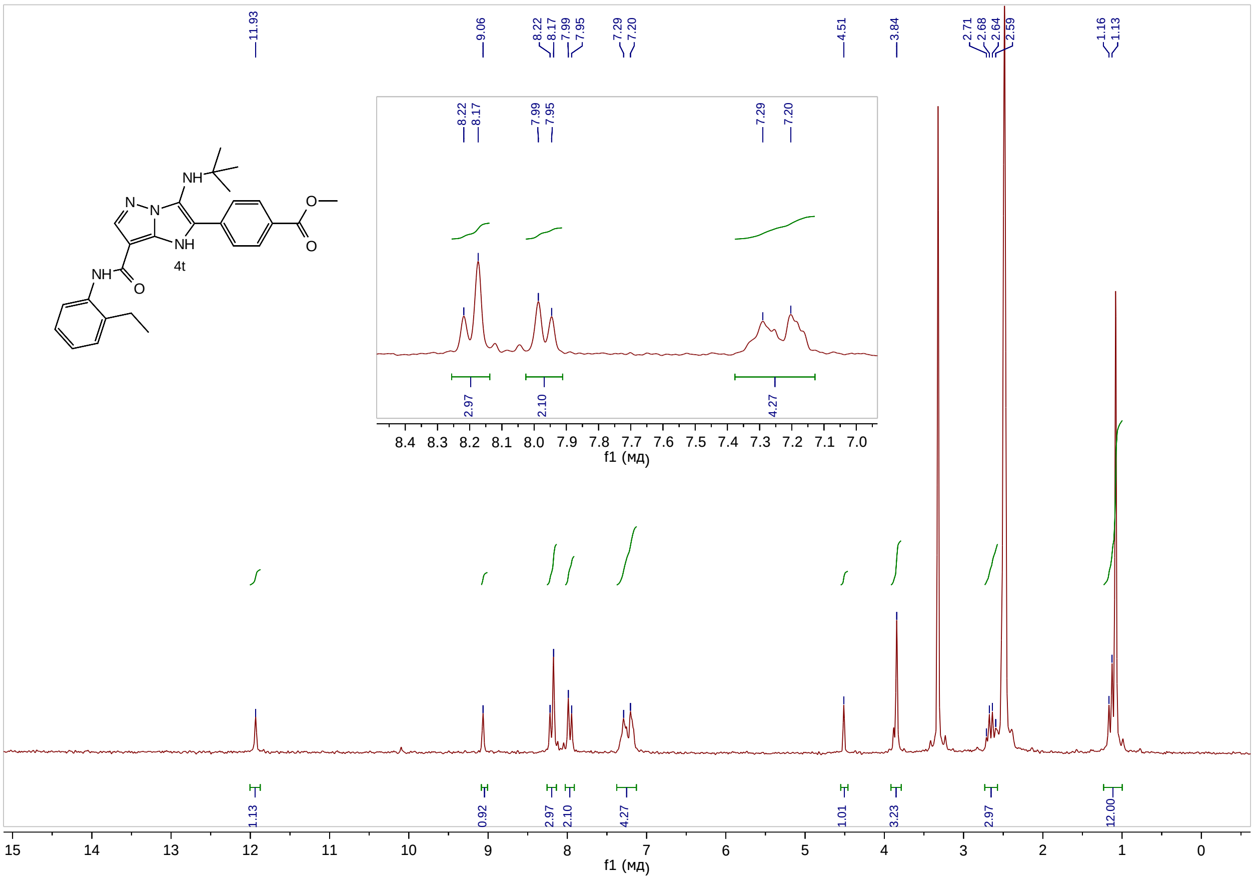
11.93
9.06
8.22
8.17
7.99
7.95
7.29
7.20
4.51
3.84
2.71
2.68
2.64
2.59
1.16
1.13
NH
N
N
O
O
NH
4t
NH
O
1.13
0.92
2.97
2.10
4.27
1.01
3.23
2.97
12.00
f1 (мд)

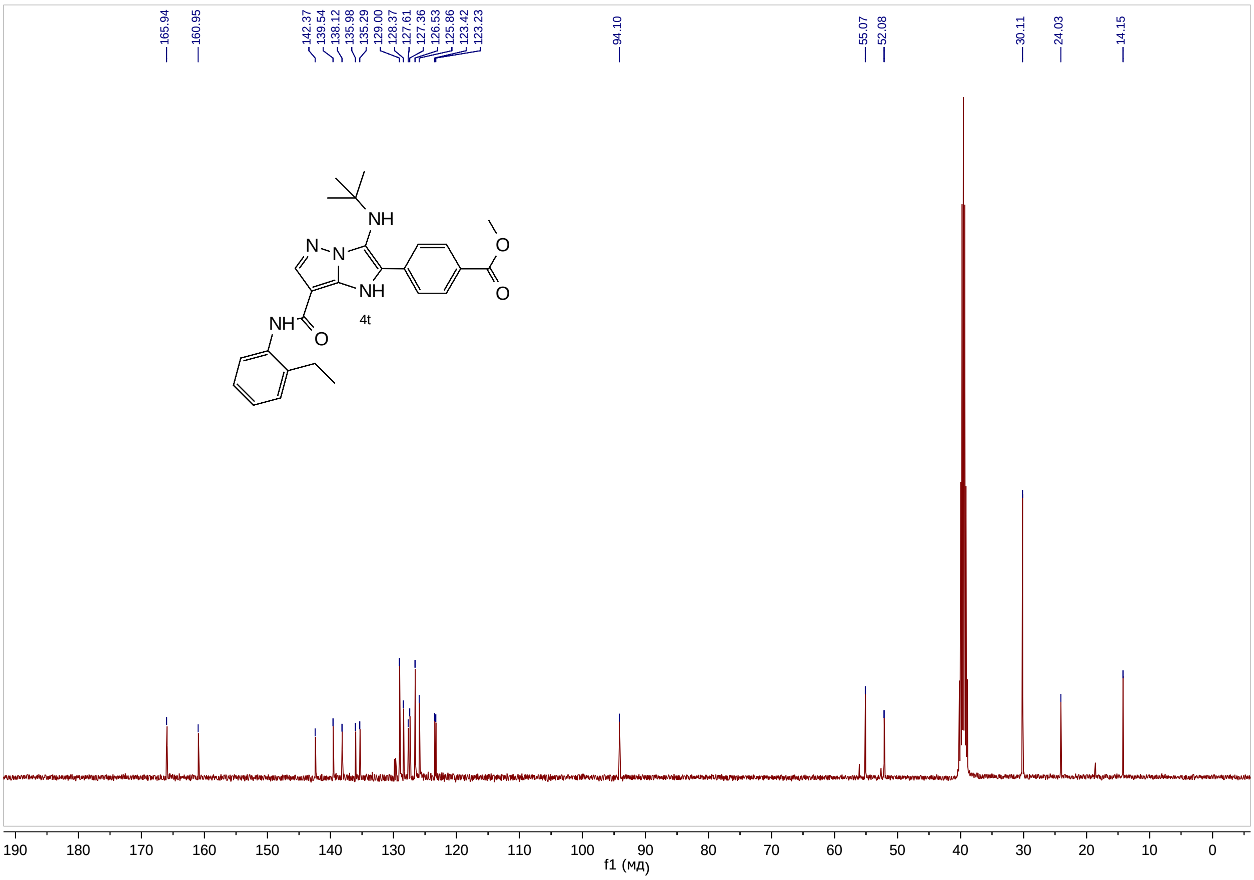
165.94
160.95
142.37
139.54
138.12
135.98
135.29
129.00
128.37
127.61
127.36
126.53
125.86
123.42
123.23
94.10
55.07
52.08
30.11
24.03
14.15
NH
N
N
O
O
NH
4t
NH
O
f1 (мд)

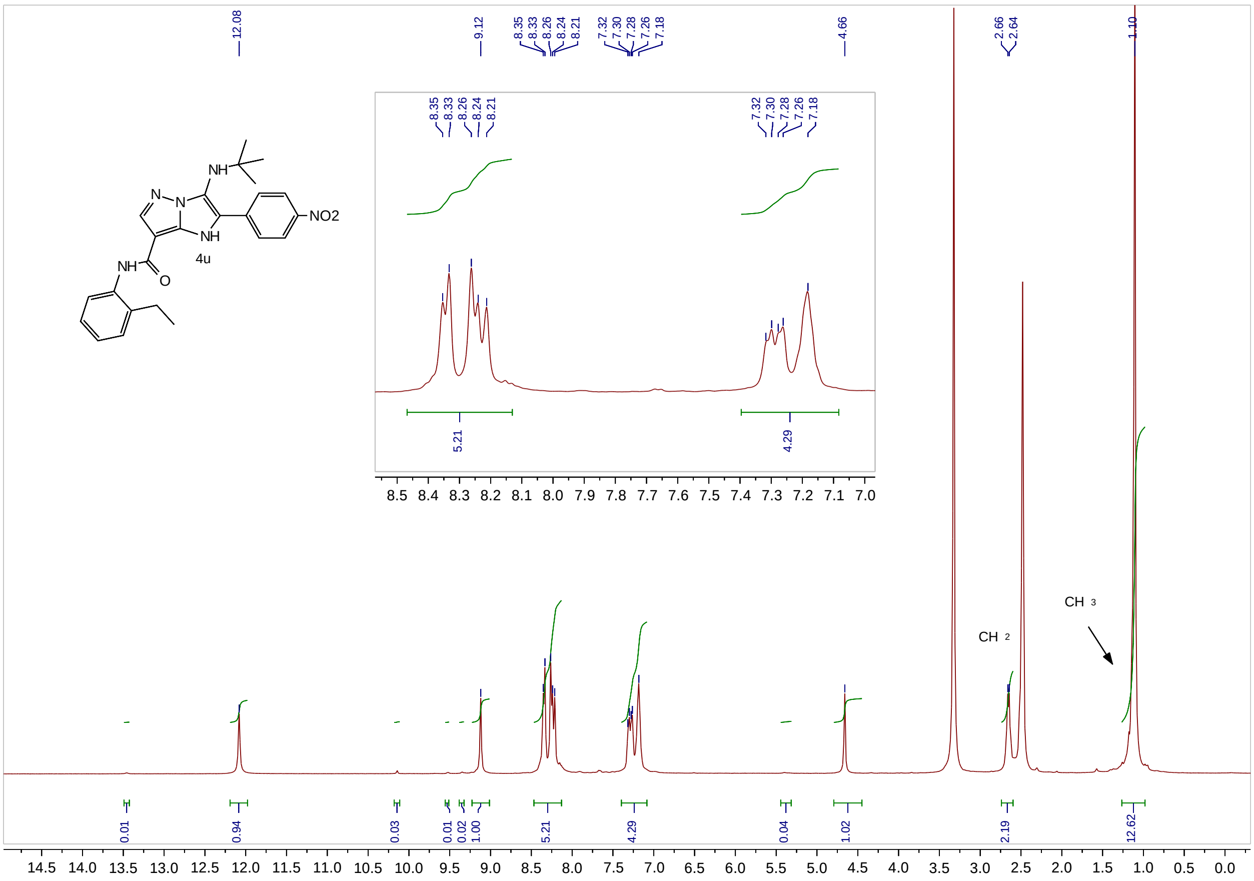
12.08
9.12
8.35
8.33
8.26
8.24
8.21
7.32
7.30
7.28
7.26
7.18
4.66
2.66
2.64
1.10
NH
N
N
NH
NO2
4u
NH
O
5.21
4.29
CH 2
CH 3
0.01
0.94
0.03
0.01
0.02
1.00
5.21
4.29
0.04
1.02
2.19
12.62

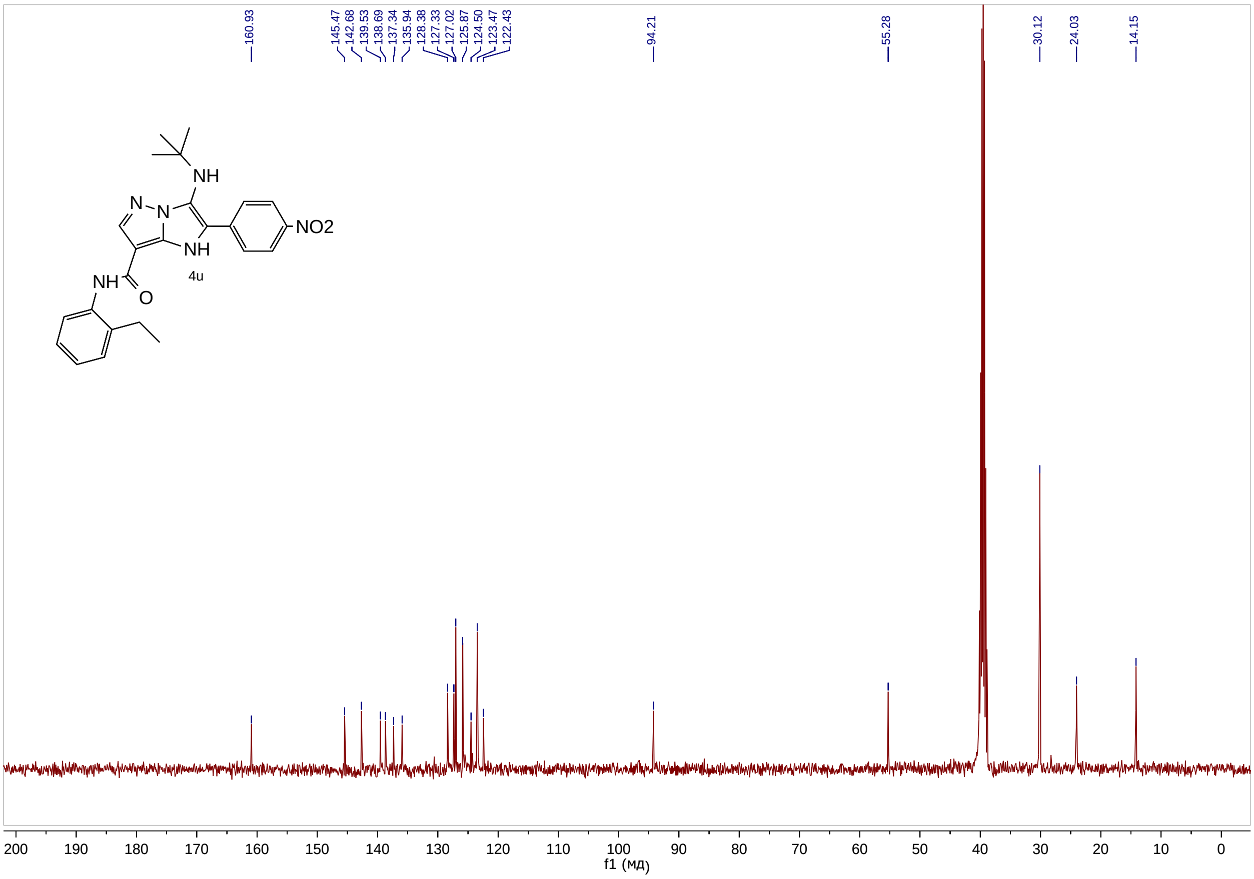
160.93
145.47
142.68
139.53
138.69
137.34
135.94
128.38
127.33
127.02
125.87
124.50
123.47
122.43
94.21
55.28
30.12
24.03
14.15
NH
N
N
NH
NO2
4u
NH
O
f1 (мд)

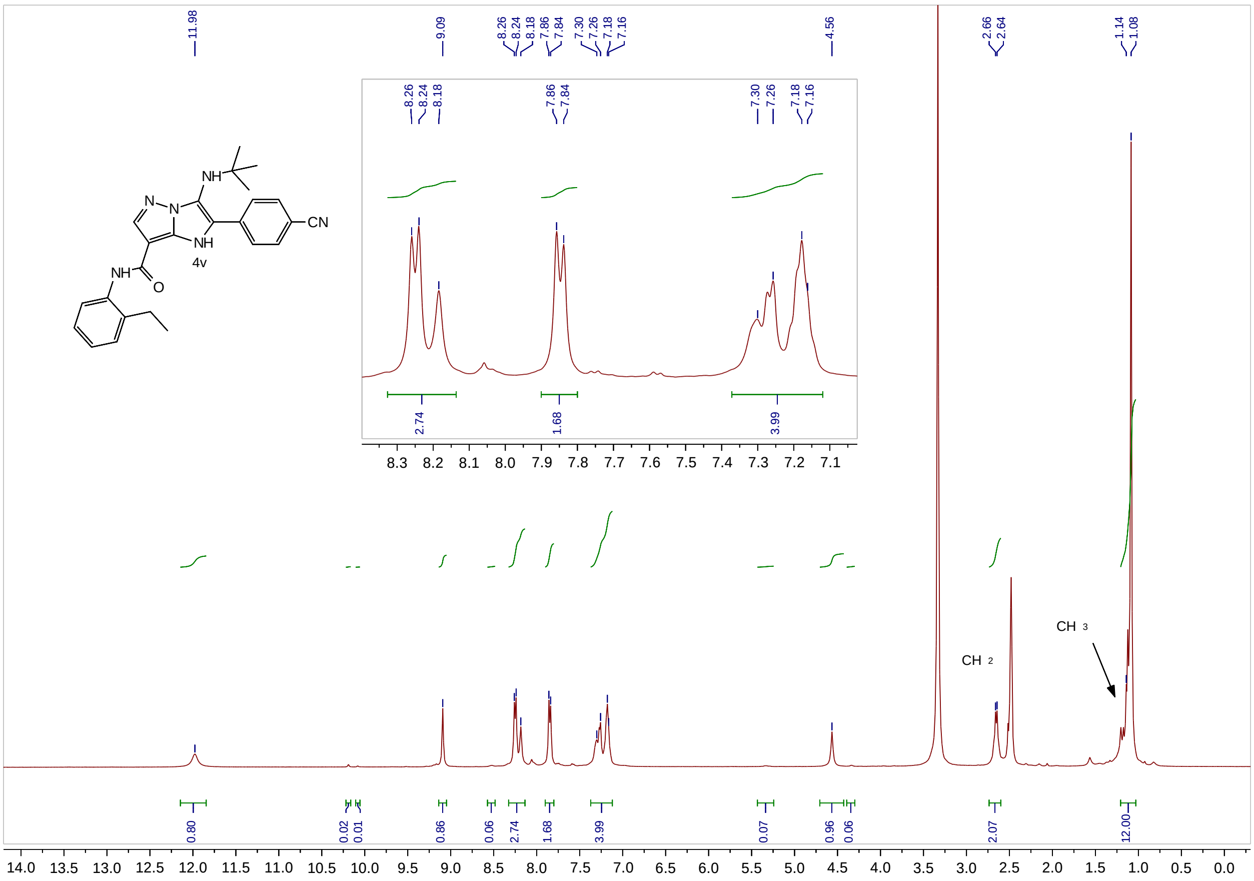

11.98
9.09
8.26
8.24
8.18
7.86
7.84
7.30
7.26
7.18
7.16
4.56
2.66
2.64
1.14
1.08
NH
N
N
NH
CN
4v
NH
O
2.74
1.68
3.99
CH 2
CH 3
0.80
0.02
0.01
0.86
0.06
2.74
1.68
3.99
0.07
0.96
0.06
2.07
12.00

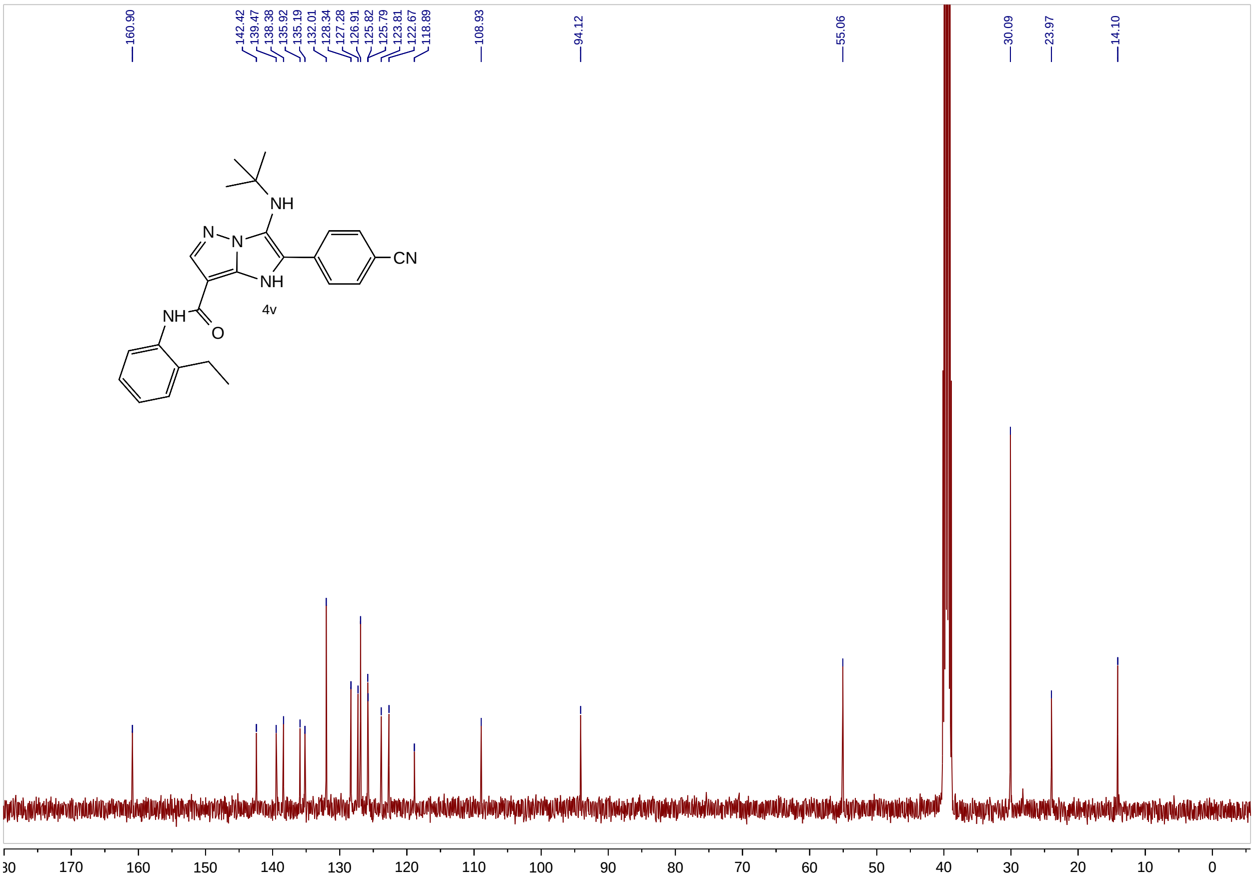

160.90
142.42
139.47
138.38
135.92
135.19
132.01
128.34
127.28
126.91
125.82
125.79
123.81
122.67
118.89
108.93
94.12
55.06
30.09
23.97
14.10
NH
N
N
NH
CN
4v
NH
O

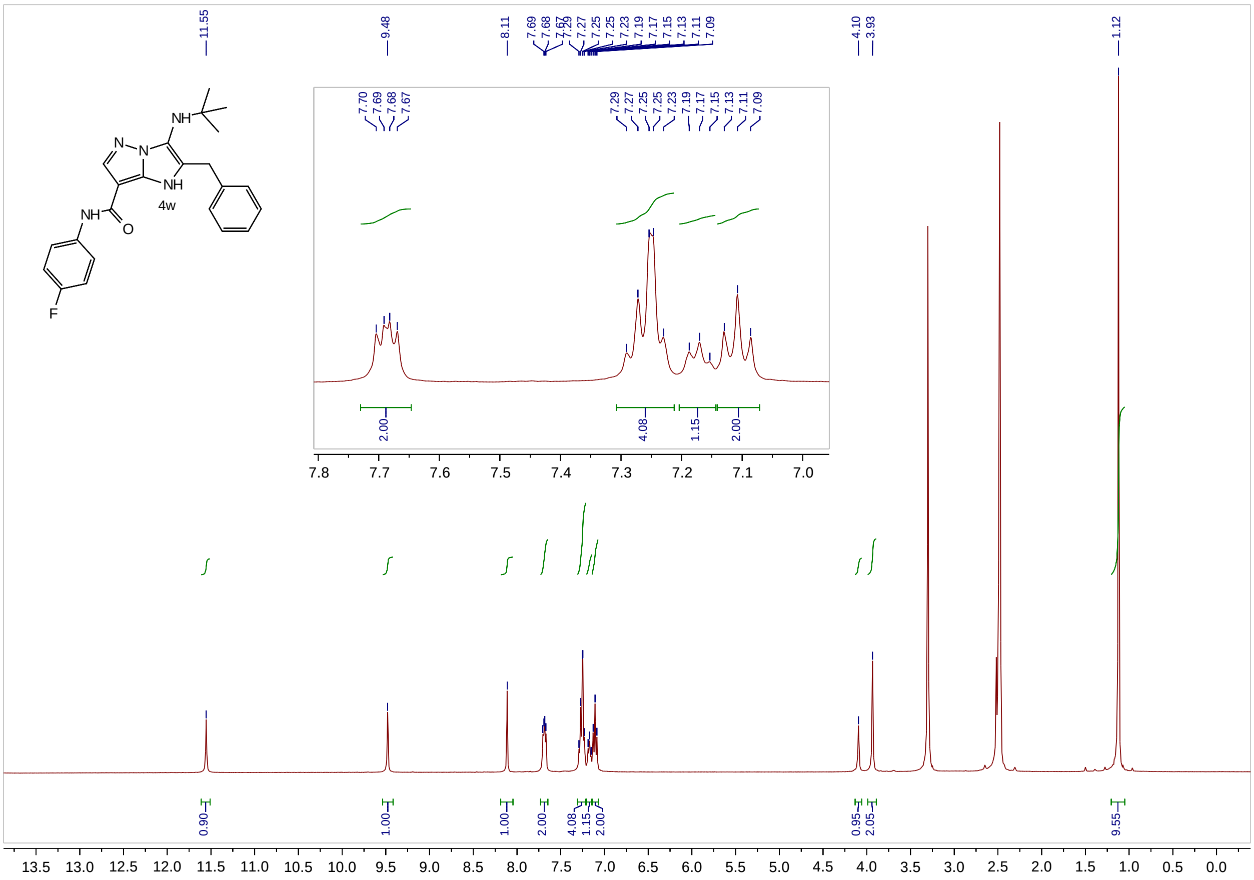
11.55
9.48
8.11
7.69 7.68 7.67 7.67 7.27 7.25 7.25 7.23 7.19 7.17 7.15 7.13 7.11 7.09
4.10 3.93
1.12
7.70 7.69 7.68 7.67
7.29 7.27 7.25 7.25 7.23 7.19 7.17 7.15 7.13 7.11 7.09
2.00
4.08
1.15
2.00
4w
0.90
1.00
1.00
2.00
4.08
1.15
2.00
0.95
2.05
9.55

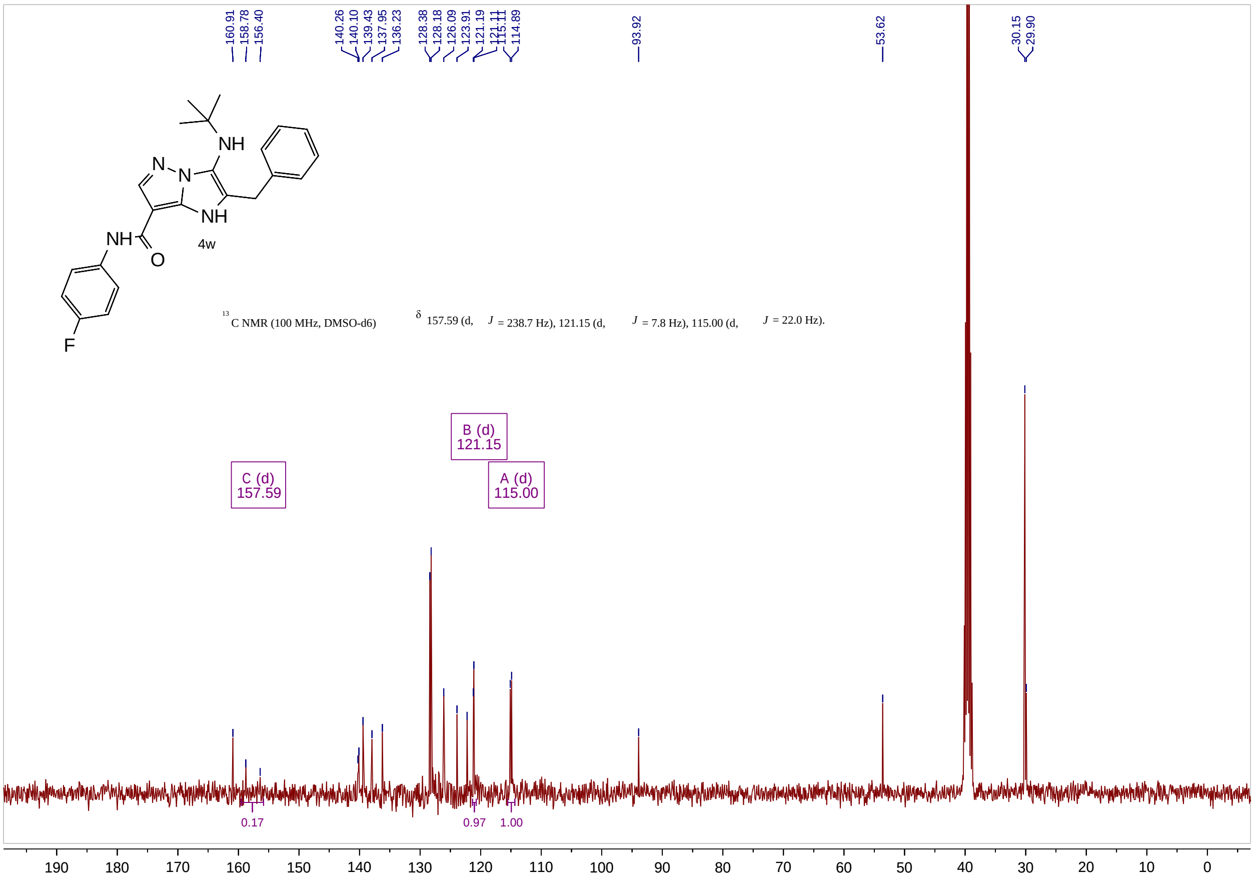
160.91 158.78 156.40
140.26 140.10 139.43 137.95 136.23
128.38 128.18 126.09 123.91 121.19 121.11 115.11 114.89
93.92
53.62
30.15 29.90
4w
13C NMR (100 MHz, DMSO-d6) δ 157.59 (d, J = 238.7 Hz), 121.15 (d, J = 7.8 Hz), 115.00 (d, J = 22.0 Hz).
B (d) 121.15
C (d) 157.59
A (d) 115.00
0.17
0.97
1.00

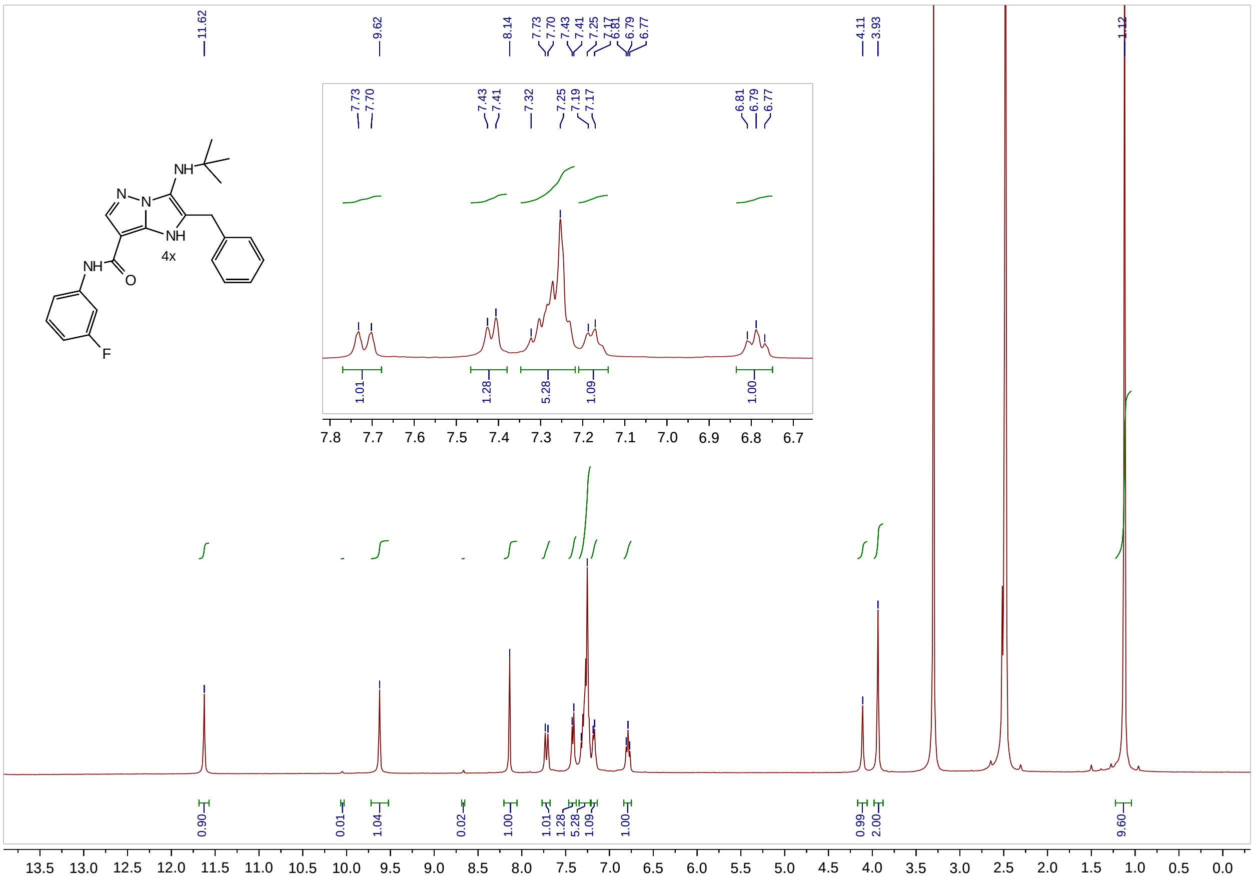
4x
NH
N
N
NH
NH
O
F

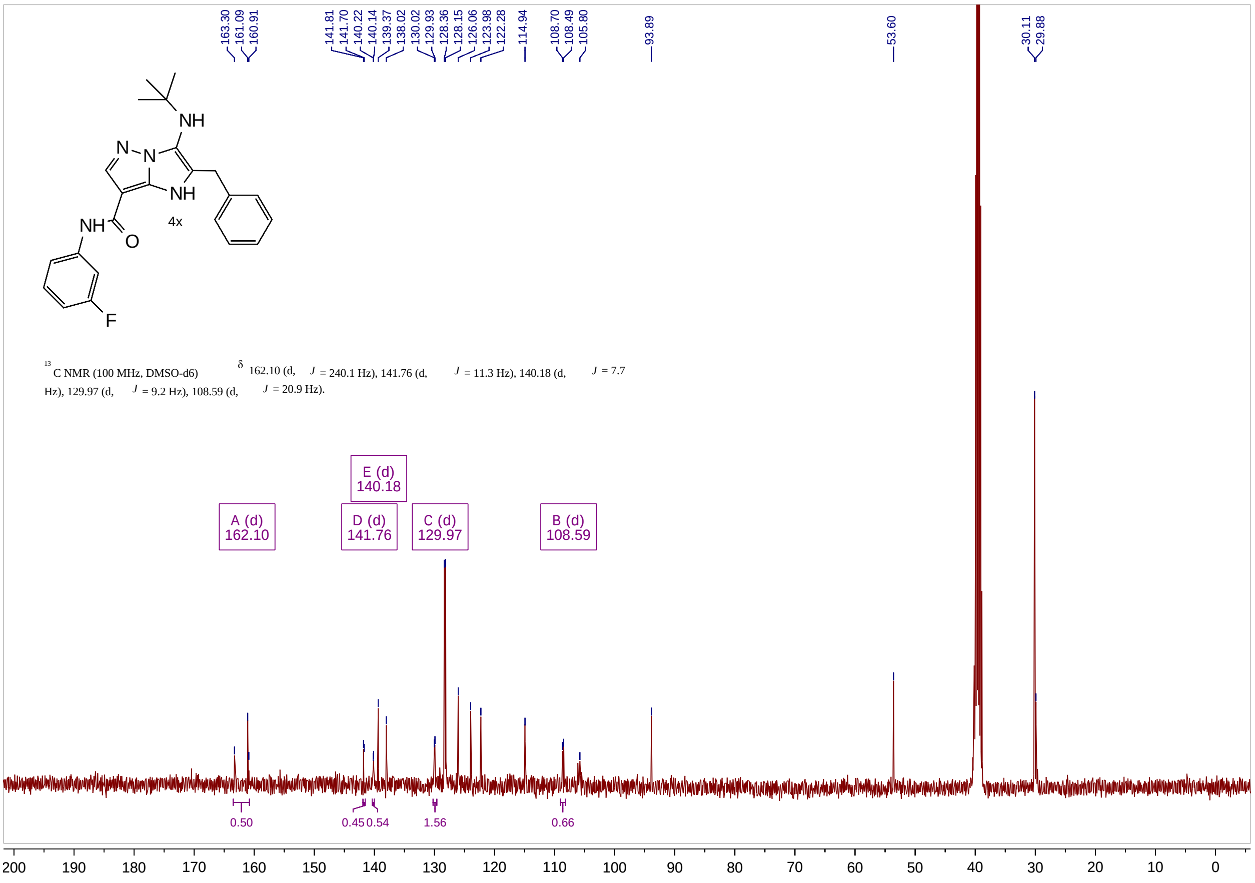
4x
NH
N
N
NH
NH
O
F
^{13}C NMR (100 MHz, DMSO-d6) δ 162.10 (d, J = 240.1 Hz), 141.76 (d, J = 11.3 Hz), 140.18 (d, J = 7.7 Hz), 129.97 (d, J = 9.2 Hz), 108.59 (d, J = 20.9 Hz).
E (d) 140.18
A (d) 162.10
D (d) 141.76
C (d) 129.97
B (d) 108.59

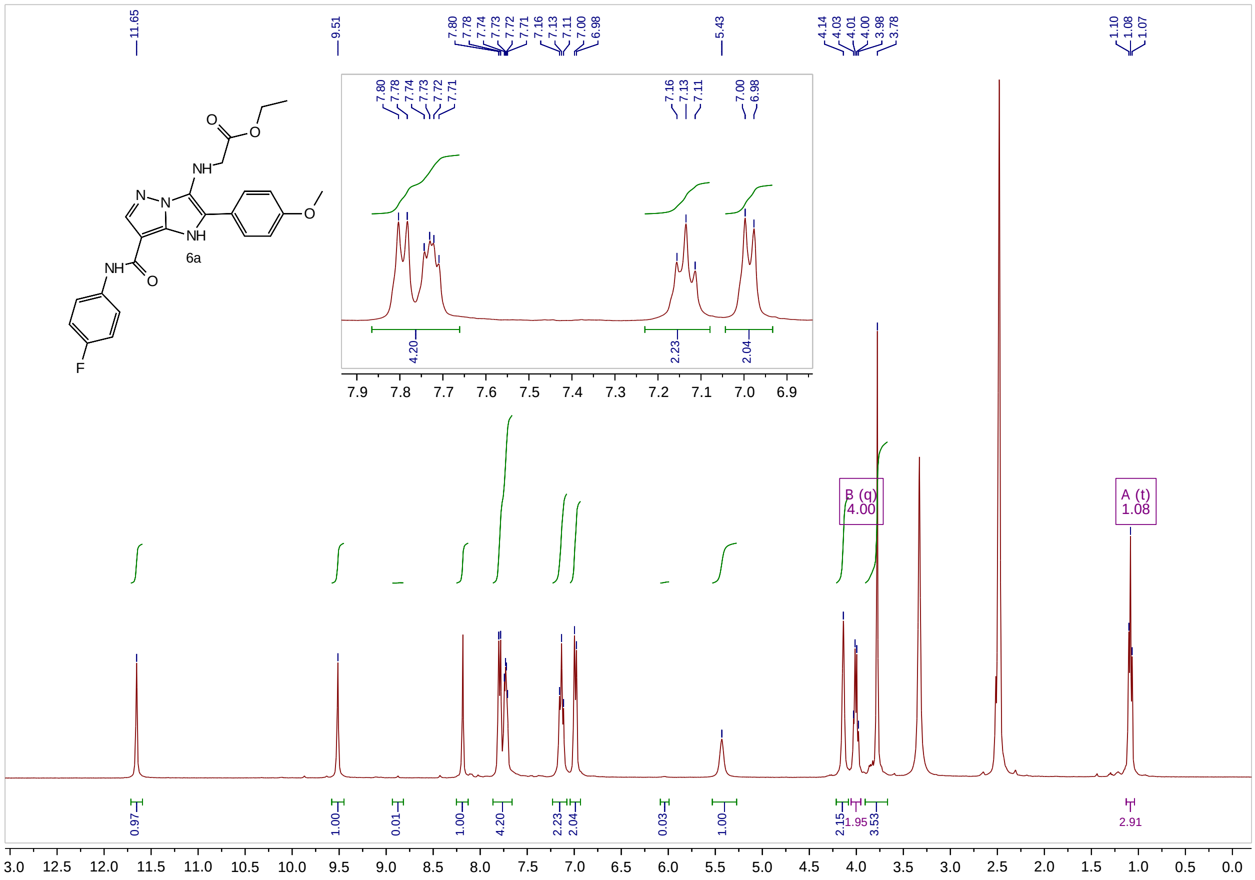
6a

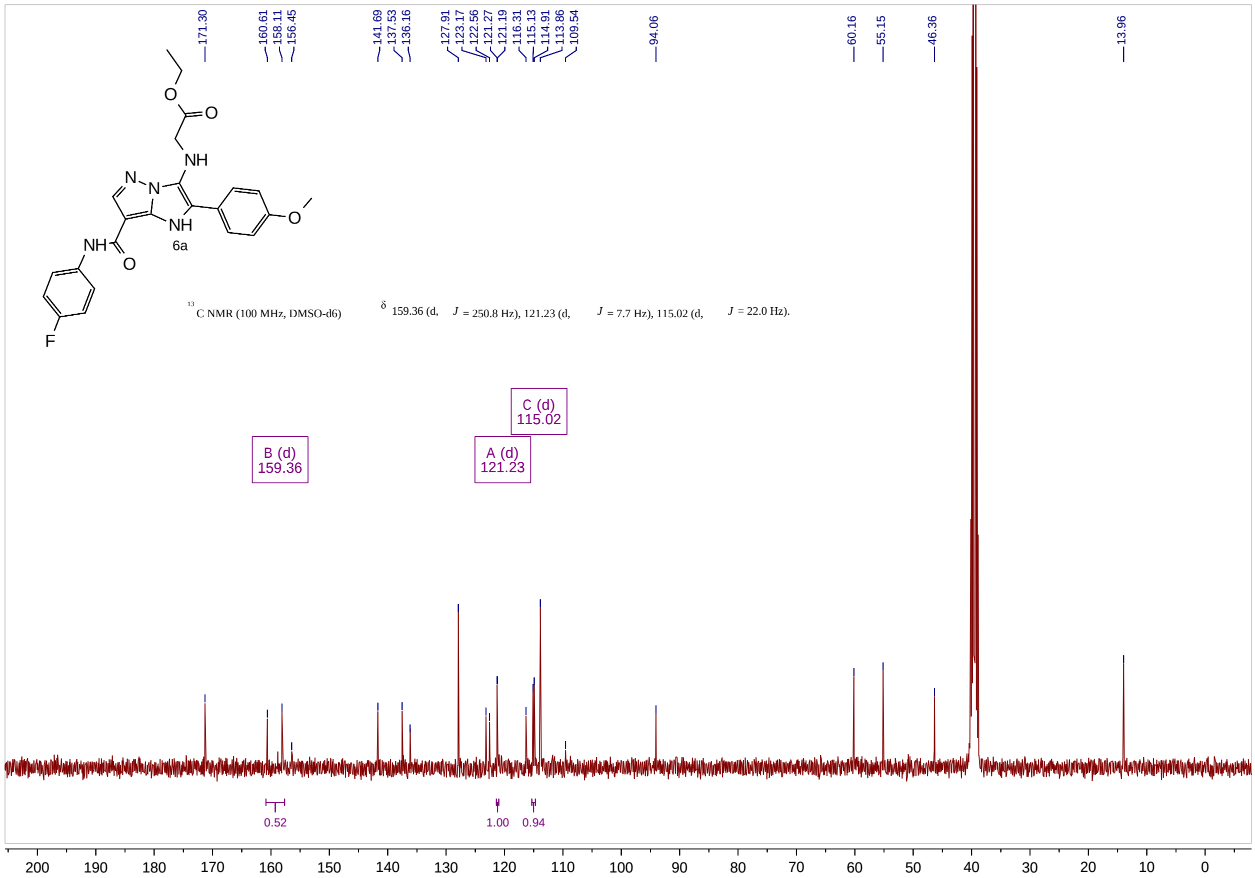
6a
^{13}C NMR (100 MHz, DMSO-d6) δ 159.36 (d, J = 250.8 Hz), 121.23 (d, J = 7.7 Hz), 115.02 (d, J = 22.0 Hz).

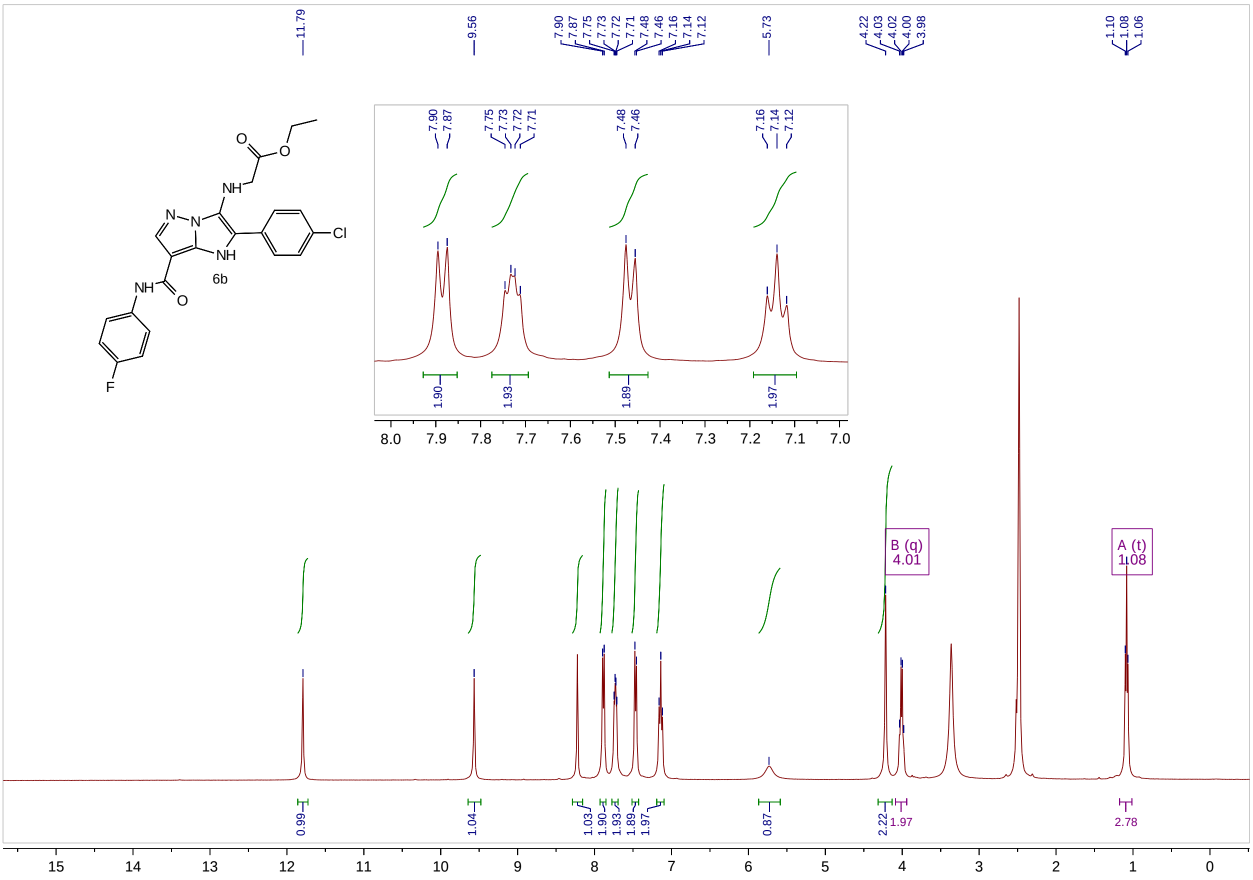
11.79
9.56
7.90 7.87 7.75 7.73 7.72 7.71 7.48 7.46 7.16 7.14 7.12
5.73
4.22 4.03 4.02 4.00 3.98
1.10 1.08 1.06
6b
B (q)
4.01
A (t)
1.08
0.99 1.04 1.03 1.90 1.93 1.89 1.97 0.87 2.22 1.97 2.78

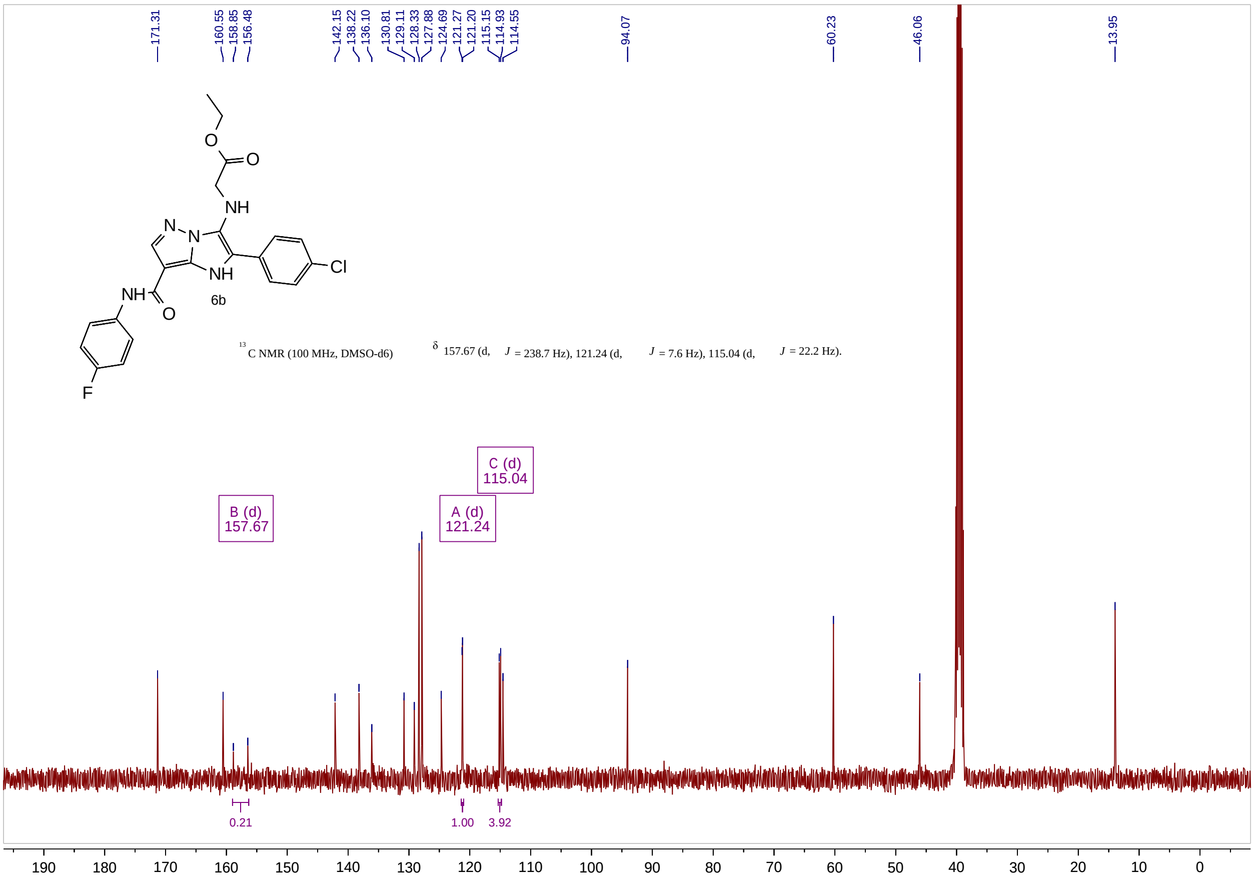
171.31
160.55 158.85 156.48
142.15 138.22 136.10 130.81 129.11 128.33 127.88 124.69 121.27 121.20 115.15 114.93 114.55
94.07
60.23
46.06
13.95
6b
^{13}C NMR (100 MHz, DMSO-d6) δ 157.67 (d, J = 238.7 Hz), 121.24 (d, J = 7.6 Hz), 115.04 (d, J = 22.2 Hz).
B (d)
157.67
A (d)
121.24
C (d)
115.04
0.21 1.00 3.92

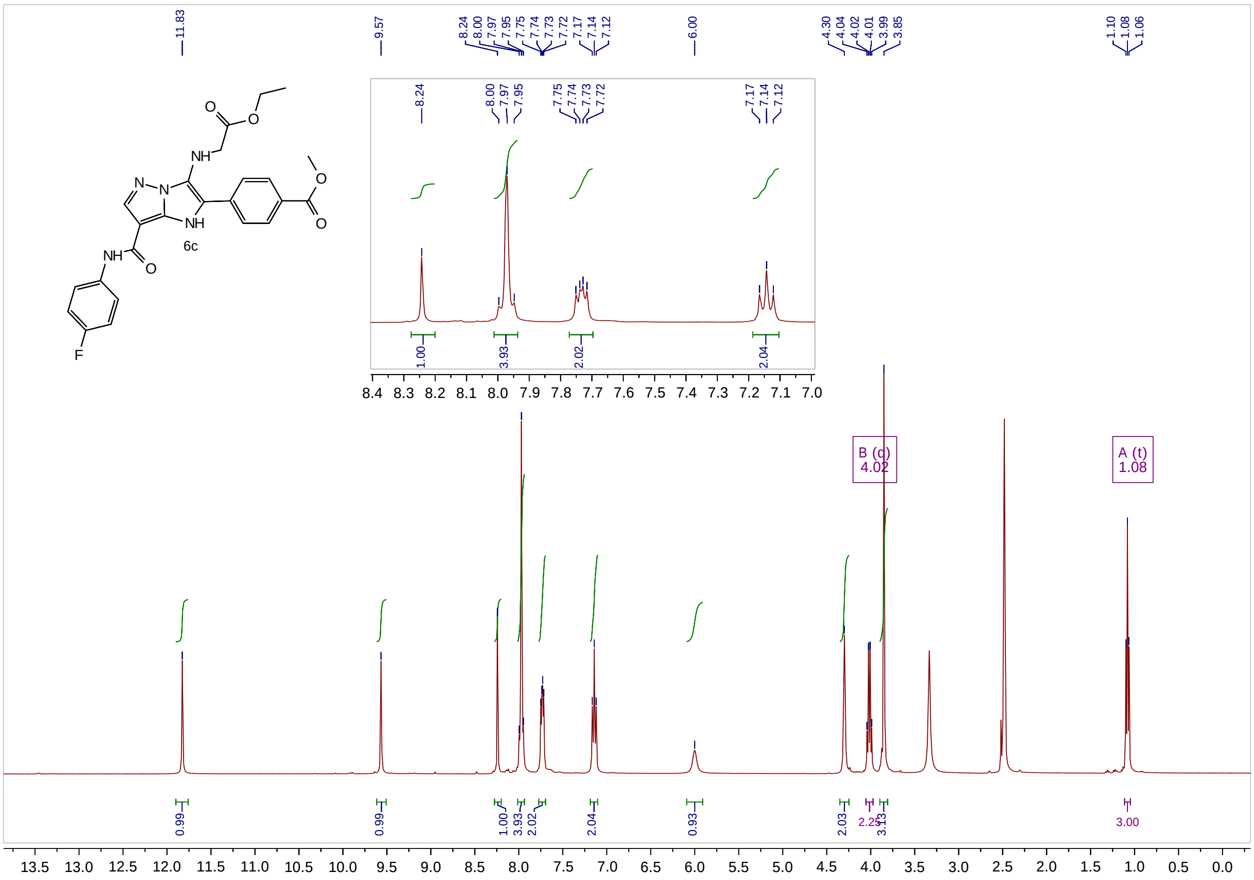
6c
A (t)
1.08
B (q)
4.02

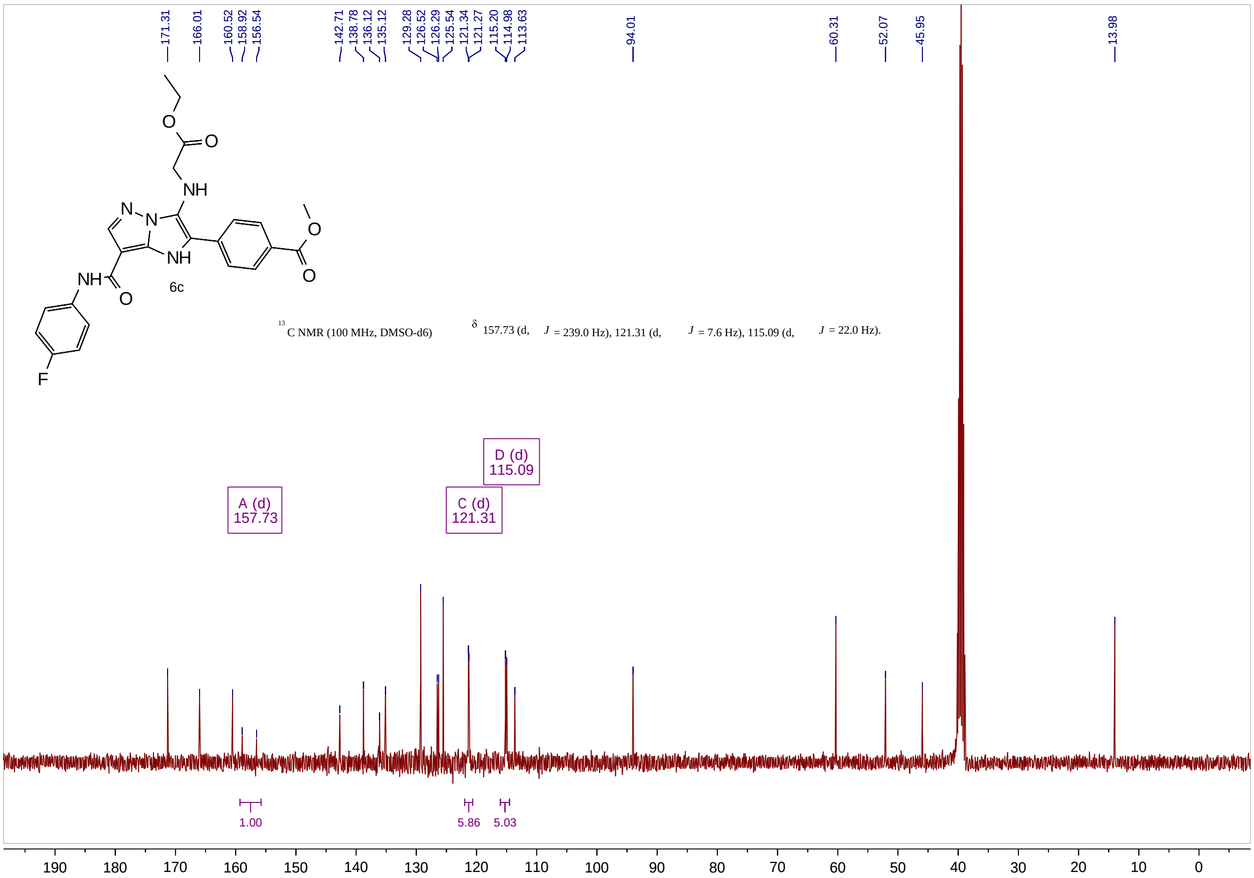
6c
13C NMR (100 MHz, DMSO-d6) δ 157.73 (d, J = 239.0 Hz), 121.31 (d, J = 7.6 Hz), 115.09 (d, J = 22.0 Hz).
A (d)
157.73
C (d)
121.31
D (d)
115.09

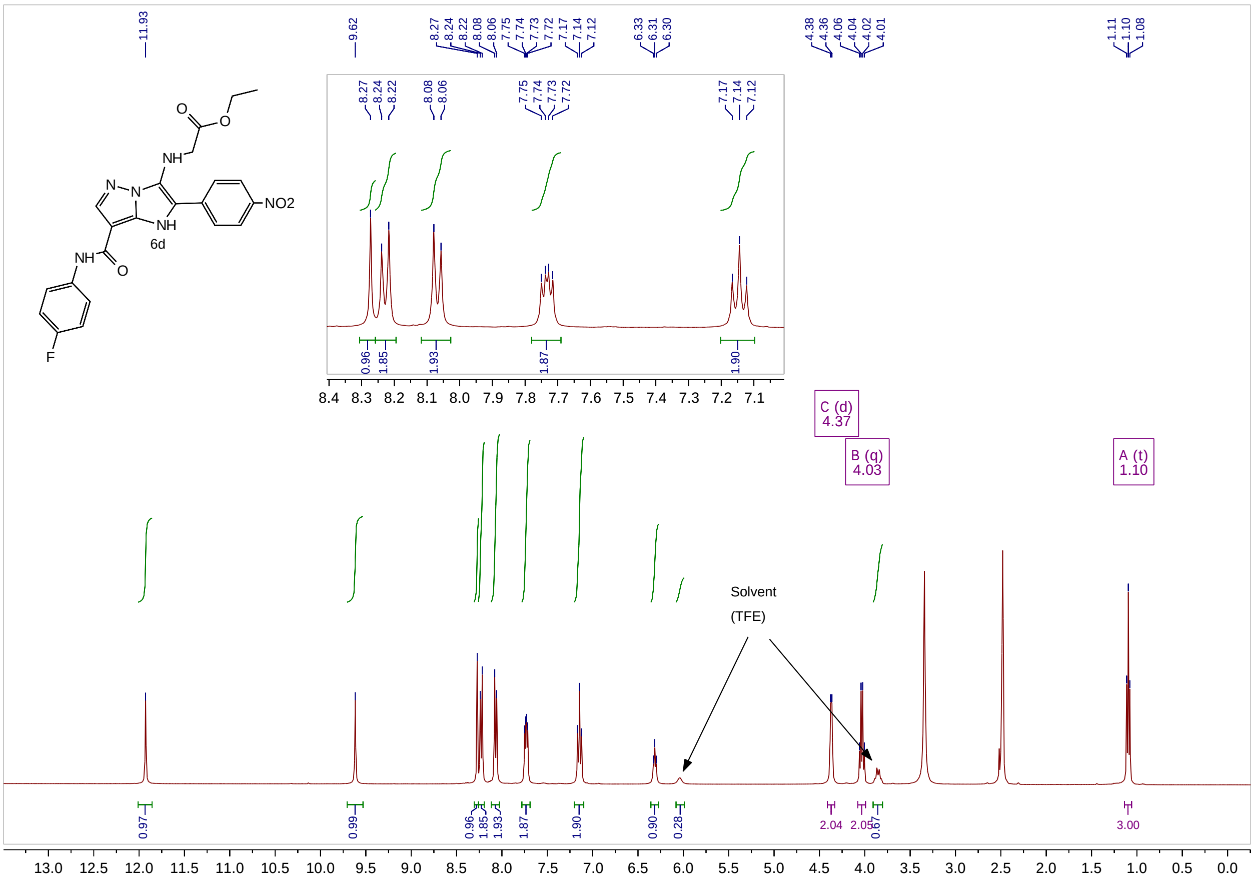

NO2
NH
6d
F
C (d)
4.37
B (q)
4.03
A (t)
1.10
Solvent
(TFE)

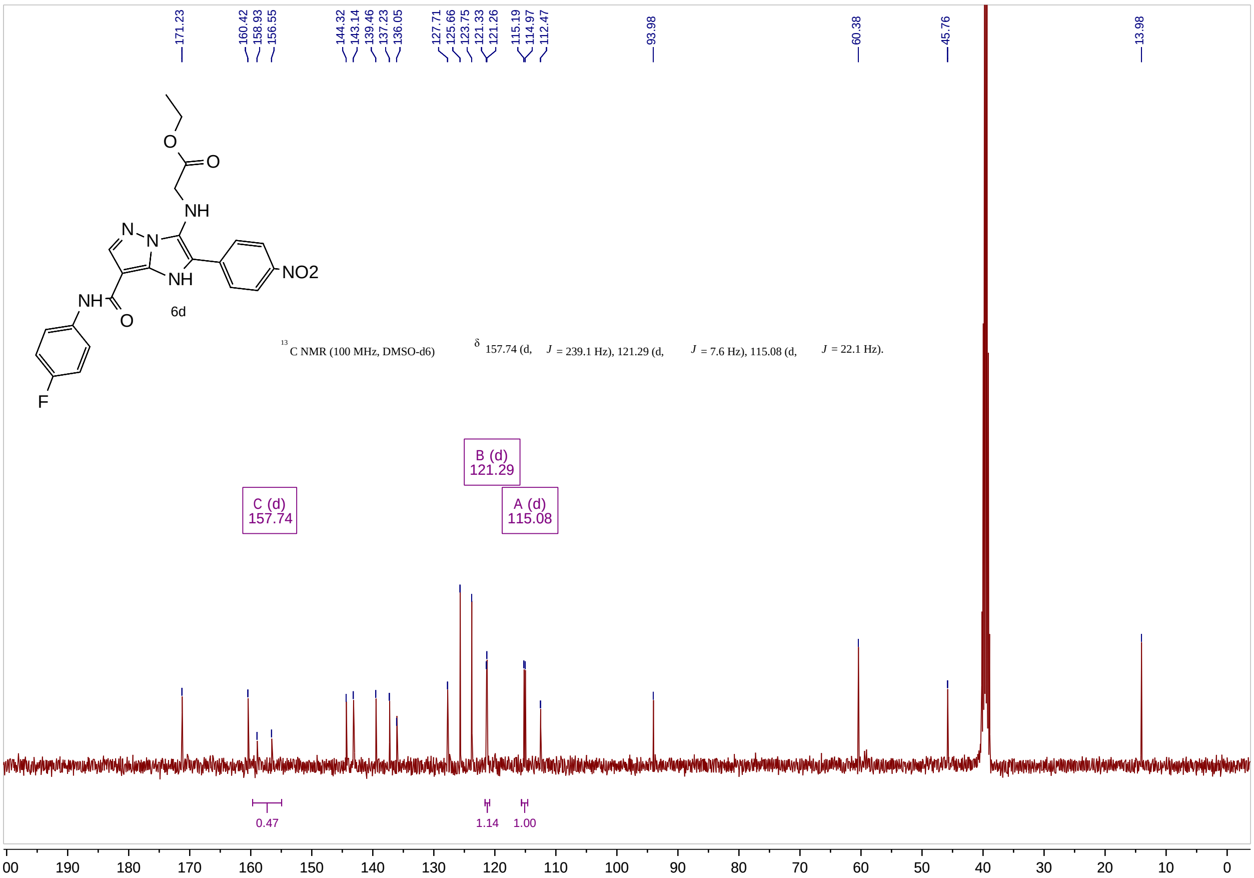

NO2
NH
6d
F
^{13}C NMR (100 MHz, DMSO-d6) δ 157.74 (d, J = 239.1 Hz), 121.29 (d, J = 7.6 Hz), 115.08 (d, J = 22.1 Hz).
B (d)
121.29
C (d)
157.74
A (d)
115.08

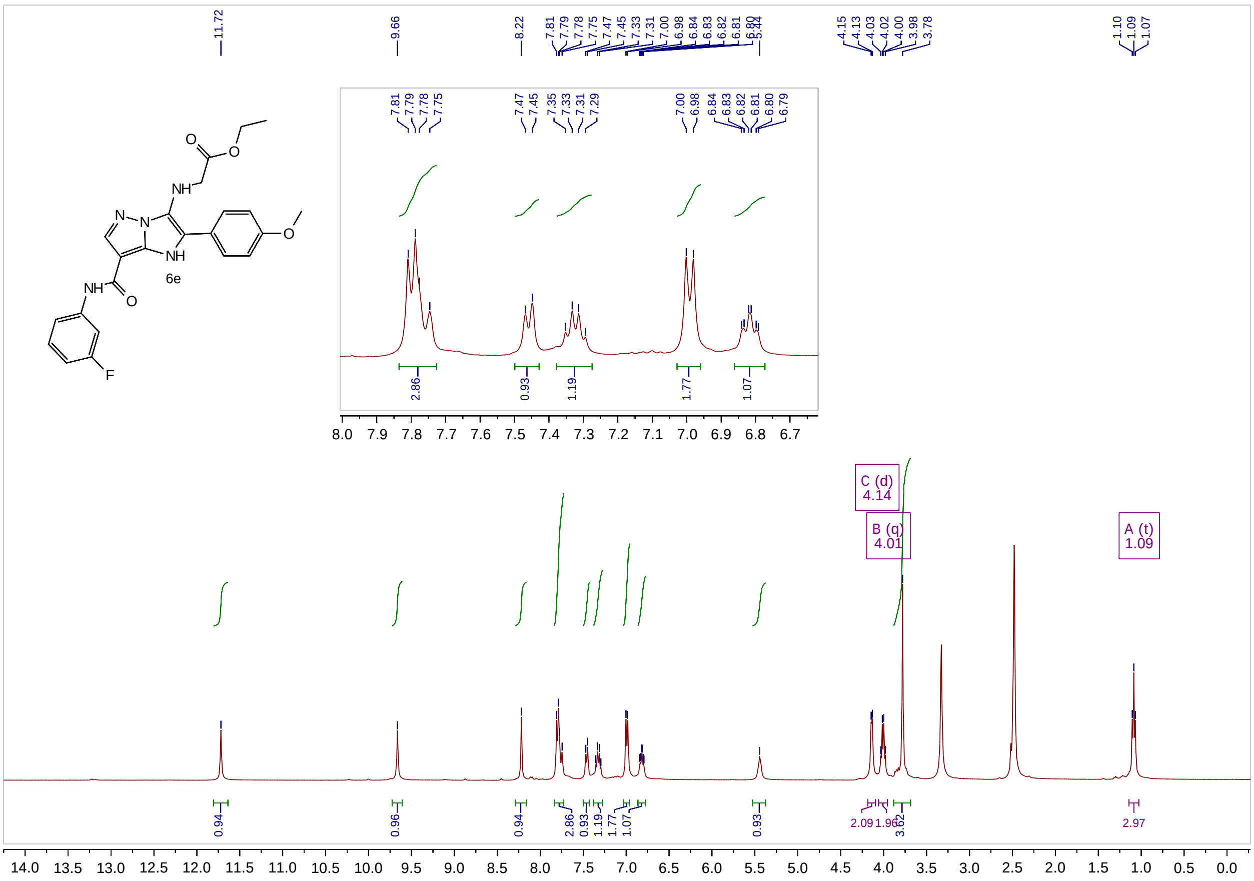
6e
C (d)
4.14
B (q)
4.01
A (t)
1.09

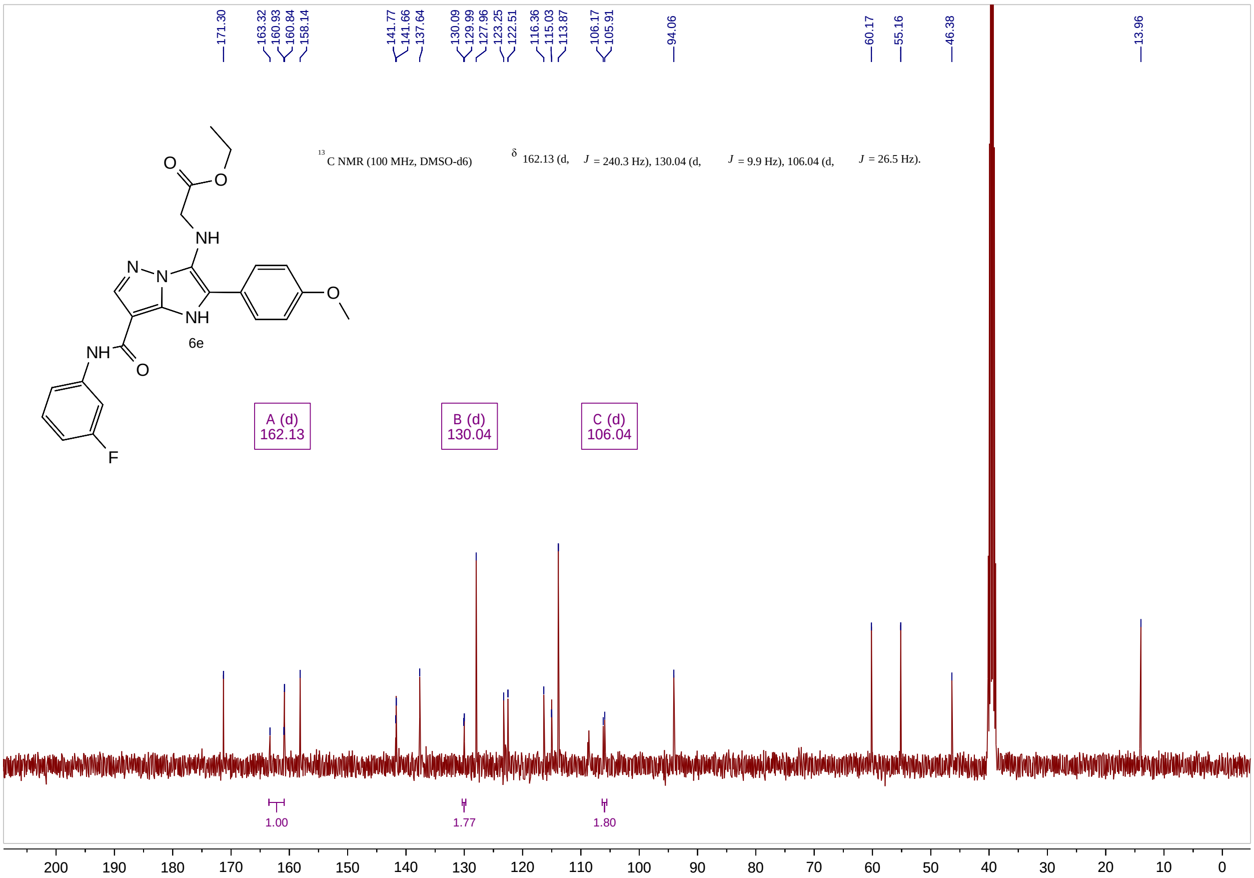
6e
13C NMR (100 MHz, DMSO-d6) δ 162.13 (d, J = 240.3 Hz), 130.04 (d, J = 9.9 Hz), 106.04 (d, J = 26.5 Hz).
A (d)
162.13
B (d)
130.04
C (d)
106.04

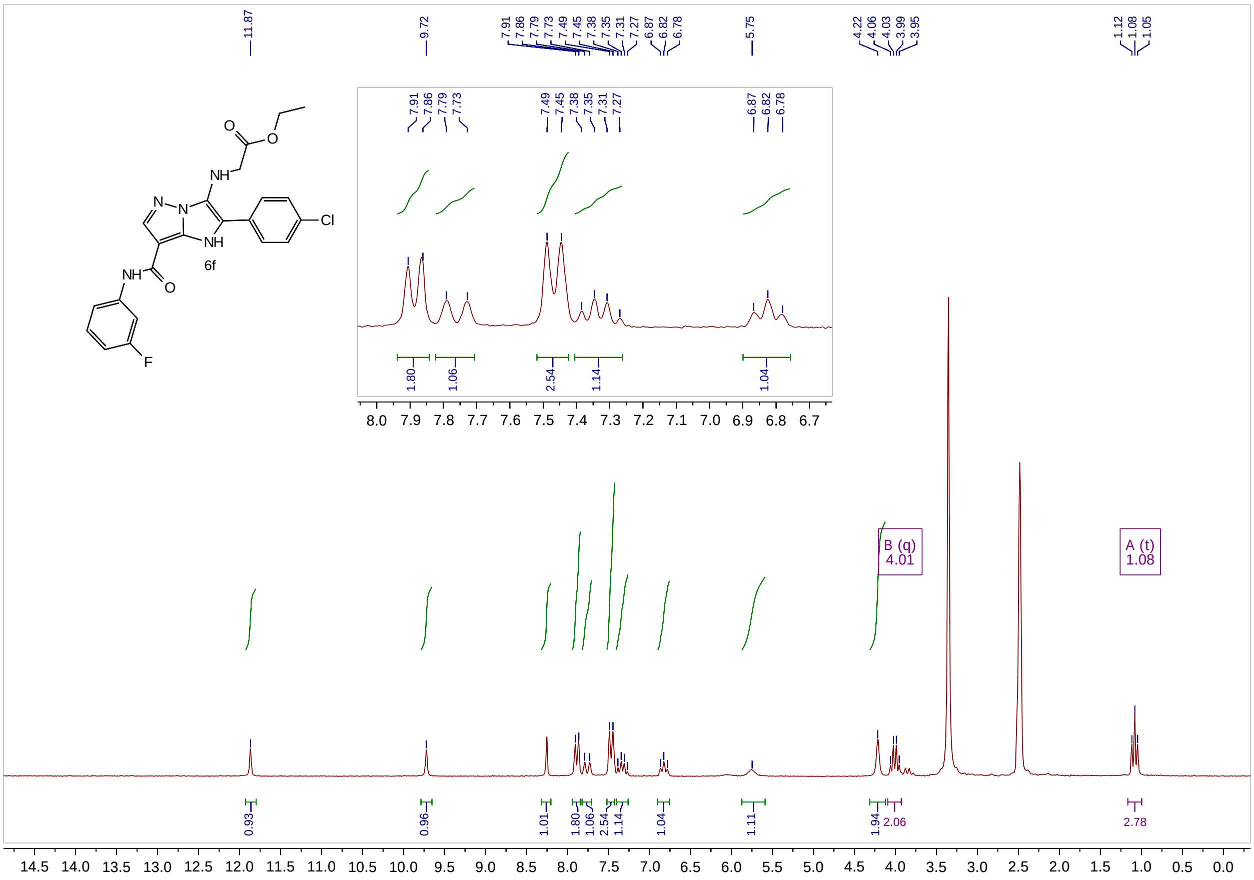
11.87
9.72
7.91 7.86 7.79 7.73 7.49 7.45 7.38 7.35 7.31 7.27 6.87 6.82 6.78
5.75
4.22 4.06 4.03 3.99 3.95
1.12 1.08 1.05
NH
O
O
N
N
NH
Cl
6f
NH
O
F
B (q)
4.01
A (t)
1.08
0.93
0.96
1.01
1.80
1.06
2.54
1.14
1.04
1.11
1.94
2.06
2.78

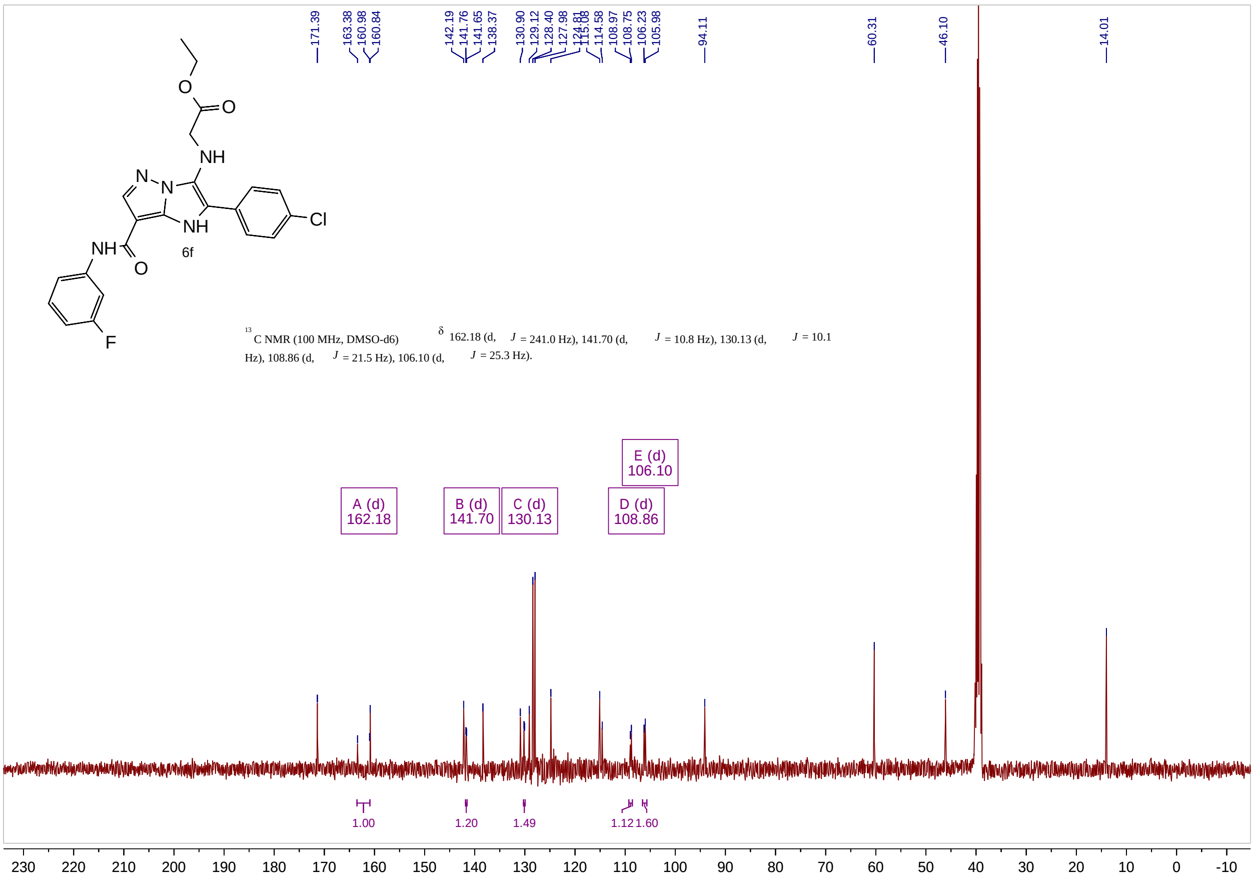
171.39
163.38 160.98 160.84
142.19 141.76 141.65 138.37
130.90 129.12 128.40 127.98 124.81 115.08 114.58 108.97 108.75 106.23 105.98
94.11
60.31
46.10
14.01
6f
F
Cl
13C NMR (100 MHz, DMSO-d6) δ 162.18 (d, J = 241.0 Hz), 141.70 (d, J = 10.8 Hz), 130.13 (d, J = 10.1 Hz), 108.86 (d, J = 21.5 Hz), 106.10 (d, J = 25.3 Hz).
A (d)
162.18
B (d)
141.70
C (d)
130.13
D (d)
108.86
E (d)
106.10

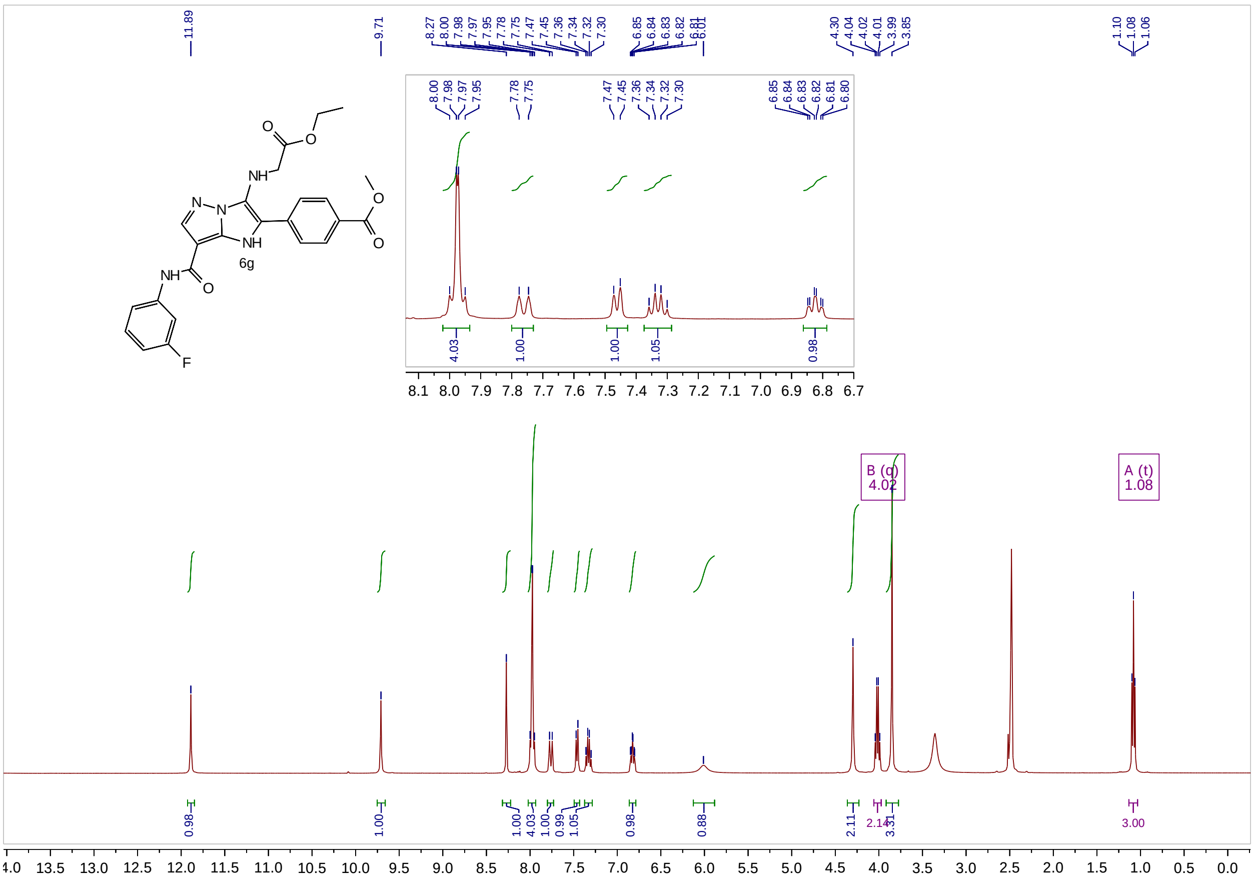
11.89
9.71
8.27
8.00
7.98
7.97
7.95
7.78
7.75
7.47
7.45
7.36
7.34
7.32
7.30
6.85
6.84
6.83
6.82
6.81
6.01
4.30
4.04
4.02
4.01
3.99
3.85
1.10
1.08
1.06
6g
NH
N
O
F
B (q)
4.02
A (t)
1.08

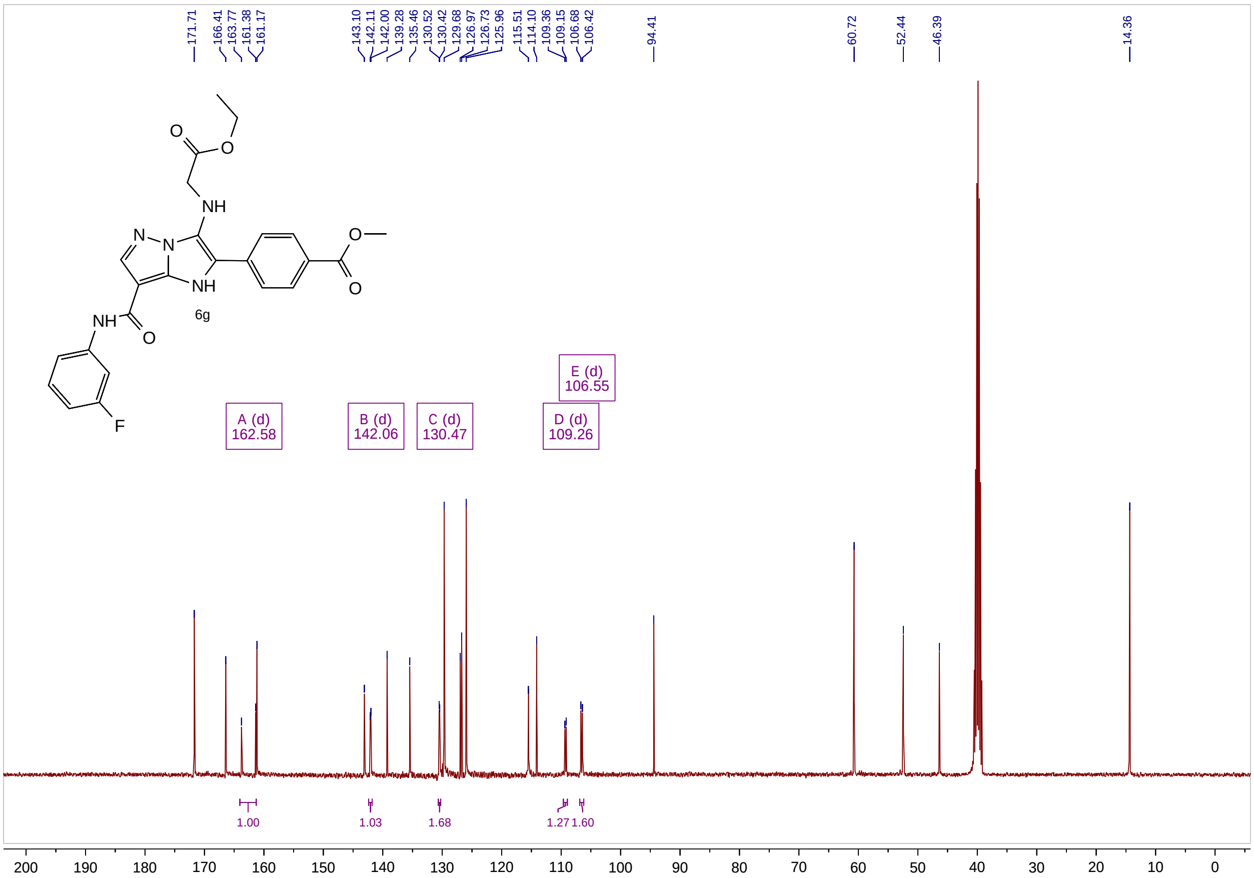
171.71
166.41
163.77
161.38
161.17
143.10
142.11
142.00
139.28
135.46
130.52
130.42
129.68
126.97
126.73
125.96
115.51
114.10
109.36
109.15
106.68
106.42
94.41
60.72
52.44
46.39
14.36
6g
A (d)
162.58
B (d)
142.06
C (d)
130.47
D (d)
109.26
E (d)
106.55

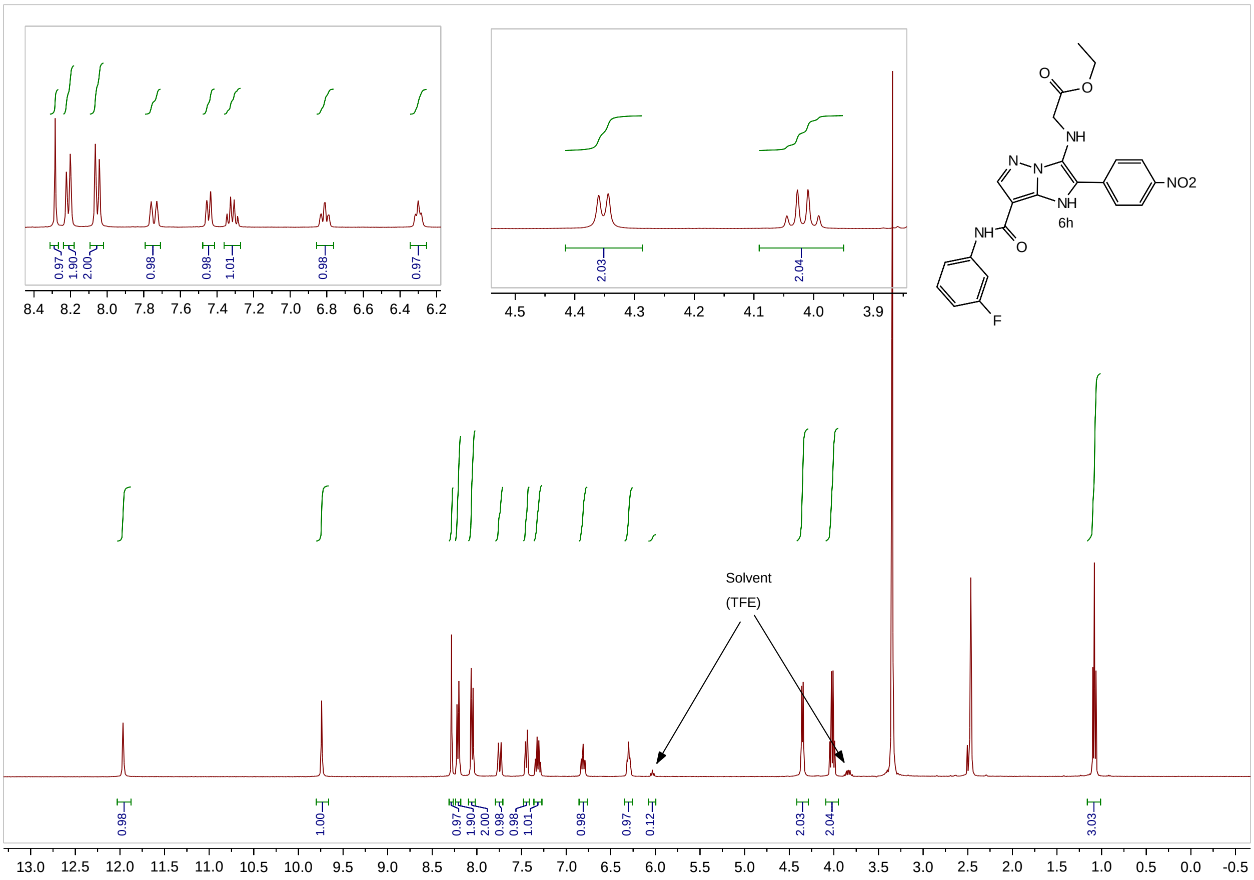

Solvent
(TFE)
NH
O
O
O
N
N
NO2
6h
F

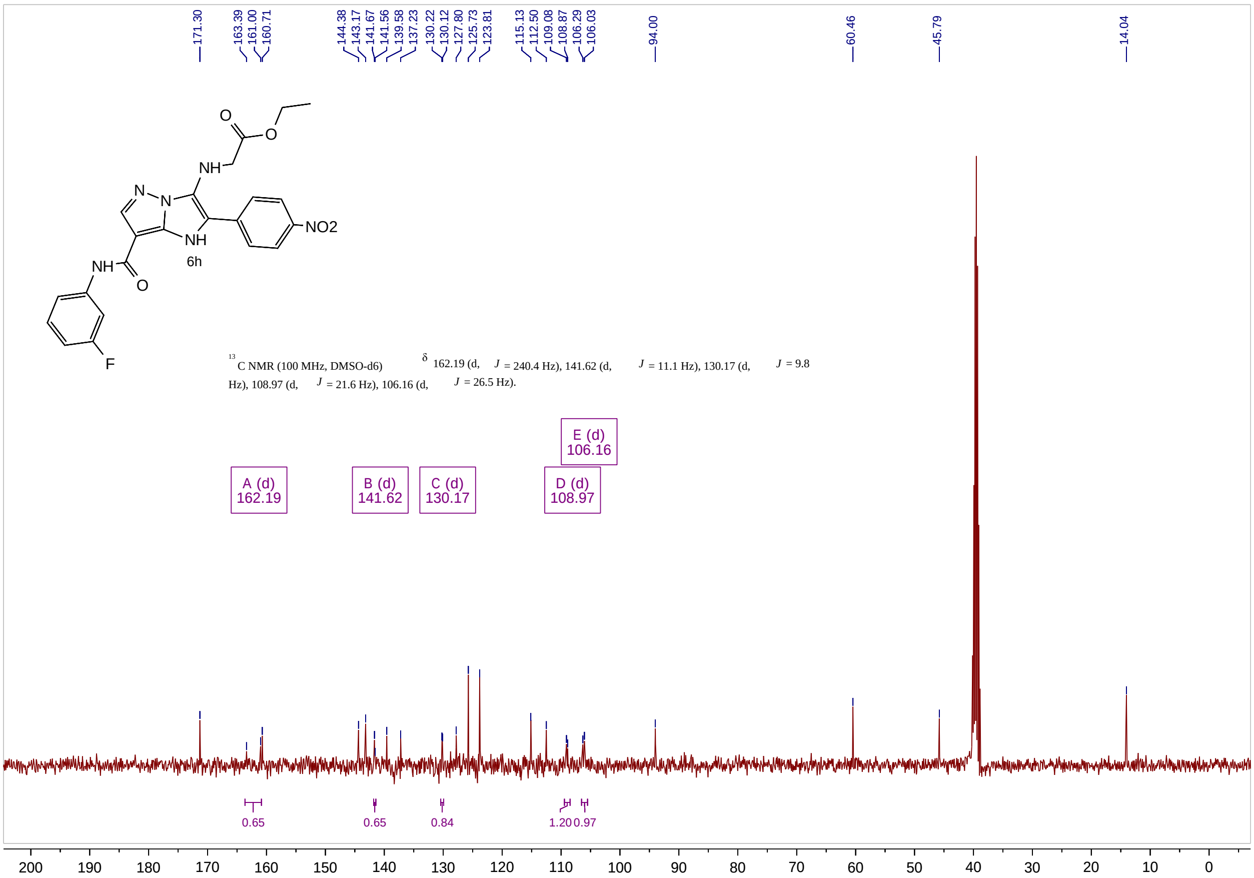

171.30
163.39
161.00
160.71
144.38
143.17
141.67
141.56
139.58
137.23
130.22
130.12
127.80
125.73
123.81
115.13
112.50
109.08
108.87
106.29
106.03
94.00
60.46
45.79
14.04
NO2
6h
F
^{13}C NMR (100 MHz, DMSO-d6) δ 162.19 (d, J = 240.4 Hz), 141.62 (d, J = 11.1 Hz), 130.17 (d, J = 9.8 Hz), 108.97 (d, J = 21.6 Hz), 106.16 (d, J = 26.5 Hz).
A (d) 162.19
B (d) 141.62
C (d) 130.17
D (d) 108.97
E (d) 106.16

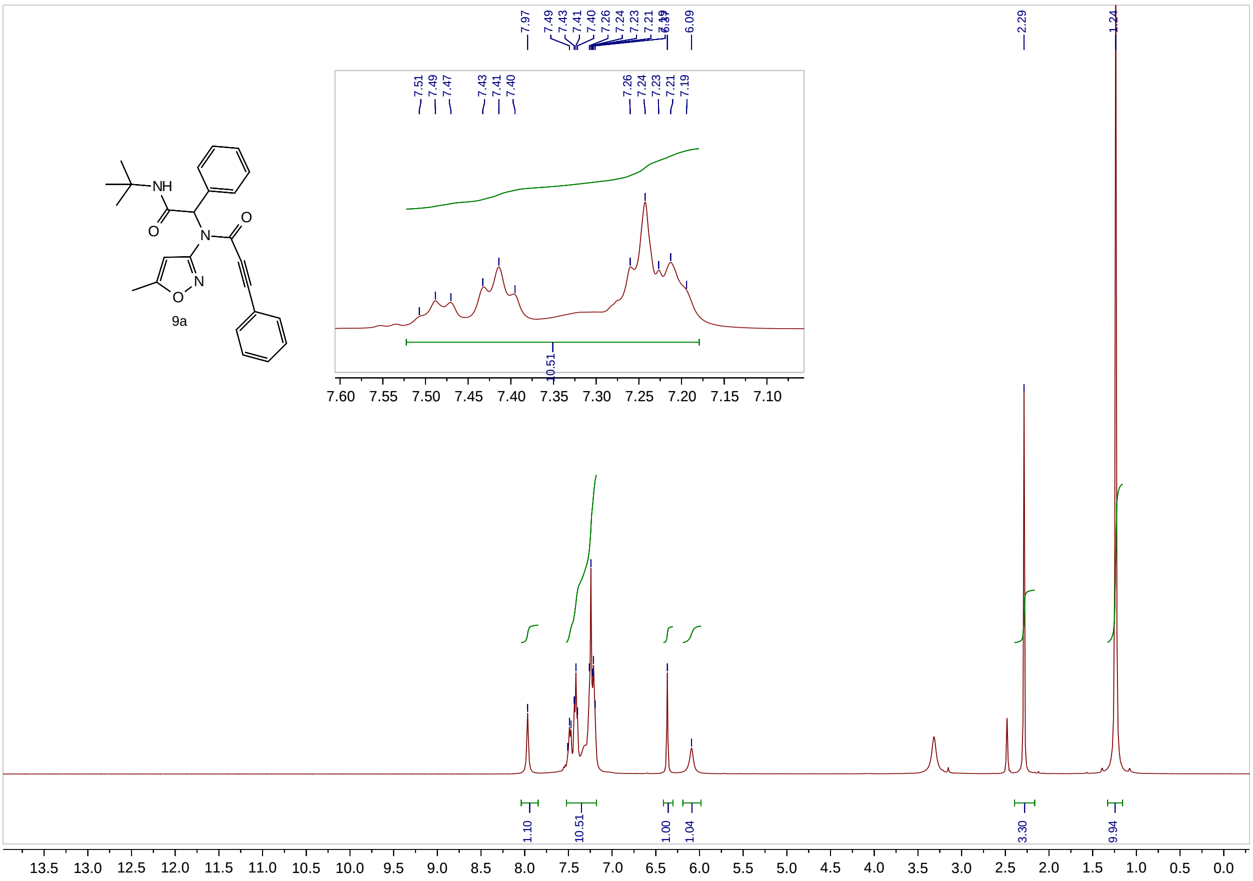

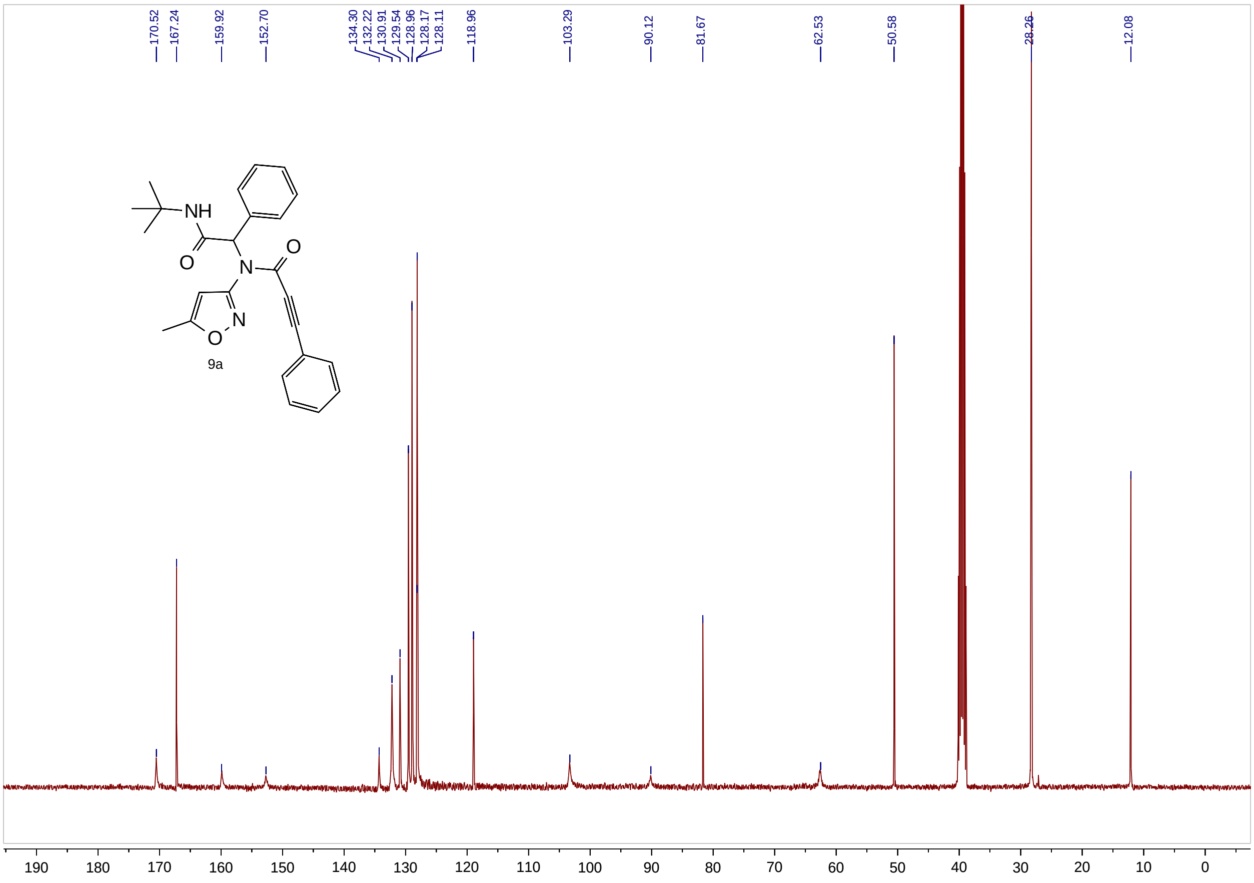

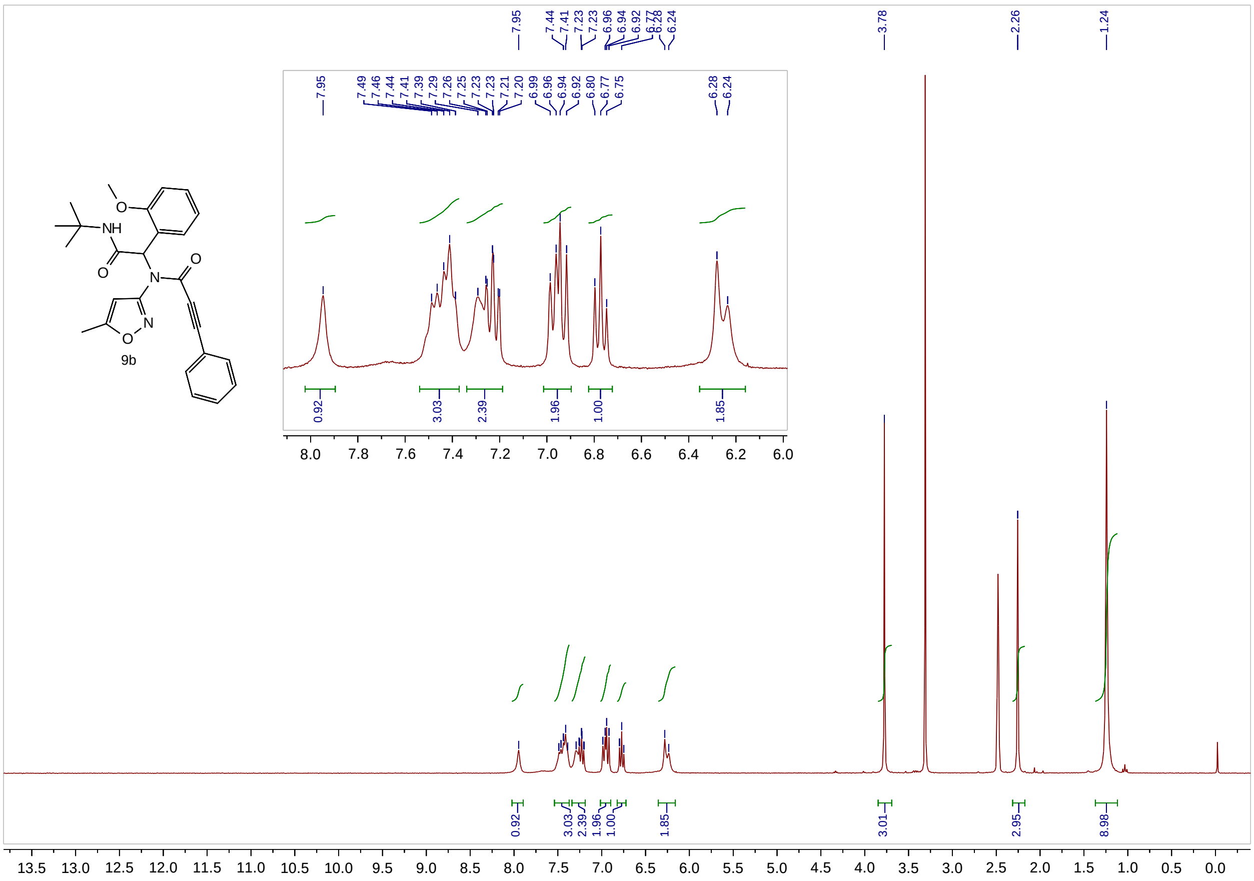
7.95
7.44
7.41
7.23
7.23
6.96
6.94
6.92
6.77
6.28
6.24
3.78
2.26
1.24
NH
O
N
O
N
O
9b
0.92
3.03
2.39
1.96
1.00
1.85
3.01
2.95
8.98
13.5
13.0
12.5
12.0
11.5
11.0
10.5
10.0
9.5
9.0
8.5
8.0
7.5
7.0
6.5
6.0
5.5
5.0
4.5
4.0
3.5
3.0
2.5
2.0
1.5
1.0
0.5
0.0

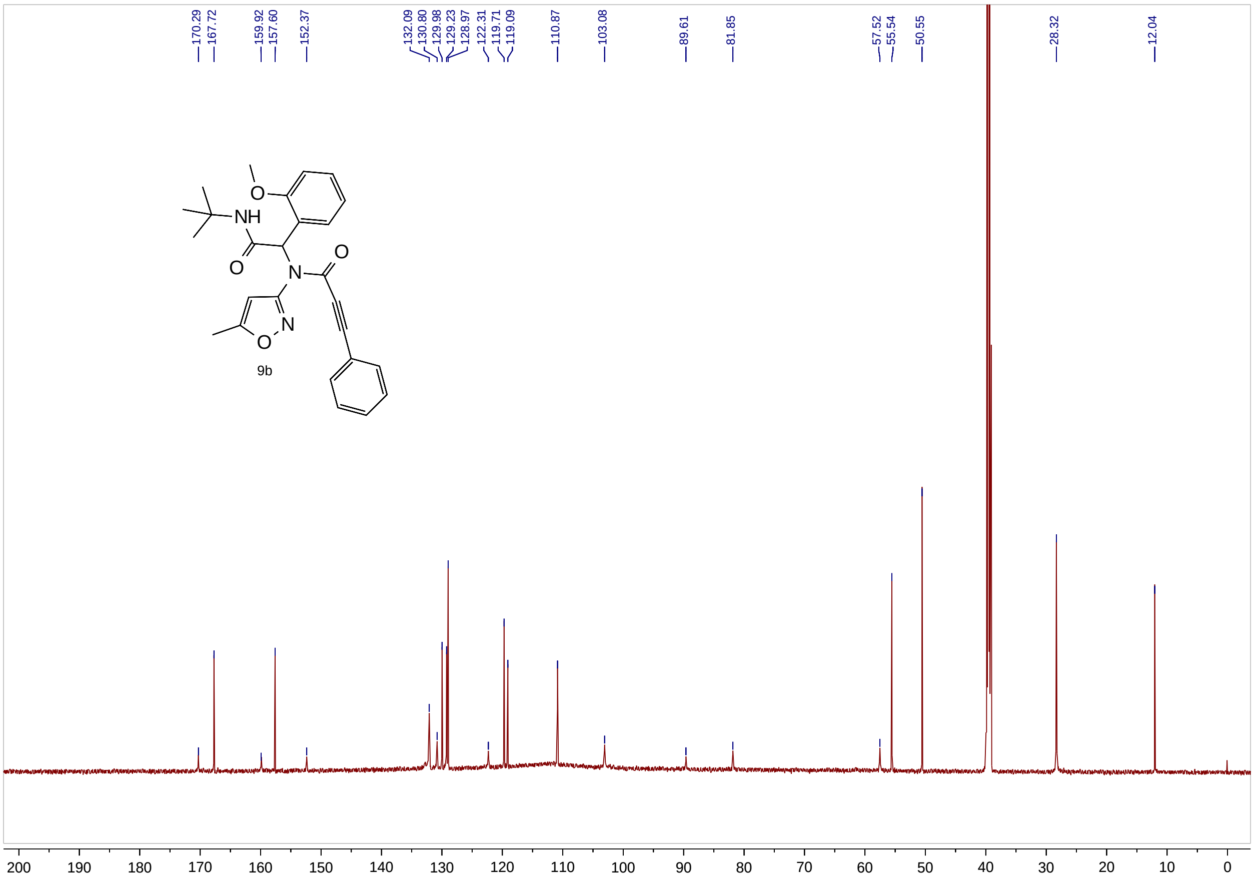
170.29
167.72
159.92
157.60
152.37
132.09
130.80
129.98
129.23
128.97
122.31
119.71
119.09
110.87
103.08
89.61
81.85
57.52
55.54
50.55
28.32
12.04
NH
O
N
O
N
O
9b
200
190
180
170
160
150
140
130
120
110
100
90
80
70
60
50
40
30
20
10
0

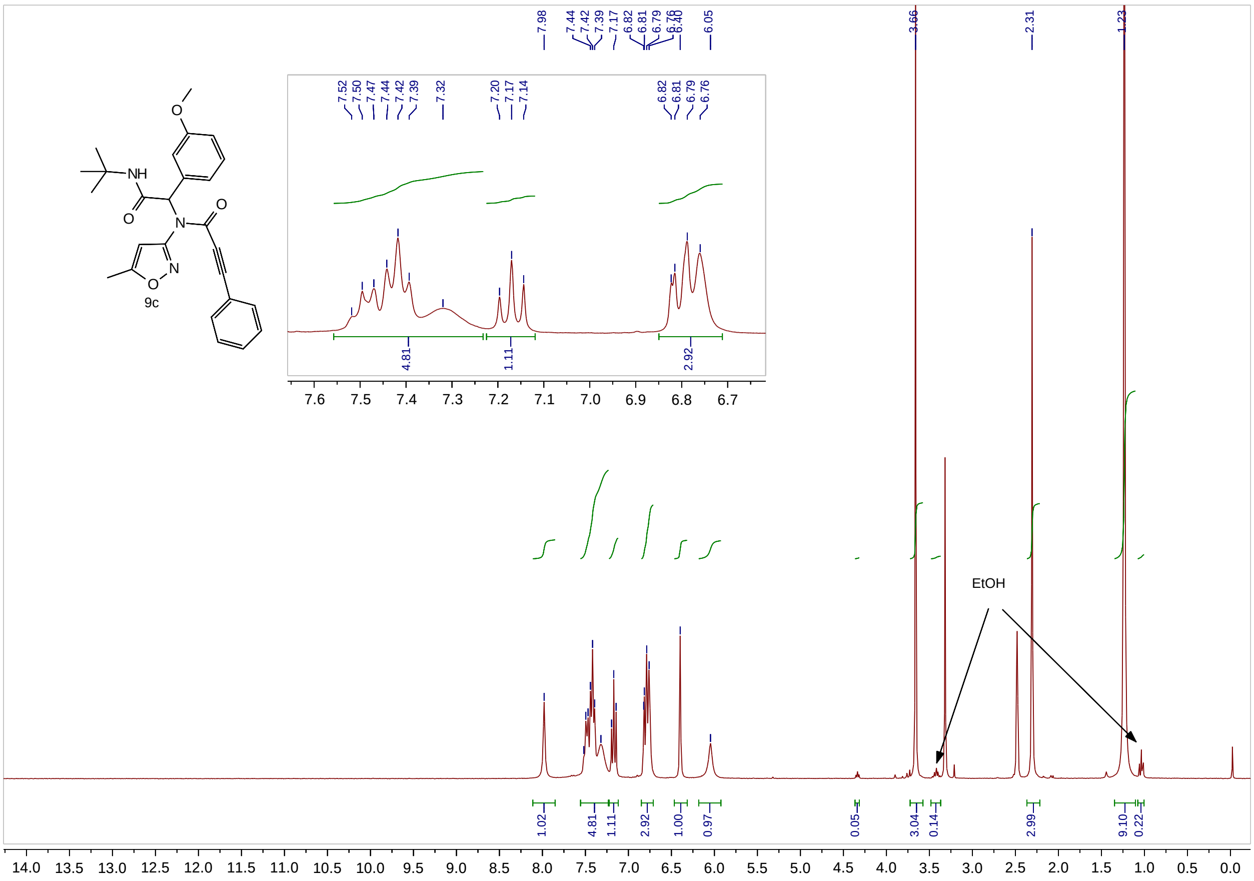
7.98
7.44
7.42
7.39
7.17
6.82
6.81
6.79
6.40
6.05
3.66
2.31
1.23
7.52
7.50
7.47
7.44
7.42
7.39
7.32
7.20
7.17
7.14
6.82
6.81
6.79
6.76
4.81
1.11
2.92
NH
O
O
N
O
N
O
9c
EtOH
1.02
4.81
1.11
2.92
1.00
0.97
0.05
3.04
0.14
2.99
9.10
0.22

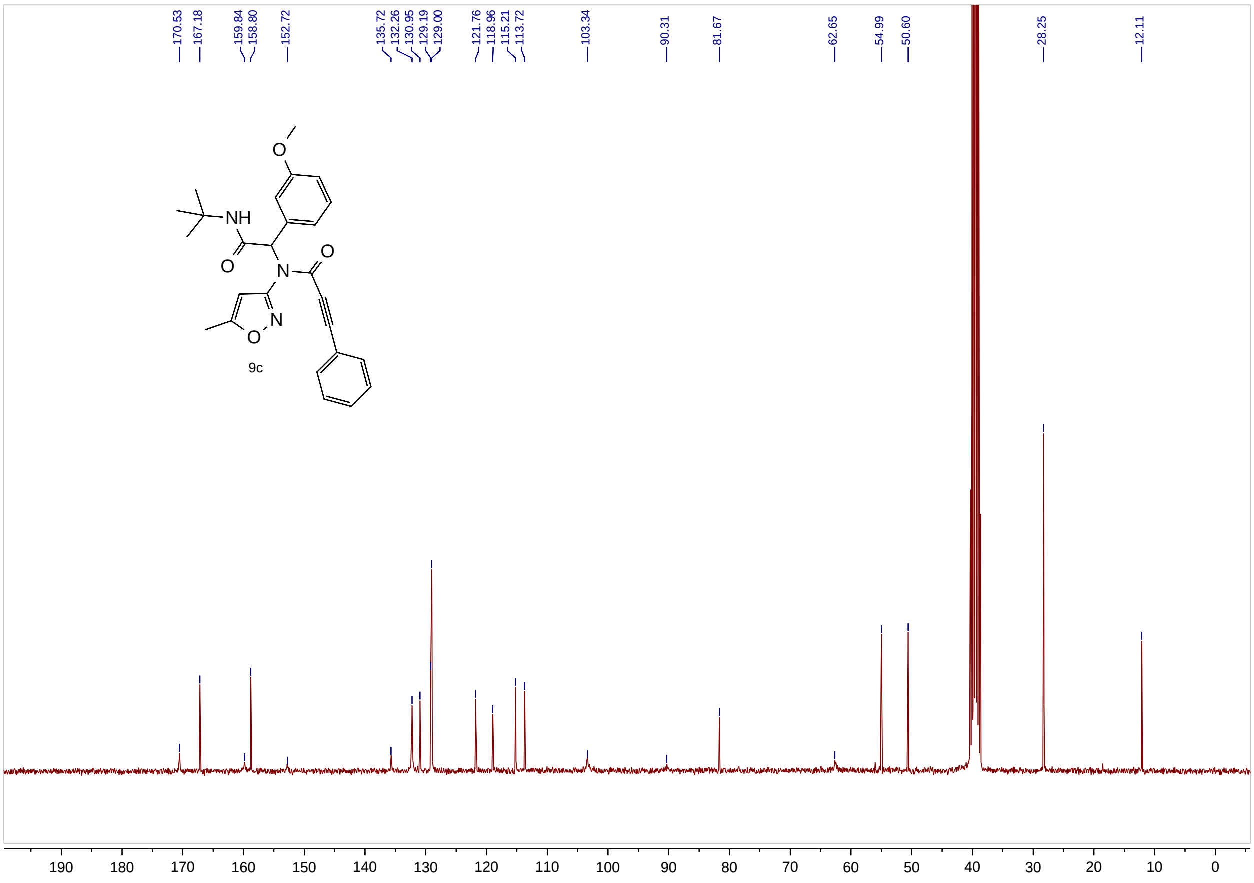
170.53
167.18
159.84
158.80
152.72
135.72
132.26
130.95
129.19
129.00
121.76
118.96
115.21
113.72
103.34
90.31
81.67
62.65
54.99
50.60
28.25
12.11
O
NH
O
N
O
N
O
9c

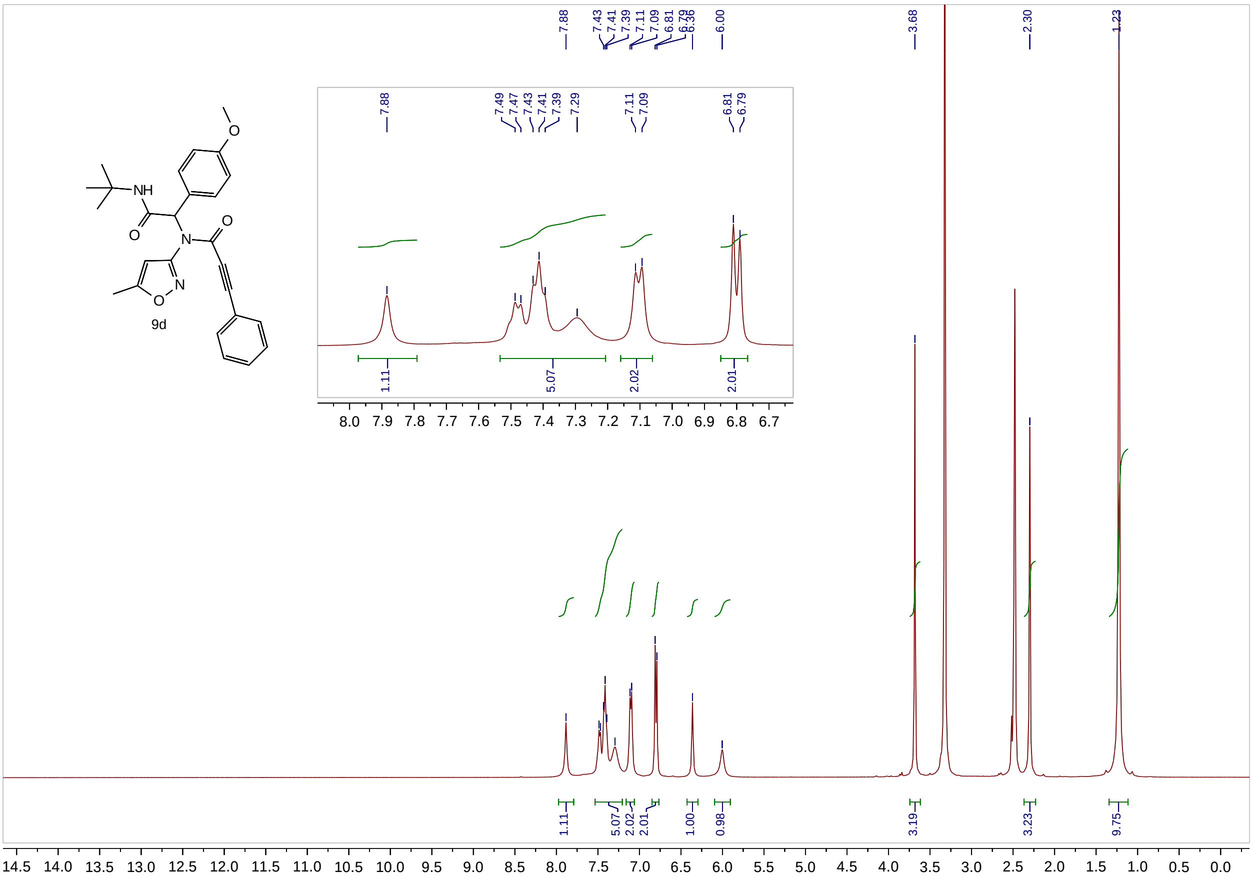
7.88
7.43
7.41
7.39
7.11
7.09
6.81
6.79
6.36
6.00
3.68
2.30
1.23
NH
O
N
O
O
N
9d
7.49
7.47
7.43
7.41
7.39
7.29
7.11
7.09
6.81
6.79
1.11
5.07
2.02
2.01
8.0 7.9 7.8 7.7 7.6 7.5 7.4 7.3 7.2 7.1 7.0 6.9 6.8 6.7
1.11
5.07
2.02
2.01
1.00
0.98
3.19
3.23
9.75
14.5 14.0 13.5 13.0 12.5 12.0 11.5 11.0 10.5 10.0 9.5 9.0 8.5 8.0 7.5 7.0 6.5 6.0 5.5 5.0 4.5 4.0 3.5 3.0 2.5 2.0 1.5 1.0 0.5 0.0

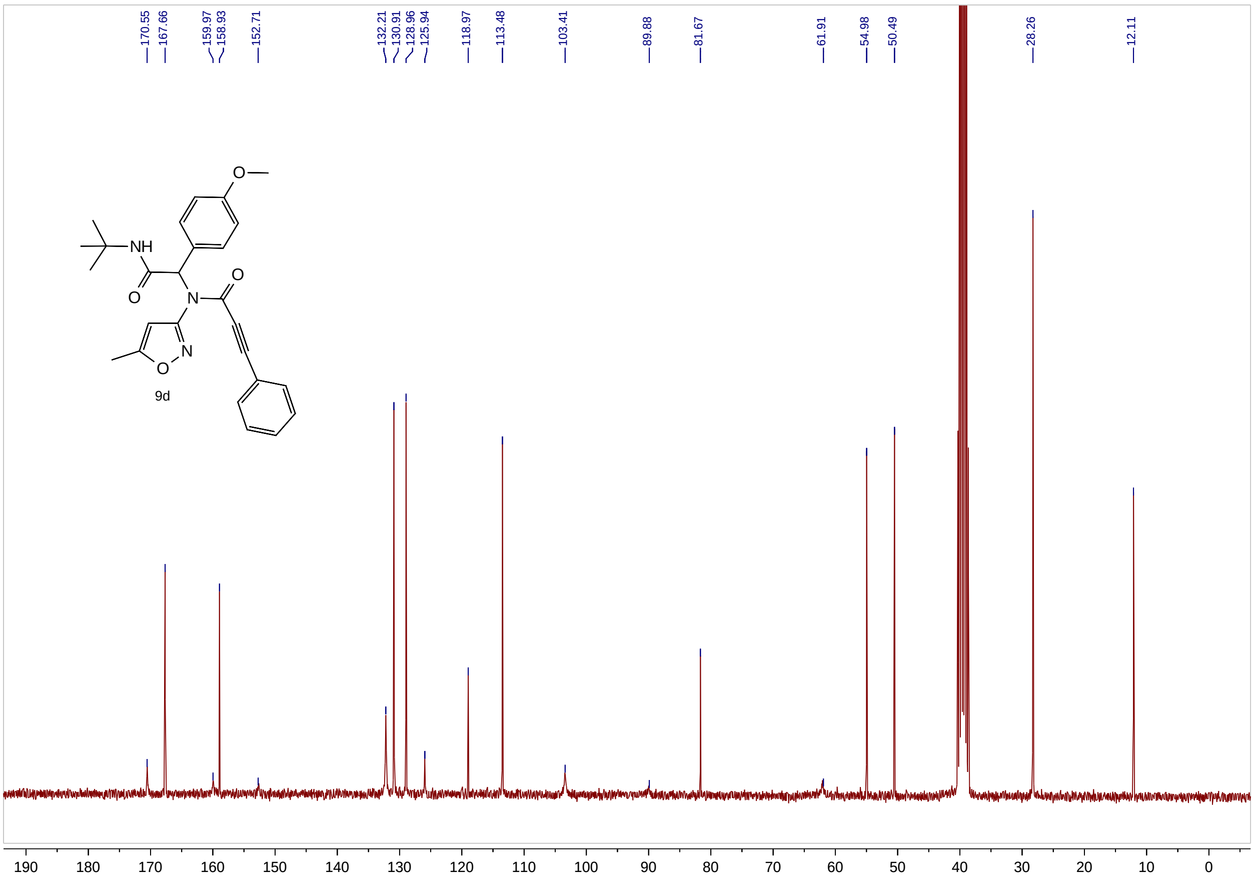
170.55
167.66
159.97
158.93
152.71
132.21
130.91
128.96
125.94
118.97
113.48
103.41
89.88
81.67
61.91
54.98
50.49
28.26
12.11
O
NH
O
N
O
O
N
9d
190 180 170 160 150 140 130 120 110 100 90 80 70 60 50 40 30 20 10 0

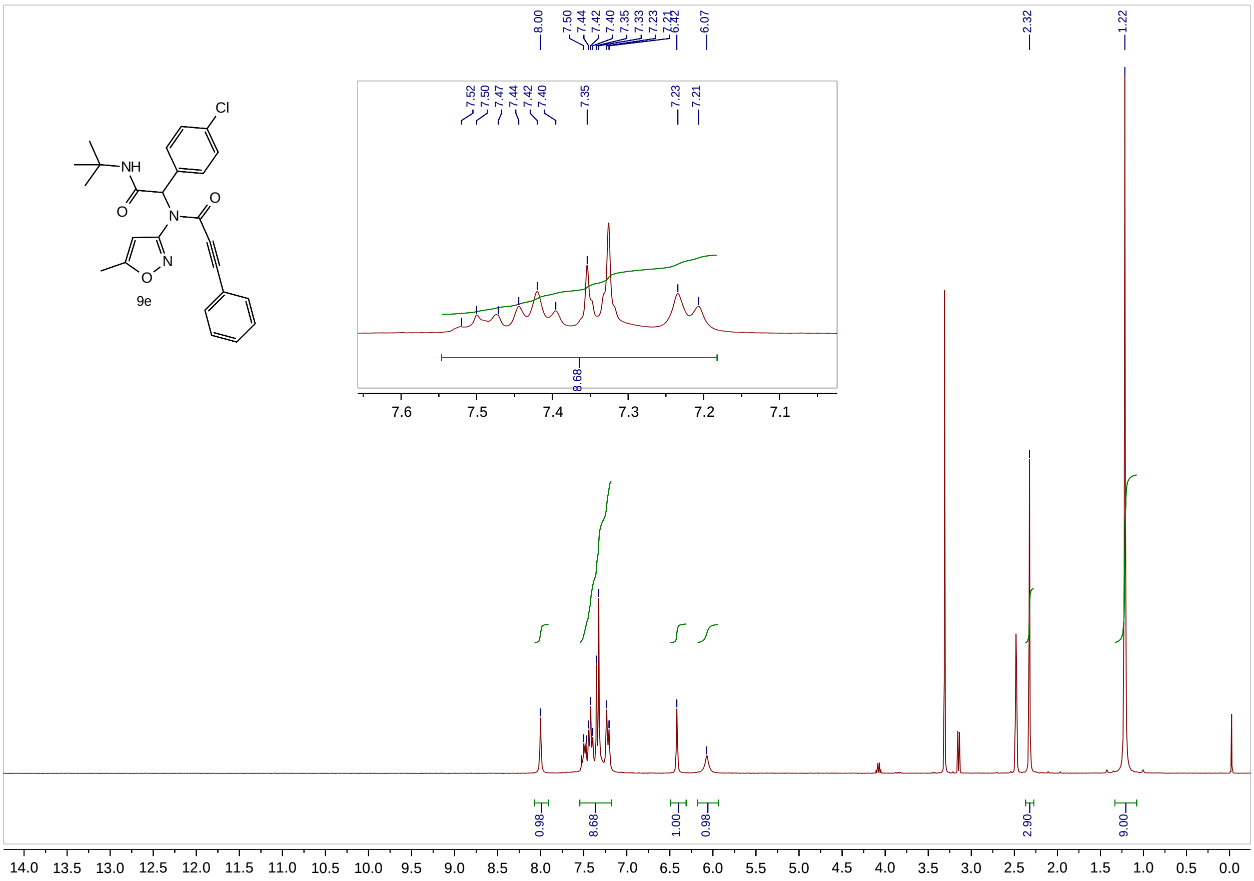
Cl
NH
O
N
O
N
O
9e
8.00
7.50
7.44
7.42
7.40
7.35
7.33
7.23
7.21
6.42
6.07
2.32
1.22
7.52
7.50
7.47
7.44
7.42
7.40
7.35
7.23
7.21
8.68
7.6
7.5
7.4
7.3
7.2
7.1
0.98
8.68
1.00
0.98
2.90
9.00
14.0
13.5
13.0
12.5
12.0
11.5
11.0
10.5
10.0
9.5
9.0
8.5
8.0
7.5
7.0
6.5
6.0
5.5
5.0
4.5
4.0
3.5
3.0
2.5
2.0
1.5
1.0
0.5
0.0

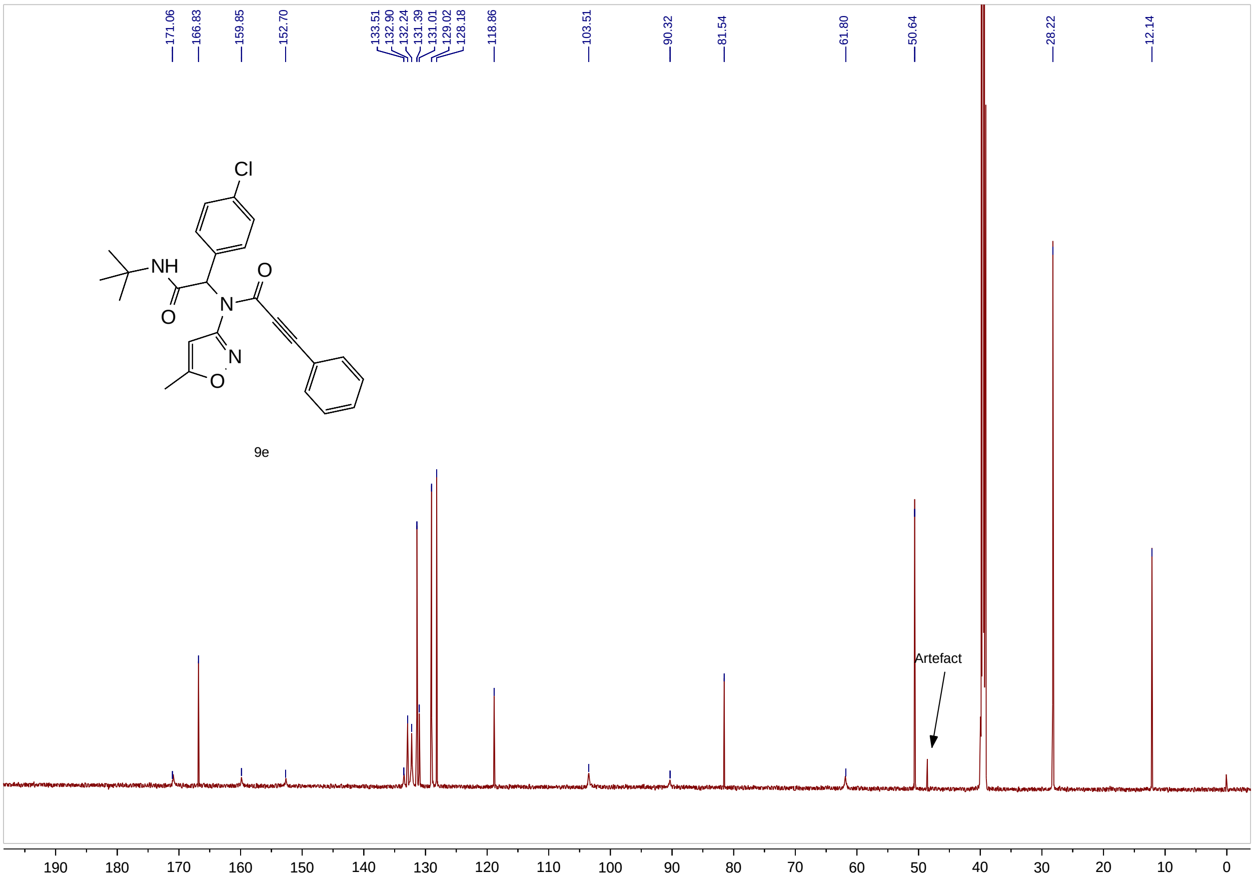
171.06
166.83
159.85
152.70
133.51
132.90
132.24
131.39
131.01
129.02
128.18
118.86
103.51
90.32
81.54
61.80
50.64
28.22
12.14
Cl
NH
O
N
O
N
O
9e
Artefact
190
180
170
160
150
140
130
120
110
100
90
80
70
60
50
40
30
20
10
0

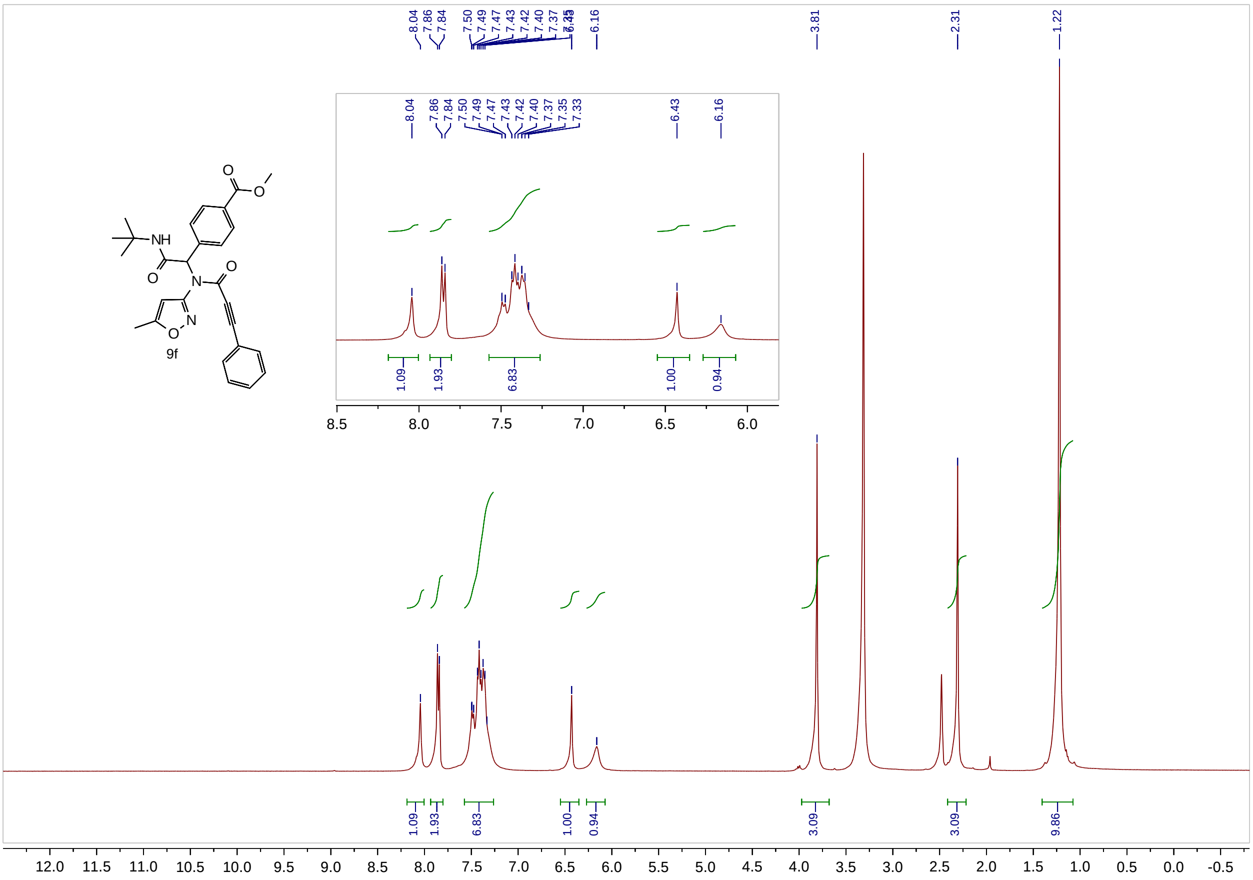
8.04
7.86
7.84
7.50
7.49
7.47
7.43
7.42
7.40
7.37
7.35
7.33
6.43
6.16
3.81
2.31
1.22
NH
O
N
O
O
O
N
9f
1.09
1.93
6.83
1.00
0.94
3.09
3.09
9.86

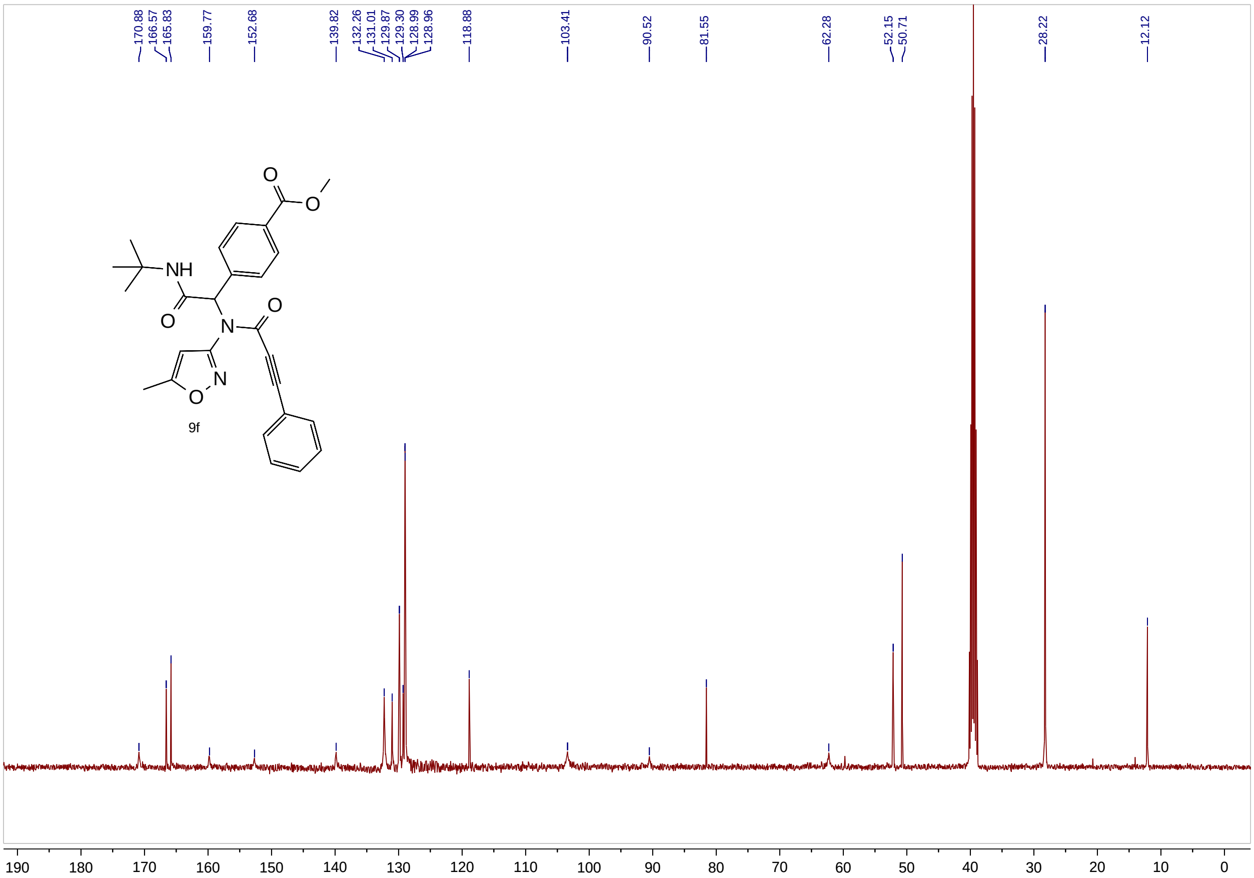
170.88
166.57
165.83
159.77
152.68
139.82
132.26
131.01
129.87
129.30
128.99
128.96
118.88
103.41
90.52
81.55
62.28
52.15
50.71
28.22
12.12
O
O
NH
O
N
O
O
N
9f

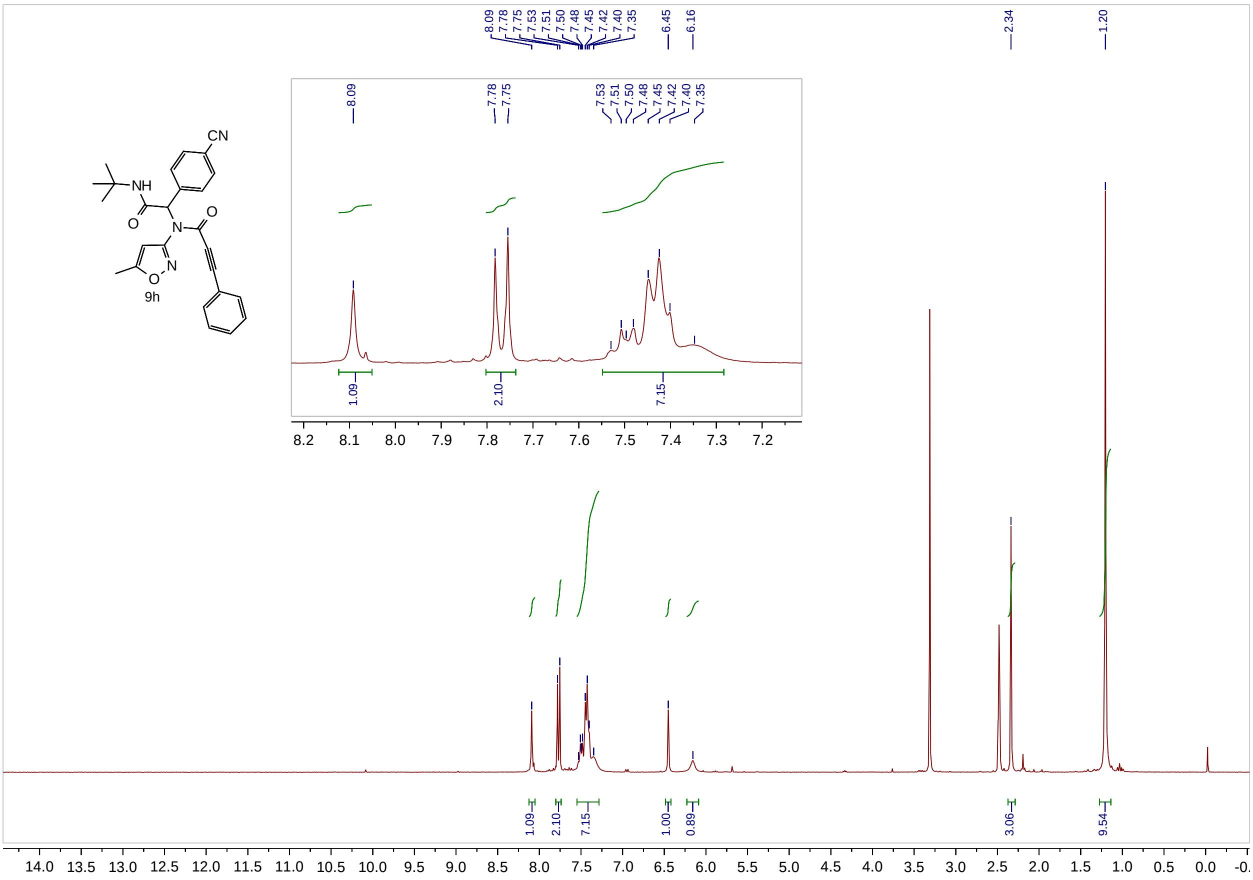

NOESY spectrum for 4a

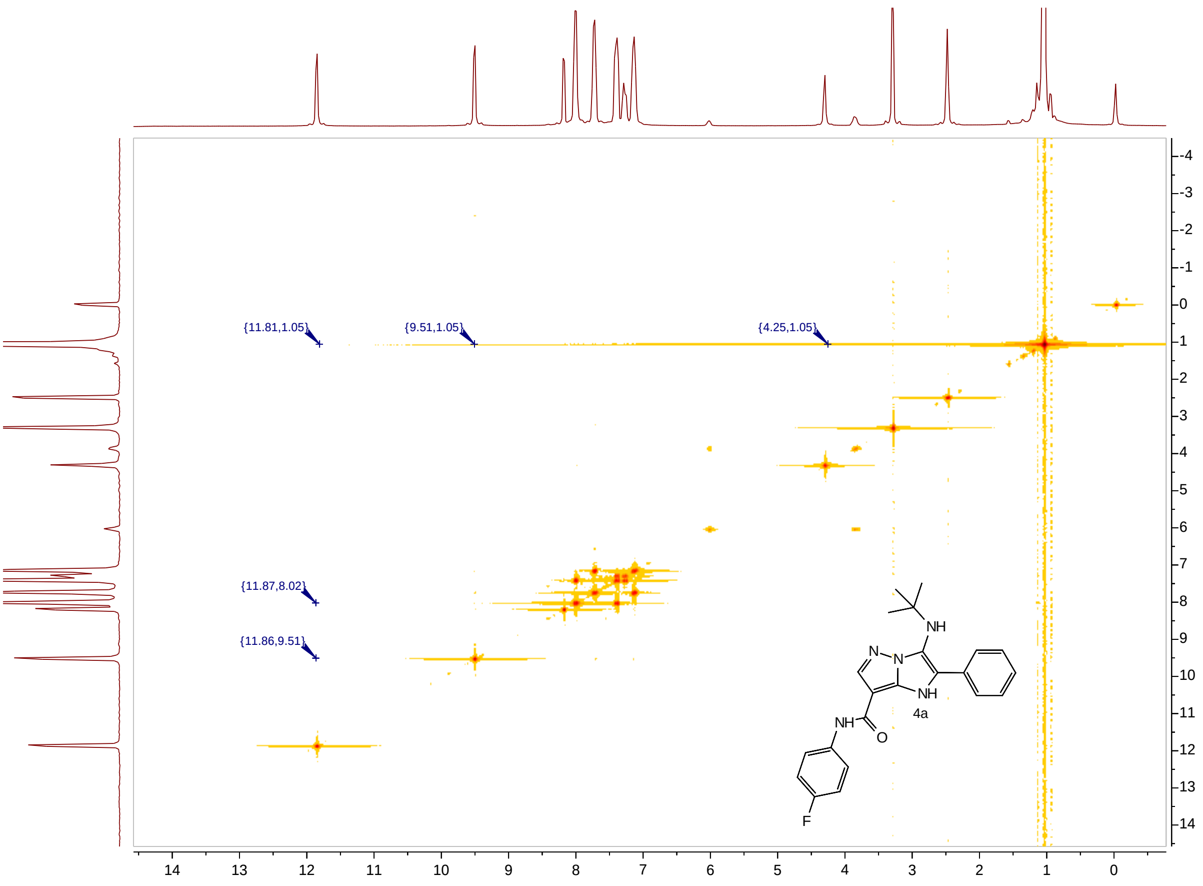

HSQC spectrum for 4a

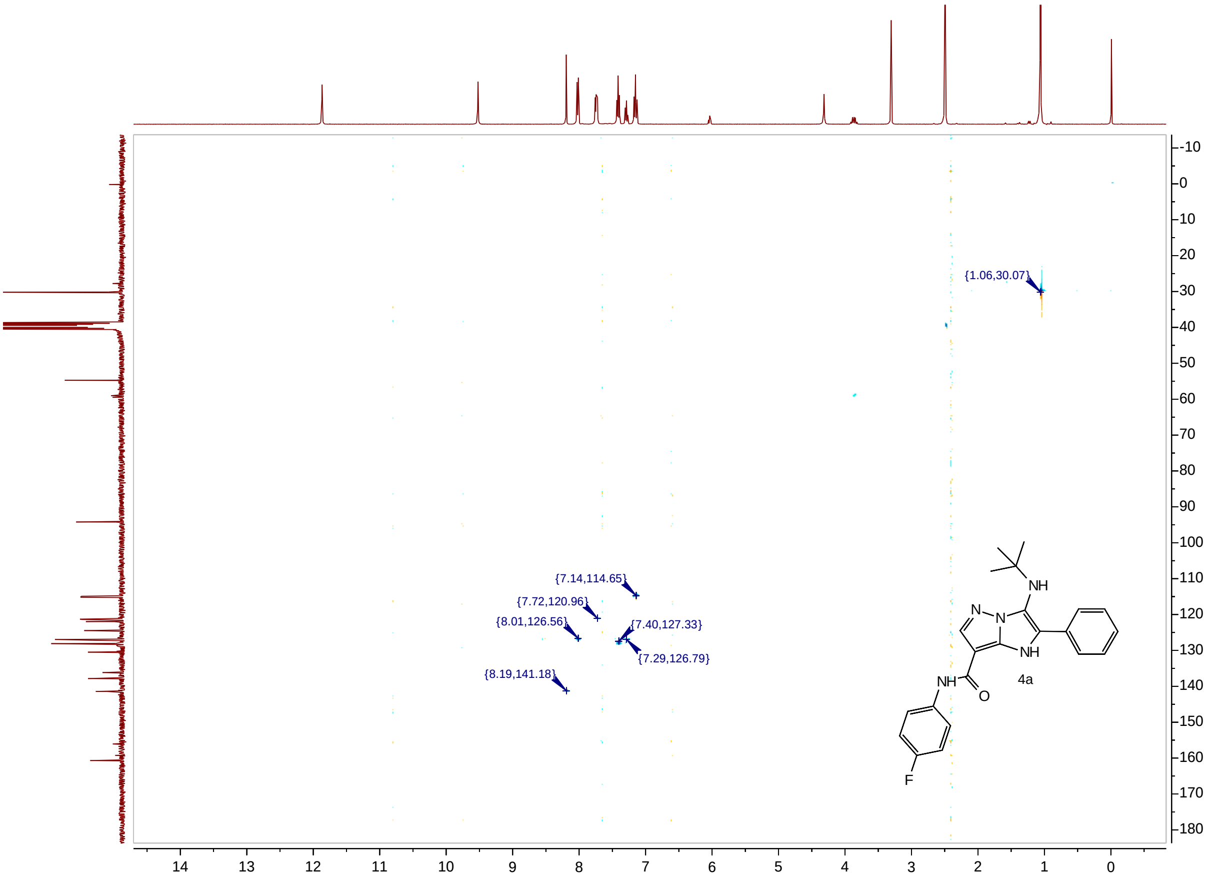

HMBC spectrum for 4a

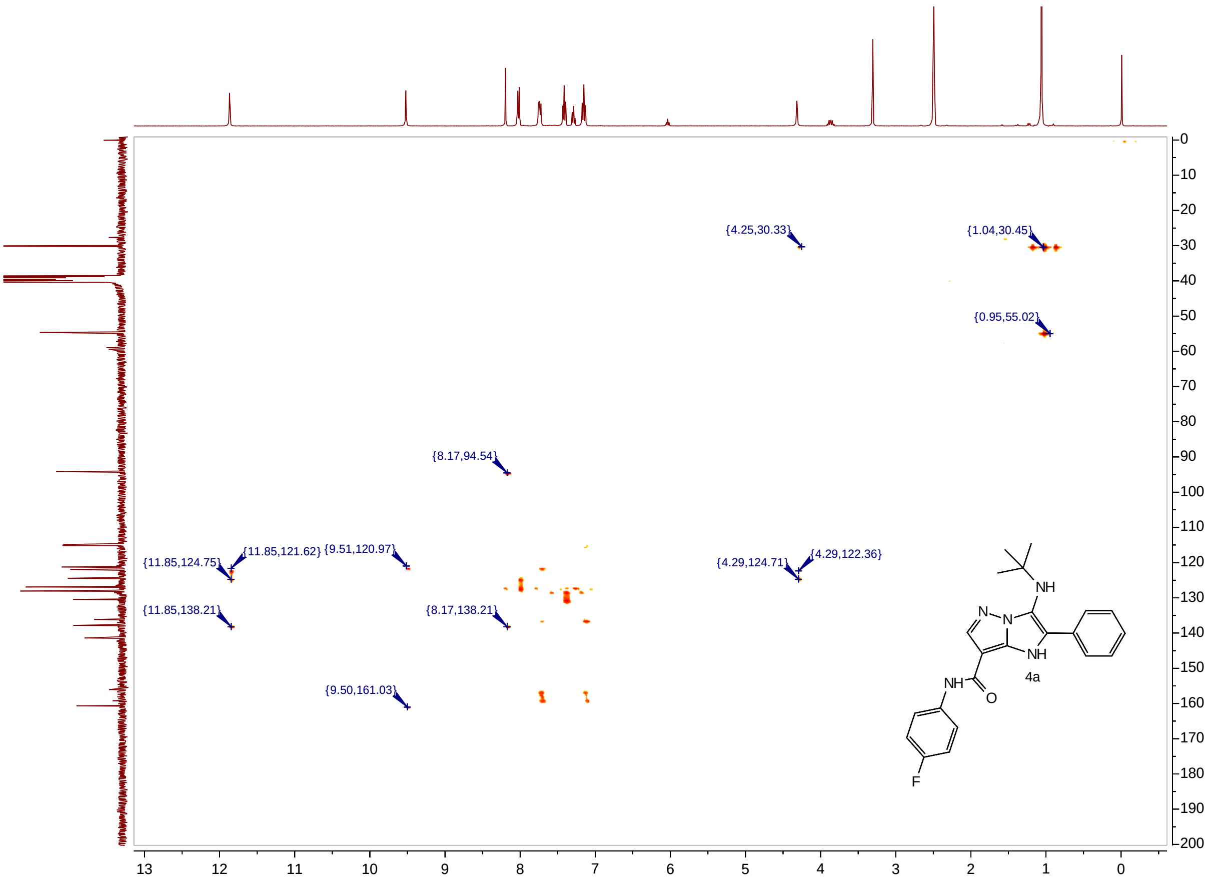

HSQC spectrum for 9b

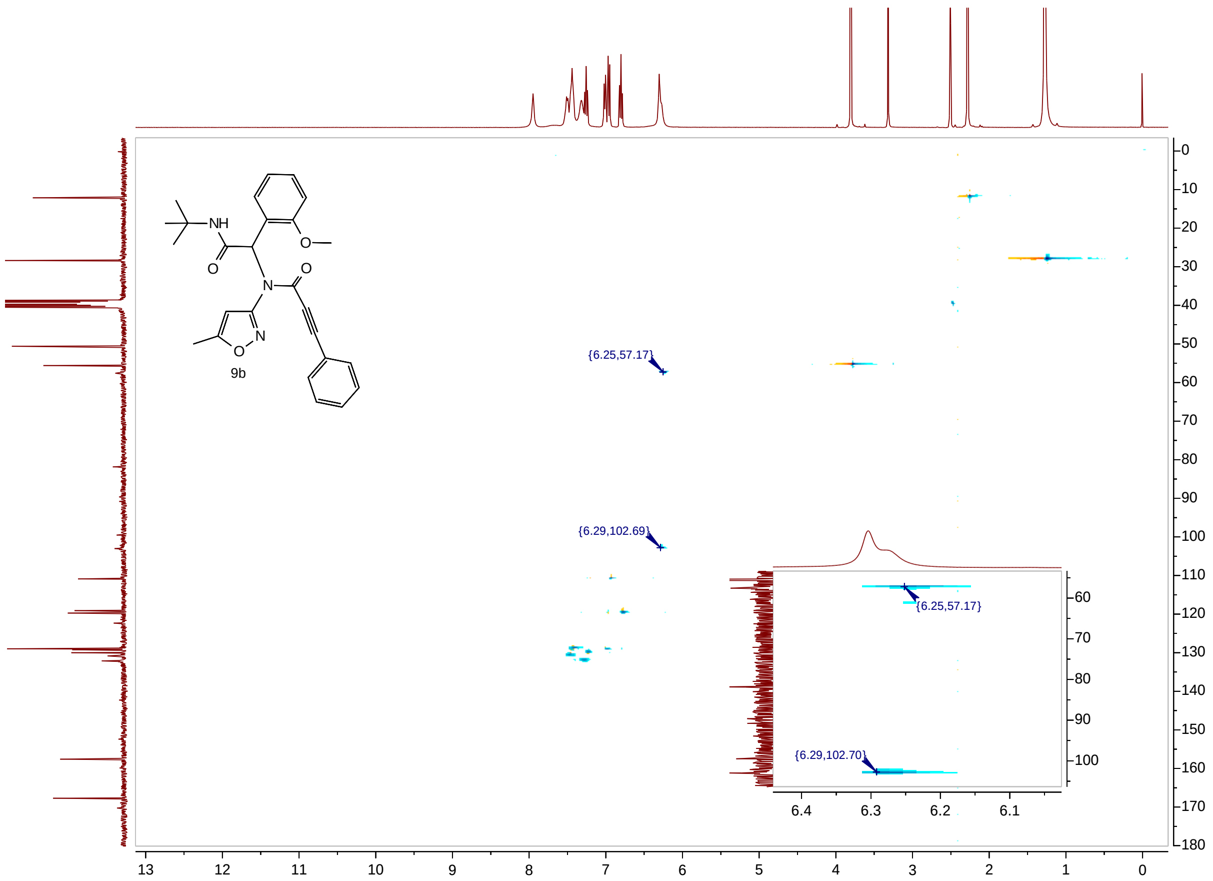

HMBC spectrum for 9b

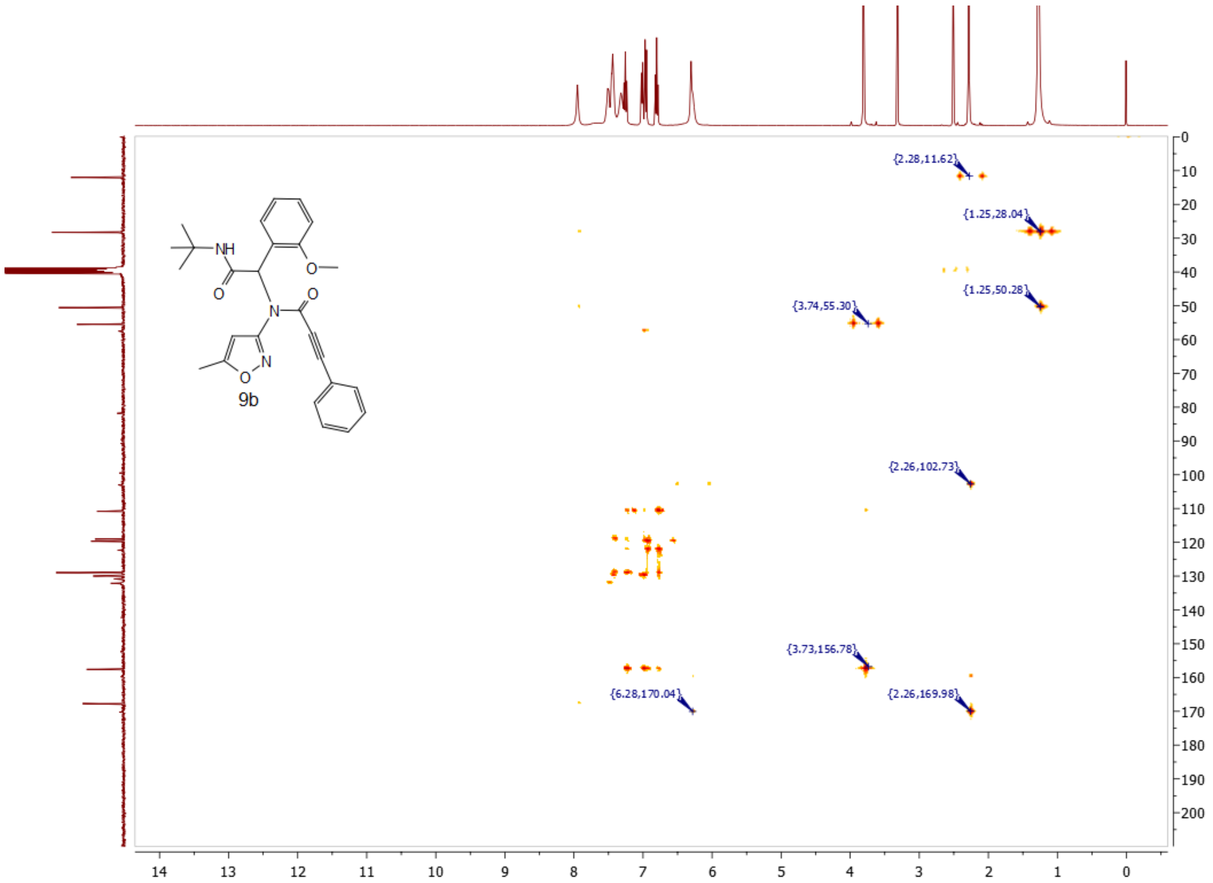

Supplement: File 2 — NMR spectra. [file Beilstein_J_Org_Chem-13-1050-s002.pdf]
